# Antidepressant Effects of South African Plants: An Appraisal of Ethnobotanical Surveys, Ethnopharmacological and Phytochemical Studies

**DOI:** 10.3389/fphar.2022.895286

**Published:** 2022-06-29

**Authors:** Melia Bokaeng Bonokwane, Makhotso Lekhooa, Madeleen Struwig, Adeyemi Oladapo Aremu

**Affiliations:** ^1^ Unit for Environmental Sciences and Management, Faculty of Natural and Agricultural Sciences, North-West University, Mmabatho, South Africa; ^2^ Preclinical Drug Development Platform, Faculty of Health Sciences, North-West University, Potchefstroom, South Africa; ^3^ Indigenous Knowledge Systems Centre, Faculty of Natural and Agricultural Sciences, North-West University, Mmabatho, South Africa

**Keywords:** alkaloids, Asteraceae, herbal medicine, ethnobotany, psychoactive plants, mental-health, indigenous knowlegde

## Abstract

Globally, the search for safe and potent natural-based treatment for depression is receiving renewed interest given the numerous side-effects associated with many existing drugs. In South Africa, the use of plants to manage depression and related symptoms is fairly documented among different ethnic groups. In the current study, we reviewed existing ethnobotanical, ethnopharmacological and phytochemical studies on South African medicinal plants used to manage depression. Electronic databases were accessed for scientific literature that meets the inclusion criteria. Plants with ethnobotanical evidence were subjected to a further pharmacological review to establish the extent (if any) of their effectiveness as antidepressants. Critical assessment resulted in 20 eligible ethnobotanical records, which generated an inventory of 186 plants from 63 plant families. Due to the cultural differences observed in the definition of depression, or lack of definition in some cultures, most plants are reported to treat a wide range of atypical symptoms related to depression. *Boophone disticha*, *Leonotis leonurus* and *Mentha longifolia* were identified as the three most popular plants, with over eight mentions each from the ethnobotanical records. The dominant families were Asteraceae (24), Fabaceae (16), Amaryllidaceae (10), and Apocynaceae (10) which accounted for about 32% of the 186 plants. Only 27 (≈14.5%) of the plants have been screened for antidepressant activity using *in vitro* and *in vivo* models. *Agapanthus campanulatus*, *Boophone disticha*, *Hypericum perforatum*, *Mondia whitei* and *Xysmalobium undulatum*, represent the most studied plants. Phytochemical investigation on nine out of the 27 plants revealed 24 compounds with antidepressant-like effects. Some of these included buphanidrine and buphanamine which were isolated from the leaves of *Boophone disticha*, Δ^9^-tetrahydrocannabinol, cannabidiol and cannabichromene obtained from the buds of *Cannabis sativa* and carnosic acid, rosmarinic acid and salvigenin from *Rosmarinus officinalis*, A significant portion (≈85%) of 186 plants with ethnobotanical records still require pharmacological studies to assess their potential antidepressant-like effects. This review remains a valuable reference material that may guide future ethnobotanical surveys to ensure their robustness and validity as well as database to identify promising plants to screen for pharmacology efficacy.

## Introduction

Clinical depression represents one of the most prevalent and highly comorbid psychiatric conditions that causes a negative emotional experience associated with pathophysiological changes (Saki et al., 2014). In this review, we will differentiate between clinical depression (MDD) and the ailments/symptomology described as depression amongst communities (MDD-com). MDD is a mental health disorder characterized by a persistent depressed mood and loss of interest in activities once enjoyed, causing noticeably significant changes in the patient’s life (Mungai and Bayat, 2019). MDD brings about physiological, behavioral and psychological symptoms initiated through stressful life situations that are difficult to overcome (Martins and Brijesh, 2018; Fathinezhad et al., 2019). Symptoms of MDD may include a depressed mood, pervasive lack of energy, a change in sleeping patterns and psychomotor activity, changes in appetite and/or weight, cognitive disturbances that make it difficult to think, concentrate, or make decisions, feeling worthless, guilt, and repeated thoughts of death and suicidal attempts or idealizations (American Psychiatric Association, 2000; World Health Organisation, 2017). MDD is caused by imbalances with certain neurotransmitters in the central nervous system (Munir et al., 2020). According to Schildkraut, (1965), the most studied basis of the pathophysiology of MDD is the alteration of monoaminergic activity in the brain, which leads to the depletion of serotonin, dopamine and norepinephrine neurotransmitters. The noradrenaline and serotonin systems play an important role in stress response, the regulation of emotions and the neurobiology of depression (Ressler and Nemeroff, 2000). Antidepressants are the current MDD treatment focusing on improving monoamine activity, however, they are associated with adverse side effects and a delayed response time (Saki et al., 2014; Popa-Velea et al., 2015), leading to poor clinical outcomes.

MDD is one of the three leading causes of disease burden in South Africa and globally (Nglazi et al., 2016). The WHO ranked it as the largest contributor to global disability, with over an estimated 300 million people suffering from MDD globally in 2015 (World Health Organisation, 2017). It is projected that by 2030, MDD will be considered the global disease burden based on serious limitation on existing treatment methods owed to the therapeutic success, safety and efficacy of available antidepressants (Abbas et al., 2015). According to the South African Stress and Health (SASH) study, MDD is among the mental disorders with the highest lifetime prevalence in South Africa (Herman et al., 2009). MDD is more prevalent in females than in males (World Health Organisation, 2017). The correlation between hormonal changes in women and an increased prevalence of depression suggests that fluctuations in hormones during puberty, menstruation, after pregnancy and around menopause may trigger depression in women (Albert, 2015). In most developing countries, there is a limited access to treatment for mental disorders, especially in primary health care settings (Stafford et al., 2008; Ferrari et al., 2013). As a result, the communities have often used plants to alleviate some of the symptomology associated with depression or feeling unwell. Following the development of the WHO Traditional Medicine Strategy 2014–2023 and due to the legal acknowledgement of traditional healers, the South African government, as a member state of the WHO, developed legislation and policies to facilitate the institutionalization of traditional medicine in South Africa (Mothibe and Sibanda, 2019).

MDD-com includes mental health related problems that could be classified as depression based on similar clinical symptoms and is perceived by African traditional healers as caused by ancestors or bewitchment (Sorsdahl et al., 2009). When interviewed on the understanding of depression, one participant (traditional healer) from a South African study conducted by Starkowitz, (2013) stated that “traditionally, there is not something like depression,” while another participant replied “we don’t have the word depression.” Those with some degree of understanding of the concept defined MDD-com as “an experience of “feeling sick” inward and feeling down in your heart while the outside flesh will be feeling fine,” and “an experience that makes a person feel like lying down and covering themselves with a blanket” (Starkowitz, 2013). Several states, such as “being put down” by ancestors, experiencing headaches and migraines, being possessed by evil spirits, mourning, sorrow and being inflicted with curses often resemble a depressed and hopeless state (Stafford et al., 2008). Multiple somatic complaints such as headaches and fatigue represent the most common presentations of MDD-com in Zimbabwe (Todd et al., 1999). Wei et al. (2016) reported that the prevalence rate of depression in patients with headache was 19.7%, and that about 15.2% of the patients had headache due to somatic symptoms of depression (cause of headache) and 10.9% attributed their primary headache as a comorbidity of depression. Moreover, depression increased the risk of tension-type headache in study participants following laboratory stress (Janke et al., 2004). This overlap between depression and headache provides evidence of a link between medicinal plants used traditionally for headache and potential antidepressant properties. Particularly, appropriate utilization of disease categories and classification allow for cross-culture comparisons (Staub et al., 2015). Therefore, the emic perception and categorization of diseases (which usually comes from within a culture) has to be understood for the development of a culturally appropriate classification system (Heinrich et al., 2009).

The use of traditional medicine is widespread in South Africa, where an estimated 70–84% of the black population consult traditional healers at some point (Koen et al., 2003; Robertson, 2006; Stafford et al., 2008). In addition, more people often utilize traditional healers than medical practitioners for their primary health care (Robertson, 2006). This can be attributed to preferences, the increased demand for herbal products in preventative health care and general well-being, and the easy availability of the cheaper, individualized and culturally appropriate traditional healthcare system (Mander, 1998; Viljoen et al., 2019). The use of commercially processed herbal preparations as part of traditional medicine, and the sale thereof, has increased over the last decade (Ndhlala et al., 2011). The use of herbal medicine such as psychotropic plant extracts is common amongst patients with mood-like and anxiety-like disorders, including MDD (Sarris et al., 2011). *Boophone disticha* is one of the most popular medicinal plants in South Africa where bulb infusions of this plant are used to treat headache and mental condition (Hutchings et al., 1996; Sobiecki, 2002). *Sceletium tortuosum* was used in the prehistoric times as a mood-altering substance, and the plant is chewed or drank traditionally for depression and stress (Hutchings et al., 1996; Van Wyk et al., 1997; Van Wyk and Gericke, 2000; Sobiecki, 2002). Infusions made from leaf, fruit and leaf decoctions of *Schinus molle* are used as antidepressants (Bhat and Jacobs, 1995). Several medicinal herbs are being used to treat mental disorders including depression (Fathinezhad et al., 2019). *Hypericum perforatum* L. is being used to treat mild depression as an alternative to conventional synthetic antidepressants (Nielsen et al., 2004), while *Sceletium tortuosum* (L.) N.E. Br has been developed into various pharmaceutical products for mood elevation (Stafford et al., 2008).

Despite the anecdotal and long-term use of medicinal plants for the treatment of MDD and related ailments in South Africa, an in-depth and up-to-date appraisal on this subject is lacking. Moreover, the need to bridge the cross-cultural gap observed in the definition of depression by exploring the regional, cultural and/or traditional concepts of depression as understood by South African traditional healers cannot be overemphasized. This review focuses on the ethnobotanical studies on South African plants with potential antidepressant effects, their pharmacological (*in vitro* and *in vivo*) screening and phytochemical assessment. This study is envisaged to provide an in-depth and current state of knowledge in the search for South African plants with antidepressant potential and identify critical gaps for future research direction in the on-going global search for safe and efficient antidepressants.

## Materials and Methods

### Literature Search Strategy

A web-based systematic literature search was conducted from March to November 2021 to identify ethnobotanical information on medicinal plants used traditionally in South Africa to treat MDD and MDD-com. The systematic review was conducted according to the PRISMA guidelines for reporting systematic reviews and meta-analysis (Moher et al., 2009; Moher et al., 2015). Electronic databases such as the Web of Science, MEDLINE (PubMed), ScienceDirect and Google Scholar were searched for published and unpublished scientific literature, including journal articles, books, theses and dissertations, on South African medicinal plants used to treat clinical depression and related ailments. These databases were searched using keywords/phrases such as South African medicinal plants, antidepressant effects of medicinal plants, ethnobotany of South Africa, indigenous plant use, medicinal plant use, South African psychoactive plants, Zulu medicinal plants. In addition, literature was retrieved from the library of the North-West University (NWU), South Africa. All medicinal plants identified by this search were subject to a further literature review to establish the extent (if any) of pharmacological research conducted into the efficacy of the plants as antidepressants. The electronic databases mentioned above were used to search for pharmacological studies providing supporting evidence of the antidepressant-like effects for each plant species, both *in vitro* and *in vivo*. To filter these studies, search terms “antidepressant effect of plants” and other keywords relating to depression and specific monoamines involved, together with plant species names, were used.

### Eligibility Criteria

The screening of all search results involved reviewing the title and abstract of articles and identifying and selecting eligible publications, downloading identified research articles, and critically assessing the articles on how they met the inclusion criteria.

#### Inclusion Criteria

For a research article to be included in the review it must:

• Be a published ethnobotanical survey reporting potential antidepressant effects of medicinal plants
• Indicate the traditional use, safety and toxicity of medicinal plants for depression and related ailments (e.g. headache, sorrow, mourning, nervousness, stress, tension, mental illness known as “spirits,” alcoholism, insomnia, insanity, feeling like crying) in South Africa
• Be published or made available on the internet during the research period (i.e. up to November 30, 2021)

For Ethno-Pharmacological Studies

• Plant extracts must be investigated against either Serotonin reuptake transporter (SERT), Dopamine transporter (DAT) and Noradrenalin transporter (NAT) receptors *in vitro*.
• Evaluate the effect of plant extract on behavioral markers of depression in vivo and the safety/toxicity profile.

#### Exclusion Criteria

Research articles were excluded from the review if they:

• Ethnobotanical review articles (literature or systematic)
• Focus on natural resources (other than plants) used for depression
• Ethnobotanical surveys that is not focusing on South Africa
• Have limited data (e.g. missing scientific plant names) on the medicinal use of plants for depression

### Data Collection

Relevant data on the antidepressant-like effects of South African medicinal plants were extracted to Excel spreadsheets following the pre-defined criteria. Bibliographies from accessed articles, together with their citations, were downloaded and saved on an online reference manager (EndNote). Missing information from some articles (e.g. local names, life form of the plant, and misspelt scientific names) and in cases of research papers lacking geographic locations of the study, the data was retrieved through direct web searching (Google). Scientific names of plants, family, local names, life form of plants, method of preparation of the plant-based medicine, route of administration and short notes were collected for each plant from each article. Where possible, data on the ethnic group that uses the plants traditionally were collected. Plant species and family were validated in references to The World Flora Online (http://theworlflora.online) and PlantZAfrica (http://pza.sanbi.org/) while the local names were confirmed using PlantZAfrica (http://pza.sanbi.org/). Any botanical synonyms or unaccepted names were updated to the recent updated plant nomenclature and are reported as such in this review. The plants listed in this review are arranged in alphabetical order based on plant families and scientific names.

## Results

### Literature Search Results

**FIGURE 1:**
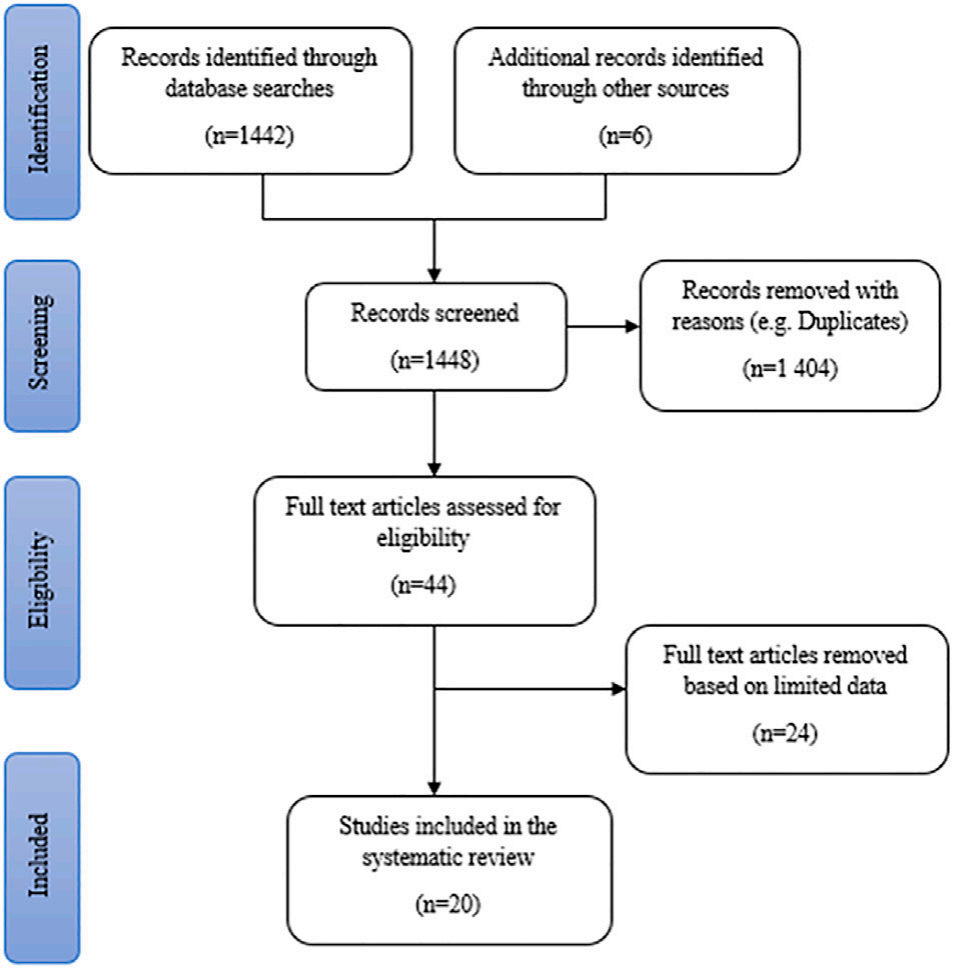
Search strategy results for the identification of studies included in the systematic review.

A total of 1,442 publication records were identified from online database searches. Additional data were obtained from six literature sources (books) retrieved from the NWU library, making a total of 1,448 records that were screened. After the removal of duplicates, 44 articles and books were assessed for eligibility and explored relative to the inclusion criteria. The full text of 44 studies was reviewed in detail and 24 studies were removed due to limited data and other reasons stated in the exclusion criteria. Finally, 20 studies were reviewed for the documentation of the potential antidepressant-like effects of medicinal plants (Figure 1). The literature comprises of a total of 15 area specific ethnobotanical surveys (75%) and five plant inventories (25%). These studies covered six out of the 9 (approximately 67%) provinces in South Africa, with multiple studies being conducted in KwaZulu-Natal and Western Cape (both four studies). The most common method of data collection across the eligible articles was semi-structured and structured interviews (40% of the literature). Most of the literature reviewed covers a wide range of medicinal uses for each plant. However, for the purpose of this review, only the plant medicinal uses related to depression and associated symptoms were listed in the generated plant inventory.

The operational definition of MDD was inconsistent across most of the ethnobotanical literature reviewed. Most of the literature did not define MDD but a broad range of traditional medicinal uses in their surveys, rather than medicinal uses specific to MDD. Since most medicinal uses reported in the literature are indigenous knowledge gathered from South African traditional healers, records of the potential antidepressant effects of medicinal plants are listed as MDD-com, which entails ailments based largely on somatic symptoms such as headaches, fatigue, or spirits. According to Mosotho et al. (2008), there has been the misconception, until recently, that developing countries are relatively free of psychiatric problems, such as MDD, which are encountered in industrialized nations. Mosotho et al. (2008) also highlighted that MDD may be easily misdiagnosed since physical complaints, such as headaches, are not always recognized as a manifestation of depression. This lack of inconsistency between western and indigenous disease classification makes the evaluation of medicinal plant use in a western scientific setting more difficult (Staub et al., 2015; Weckerle et al., 2018).

**FIGURE 2:**
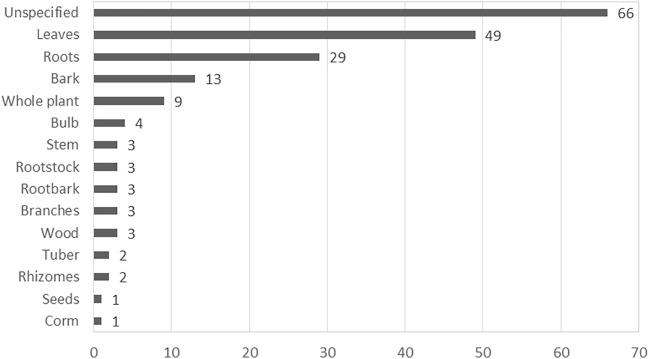
The frequency of medicinal plant parts used in South Africa for depression and related ailments.

**FIGURE 3:**
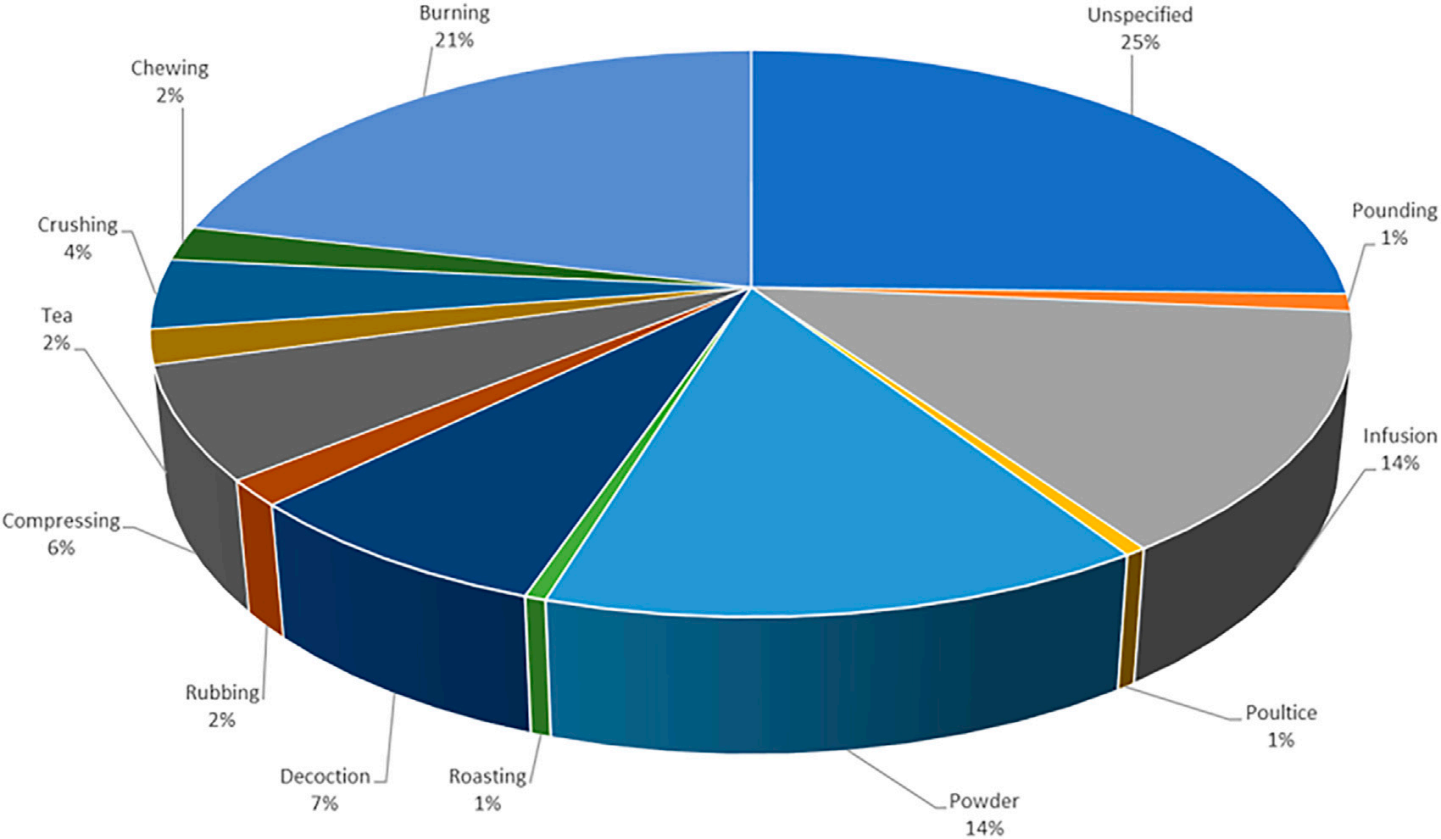
Different methods of preparation for medicinal plants (*n* = 215). Out of the 186 medicinal plants recorded, several had ˃1 method used for preparation.

The absence of methodological framework was evident in one of the reviewed study (Moteetee et al., 2019). A number of concerns were observed with the naming of the listed plants which are often encountered in published articles (Rivera et al., 2014). About 50% (10) of the articles listed at least one scientific name incorrectly either by misspelling the scientific name, recording an incorrect or ambiguous scientific name or by using non-standard author abbreviation (Van Wyk et al., 1997; Van Wyk and Gericke, 2000; Van Wyk et al., 2008; Mogale et al., 2015; Hulley and Van Wyk, 2019). In approximately 65% of the reviewed studies, there was no evidence of the identity of the medicinal plant recorded as the authors failed to provide details of the voucher specimen of the plants. Failure to provide sufficient details on the identification of the voucher specimen often makes the validation of the plant species difficult (Weckerle et al., 2018). Although some authors specified the plant parts used for the herbal preparation for managing depression and related ailments, this information was absent in many cases (Figure 2). Likewise, similar concern was evident with the method used for preparing the plants (Figure 3). These aforementioned concerns are important information required for the generation of plant inventory to ensure the integrity of data from ethnobotanical surveys.

### South African Medicinal Plants With Potential Antidepressant Effects

Table 1 represents an overview of the key characteristics of each of the ethnobotanical studies reviewed. Included studies were conducted and published during the period from 1989 to 2019. A total of eight studies were conducted in the past 7 years. In this review, 186 medicinal plants from 63 plant families have been listed as being used in South Africa to treat MDD and MDD-com (Supplementary Table S1). The life forms for the plants included shrubs (40%), trees (21%), herbs (38%) and grasses (1%). An overview of 54 medicinal plants that comprised of popular plants based on multiple mentions (37) from ethnobotanical studies and 27 plants with pharmacological evidence for depression are listed in Table 2. About 20% of the recorded plants (i.e. *Adenia gummifera* (Harv.) Harms, *Albizia adianthifolia* (Schum.) W.Wight, *Ballota hirsuta* Benth., *Boophone disticha*, *Gomphocarpus fruticosus* (L.) W.T.Aiton, *Mentha longifolia* (L.) L. and *Leonotis leonurus* (L.) R.Br.) were categorized as the most popular based on a relatively high number of mentions in the ethnobotanical surveys reviewed. The three most popular plants, with over eight mentions each from ethnobotanical surveys, included *Boophone disticha*, *Leonotis leonurus* and *Mentha longifolia*. Several other plants, such as *Agapanthus campanulatus, Cannabis sativa*, *Heteromorpha trifoliata* (H.L.Wendl.) Eckl. and Zeyh., *Sceletium tortuosum*, *Schinus molle* L. and *Xysmalobium undulatum*, have been used traditionally in South Africa to treat MDD and MDD-com (Hutchings et al., 1996; Van Wyk et al., 1997; Van Wyk and Gericke, 2000).

**TABLE 1:** An overview of ethnobotanical literature documenting South African medicinal plants with potential antidepressant effects.

Reference	Province	Area/region	Title/focus of the study	Ethnic group	No. of plant species	No. of plant families	Voucher specimen deposited	Characteristics of participants	Methodological framework (data collection and analysis, techniques)
Van Wyk and Gericke, (2000)	Southern Africa	Unspecified	An inventory of useful plants of Southern Africa	Unspecified	12	9	Unspecified	Unspecified	Ethnobotanical book
Van Wyk et al. (2008)	Eastern Cape and Western Cape	South-eastern Karoo, Graaff-Reinet and Murraysburg regions	An ethnobotanical survey of medicinal plants used in the southeastern Karoo	Xhosa, Khoikhoi and San	8	5	Unspecified	Local experts	Ethnobotanical field studies
Nortje and van Wyk, (2015)	Northern Cape	Kamiesberg, Namaqualand	An ethnobotanical survey of medicinal plants of the Kamiesberg, Namaqualand, South Africa	Khoisan	12	11	Yes	Local inhabitants	Semi-structured and structured interviews
Hutchings, (1989)	KZN	Unspecified	A survey and analysis of traditional medicinal plants as used by the Zulu, Xhosa and Sotho	Zulu, Xhosa and Sotho	40	22	Unspecified	Unspecified	Ethnobotanical field studies
Hutchings, et al. (1996)	KZN	Unspecified	An inventory of Zulu medicinal plants	Zulu	54	31	Unspecified	Traditional healers	Ethnobotanical book
Van Wyk et al. (1997)	Unspecified	South Africa	Inventory of medicinal plants of South Africa	Unspecified	27	19	Unspecified	Unspecified	Ethnobotanical book
Sobiecki, (2002)	Unspecified	Unspecified	A preliminary inventory of plants used for psychoactive purposes in southern African healing traditions	Unspecified	40	26	Unspecified	Traditional healers	Interviews
Corrigan et al. (2011)	KZN	KwaNibela Peninsula, St Lucia	An ethnobotanical survey of plants used in the KwaNibela Peninsula, St Lucia, South Africa	Zulu and Swati	2	2	Unspecified	Community members and traditional knowledge experts	Ethnobotanical field studies
Bhat and Jacobs, (1995)	Eastern Cape	Transkei	An ethnobotanical survey of traditional herbal medicine used in Transkei	Xhosa	3	3	Yes	Elderly villagers, traditional doctors and herbalists	Interviews
Stafford, (2009)	KZN and Western Cape	Unspecified parts of KZN and Western Cape	Ethnobotanical literature survey of South African medicinal plants with central nervous system related activity and use	Zulu	34	23	Unspecified	Unspecified	Ethnobotanical field studies
Moffett, (2016)	Lesotho and Free State	Unspecified	An ethnobotanical survey of medicinal plants used by the Basotho	Sotho	45	23	Unspecified	Traditional healers	Interviews
Venter and Venter, (2016)	Unspecified	Unspecified	A list of useful South African indigenous trees	Unspecified	11	8	Unspecified	Unspecified	Ethnobotanical book
De Beer and Van Wyk, (2011)	Northern Cape	Agter–Hantam	An ethnobotanical survey of the Agter–Hantam, Northern Cape Province, South Africa	Khoi–San	7	6	Yes	Traditional healers and local people of Khoi–San descent	Interviews
Hulley and Van Wyk, (2019)	Western Cape	Western Little Karoo/Kannaland (Barrydale, Zoar, Calitzdorp and Vanwyksdorp)	Quantitative medicinal ethnobotany of Kannaland (western Little Karoo), South Africa	Khoi-San	21	16	Yes	Children (13–19 y/o), adults (20–59 y/o) and senior citizens(60 y/o and above) of Khoi descent	Ethnobotanical field survey
Mogale et al. (2019)	Limpopo	Central Sekhukhuneland (Frisgewaght at Phokwane, Ga-Moretsele/Tsehlwaneng near Jane Furse and Ga-Sekhele near Schoonoord)	The ethnobotany of Sekhukhuneland and the plants used by rural Bapedi people	Pedi	3	2	Unspecified	Twenty-seven local inhabitants	Ethnobotanical field surveys and interviews
Moteetee et al. (2019)	Lesotho and the Free State	Unspecified	The ethnobotany of the Basotho of Lesotho and the Free State Province of South Africa (South Sotho)	Sotho	17	13	Unspecified	Unspecified	Unspecified
Philander, (2011)	Western Cape	Unspecified	An ethnobotany of Western Cape Rasta bush medicine	Khoi-San, Rastafari	18	15	Yes	Bush doctors	Ethnobotanical field survey, interview
Mongalo, (2018)	Limpopo	Blouberg area	Ethnobotanical knowledge of the lay people of Blouberg area (Pedi tribe), Limpopo Province, South Africa	Pedi	2	2	Yes	Traditional healers and medicinal plant sellers	Ethnobotanical field survey, questionnaires
Thring and Weitz, (2006)	Western Cape	Bredasdorp/Elim region of the Southern Overberg	Medicinal plant use in the Bredasdorp/Elim region of the Southern Overberg in the Western Cape Province of South Africa	Coloured population	10	5	Yes	Elderly people	Interviews, questionnaires
Mhlongo and Van Wyk, (2019)	KZN	Amandawe	Zulu medicinal ethnobotany: new records from the Amandawe area of KwaZulu-Natal, South Africa	Zulu	2	2	Unspecified	Community members	Ethnobotanical field survey

KZN, KwaZulu-Natal.

**TABLE 2:** Examples of popular medicinal plants used against depression and related ailments in South Africa based on multiple mentions in ethnobotanical literature and the presence of pharmacological evidence.

Plant family	Scientific name [synonyms]	Local name^#^	Life form	Plant part used	Method of preparation, route of administration and/or short notes	References
Aizoaceae	*Sceletium tortuosum* (L.) N.E. Br [*Mesembryanthemum tortuosum* L.]	Kanna (E); Kougoed (A)	Herb	Whole plant	Used as a psychoactive substance; Emetics made from leaves in boiling water are administered for the fearful dreams; Leaves used to treat headache; Whole plant chewed or drunk for depression and anxiety disorders; Past use as a mood-altering substance from prehistoric times; The dried plant material is prepared traditionally and chewed, smoked, or powdered and inhaled as a snuff; Whole plant used to elevate mood and reduce anxiety and stress	Hutchings et al. (1996); Nortje and van Wyk, (2015); Philander, (2011); Sobiecki, (2002); Van Wyk and Gericke, (2000); Van Wyk et al. (1997)
Amaryllidaceae	*Agapanthus campanulatus* F.M. Leight. [*A. campanulatus* subsp*. patens* (F.M. Leight.) F.M. Leight.]	Bell agapanthus (E); Bloulelie (A); Ubani (Z); Leta-la-phofu (S); Ugebeleweni (X)	Herb	Unspecified	Unspecified parts used by the Sotho to treat people with “spirit,” which is a type of mental disturbance; Unspecified	Moffett, (2016); Sobiecki, (2002); Stafford, (2009)
	*Boophone disticha* (L.f.) Herb. [*Amaryllis disticha* L.f., *Brunsvigia disticha* (L.f.) Sweet*, B. toxicaria* (L.f. ex Aiton) Herb.]	Cape poison bulb, sore eye flower (E); Gifbol, seeroogblom (A); Leshoma (S); Incwadi (X); Incotho (Z)	Herb	Bulb	Used as emetics and snuffed or inhaled medicines; Bulb decoctions are administered by mouth to adults suffering from headaches; Unspecified; Unspecified; Bulb infusions are drunk to induce hallucinations and to treat mental diseases; Unspecified; Bulbs are used to treat headache; Weak decoctions of bulb scales administered by mouth or as enemas for headache	Hutchings, (1989); Hutchings et al. (1996); Moffett, (2016); Philander, (2011); Sobiecki, (2002); Stafford, (2009); Van Wyk and Gericke, (2000); Van Wyk, et al. (1997)
	*Haemathus coccineus* L. [*H. latifolius* Salisb.]	March flower, paintbrush lily (E); Bergajuin, bloedblom (A); Uzaneke (Z)	Herb	Roots	Boiled root decoctions are taken as emetics	Hutchings et al. (1996)
	*Scadoxus puniceus* (L.) Friis and Nordal [*Haemanthus puniceus* L, *H. rouperi* auct. *H. superbus* Baker]	Paintbrush lily (E); Rooikwas (A); Umgola (Z)	Shrub	Bulbs	Bulbs are used for headaches; Unspecified	Hutchings et al. (1996); Van Wyk, et al. (1997)
Anacardiaceae	*Schinus molle* L. [*S. angustifolia* Sessé and Moc., *S. huigan* Molina, *S. molle* var*. molle*, *S. occidentalis* Sessé and Moc.]	False pepper tree (E); Peperboom (A)	Tree	Stems, leaves	Infusions made from leaves and fruits and leaf decoctions are used as antidepressants; Unspecified part pressed on the head for headache; Leaves used as compress to treat headache; Fresh leaves placed on a cloth with vinegar and wrapped on the head for headache	Bhat and Jacobs, (1995); Hulley and Van Wyk, (2019); Nortje and van Wyk, (2015); Van Wyk et al. (2008)
Apiaceae	*Alepidea amatymbica* Eckl. and Zeyh. [*A. amatymbica* var *amatymbica* Eckl. and Zeyh., *A. amatymbica* var. *cordata* Eckl. and Zeyh., *A. aquatica* Kuntze, *Eryngium amathymbicum* (Eckl. and Zeyh.) Koso-Pol]	Giant alepidea (E); Kalmoes (A); Ikhathazo (Z); Iqwili (X); Lesoko (S)	Herb	Rhizome; roots	Dry rhizome and roots are smoked, or powdered and taken as a snuff to help prevent nervousness; Dry rhizomes are smoked or powdered and taken as snuff for mild sedation and vivid dreams; Fresh rhizomes are chewed, or decoctions are made from dried product. Also administered as snuff or burnt and inhaled. Smoke from roots used as a mild sedative	Sobiecki, (2002); Van Wyk and Gericke, (2000); Van Wyk et al. (1997)
	*Centella asiatica* (L.) Urb. [*C. asiatica* var. a*siatica*, *C. asiatica* var. *crista* Makino, *C. hirtella* Nannf.]	Indian pennywort (E); Inyongwane (X); Varkoortjies (A)	Herb	Leaves	Finely ground leaves used as snuff; Dried, powdered leaf used as a snuff, which produces a calming, sedative effect; Possesses anti-inflammatory, tranquilizing and age-related neuroprotective effects	Sobiecki, (2002); Van Wyk and Gericke, (2000); Van Wyk et al. (1997)
	*Heteromorpha trifoliata* (H.L.Wendl.) Eckl. and Zeyh. [*Bupleurum trifoliatum* H.L.Wendl. and Bartl.]	Parsley tree (E); Mkatlala (S); Umbangandlala (Z)	Tree	Leaves	Emetics and snuffed or inhaled medicines; Leaf decoctions are administered for mental and nervous diseases e.g. smoked for headaches; The Sotho administer leaf decoctions for mental and nervous diseases, and Xhosa administer warm leaf infusions for similar purposes	Hutchings, (1989); Hutchings et al. (1996); Sobiecki (2002)
Apocynaceae	*Gomphocarpus fruticosus* (L.) W.T.Aiton [*Asclecias fruticosa* L., *G. fruticosus* subsp. *decipiens* (N.E.Br) Goyder and Nicholas*, G. fruticosus* subsp. *flavidus* (N.E.Br) Goyder and Nicholas, *G. fruticosus* subsp. *rostratus* (N.E.Br) Goyder and Nicholas]	Milkweed (E); Tontelbos (A); Lebejana (S); Umsinga-lwesalukazi (Z)	Herb	Whole plant	Emetics and snuffed or inhaled medicines; Dried aerial parts used as snuff; Leaves are taken orally as headache treatment; Roots used as snuff to treat headache; Snuff made from powdered leaves used as a sedative; Unspecified; Snuff made from powdered leaves is used as a sedative; Snuff from powdered leaves is used as a sedative	Hutchings, (1989); Moffett, (2016); Mogale et al. (2019); Nortje and van Wyk, (2015); Stafford, (2009); Van Wyk and Gericke, (2000); Van Wyk et al. (1997)
	*Hoodia gordonii* (Masson) Sweet ex Decne. [*Scytanthus gordonii* (Masson) Hook., *Stapelia gordonii* Masson]	Bushman’s hat, Hoodia (E); Bitterghaap (A)	Shrub	Unspecified	Unspecified	Van Wyk et al. (1997)
	*Mondia whitei* (Hook.f.) Skeels [*Chlorocodon whitei* Hook. f., *C. whiteii* Hook. f.]	White’s ginger (E); Umondi (Z)	Herb	Roots	Root infusions used to treat stress and tension in adults; Unspecified	Sobiecki, (2002); Stafford, (2009)
	*Xysmalobium undulatum* (L.) W.T.Aiton [*Asclepias ciliata* Murray ex Decne., *A. leucotrica* Schltr., *A. undulata* L., *Gomphorcarpus undulatus* (L.) Schltr.]	Milk bush (E); Bitterhout/melkbos (A); Iyeza (X); Ishinga (Z); Leshokoa (S)	Herb	Roots	Emetics and snuffed or inhaled medicines; Unspecified; Used as decongestant and for headache; Roots contain several glycosides with weak central nervous system depressant and antidepressant activity; Unspecified; Powdered root used as snuff	Hutchings, (1989); Hutchings et al. (1996); Moffett, (2016); Sobiecki, (2002); Stafford, (2009); Van Wyk, et al. (1997)
Asparagaceae	*Bowiea volubilis* Harv. [*Ophiobostryx volubilis* (Harv.) Skeels, *Schizobasopsis volubilis* (Harv.) J.F.Macbr.]	Climbing onion (E); Knolklimop (A); Ugibisisila, iguleni, (Z); Umgaqana (X)	Herb	Bulb	Emetics and snuffed or inhaled medicines; Infusions made from crushed bulbs are used as protective washes when travelling; Bulb used to treat sore eyes and headache; Unspecified	Hutchings, (1989); Hutchings et al. (1996); Philander, (2011); Van Wyk, et al. (1997)
Asteraceae	*Afroaster hispida* (Thunb.) J.C.Manning and Goldblatt [*Aster bakerianus* Burtt Davy ex C.A.Sm., *A. asper* (Less.) Schönland, *A. bakerianus* subsp. *albiflorus* W.Lippert]	Baker’s wild aster (E); Udlutshana (Z); Umthekisana (X); Phoa (S)	Herb	Roots	Emetics and snuffed or inhaled medicines; Ground roots are taken as snuff for headaches; Dried, powdered roots taken as snuff or decoctions taken orally for headache; Dried, powdered roots taken as snuff	Hutchings, (1989); Hutchings et al. (1996); Moffett, (2016); Van Wyk et al. (1997)
	*Artemisia afra* Jacq. ex Willd. [*A. tenuifolia* Moench]	African wormwood (E); Wilde-als (A); Umhlonyane (X); Mhlonyane (Z); Lengana (B)	Shrub	Leaves	Leaves used in the treatment of headache and anxiety; Infusions or steam from crushed leaves are commonly inhaled for headaches and colds; Unspecified; Tea made from leaves used to treat headache; Unspecified	Hulley and Van, Wyk (2019); Hutchings et al. (1996); Stafford, (2009); Thring and Weitz, (2006); Van Wyk et al. (1997)
	*Artemisia dracunculus* L. [*A. dracunculoides* Pursh]	True tarragon, biting dragon (E)	Herb	Unspecified	Unspecified	Stafford, (2009)
	*Pluchea scabrida* DC. [*Conyza scabrida* (DC.) DC. Ex Miq]	Oven bush (E); Bakbos (A); Mokotedi-wa-thaba (S)	Shrub	Leaves	Unspecified parts used to treat headache; In Transkei, ground leaves are snuffed for headaches; Roots are used to treat depression; Leaves are placed on cloth with vinegar/brandy and wrapped around head for headache; Unspecified; Leaves placed in cloth with vinegar/brandy and wrapped around the head to treat headache	Hulley and Van Wyk, (2019); Hutchings et al. (1996); Mogale et al. (2019); Moteetee et al. (2019); Thring and Weitz, (2006); van Wyk et al. (1997); Van Wyk et al. (2008)
	*Tarchonanthus camphoratus* L. *[T. camphoratus* var. *camphoratus, T. abyssinicus* Sch.Bip.]	Camphor bush (E); Kankerbos (A); Igqeba-elimhlophe (Z); Sefahla (S)	Tree	Branches; leaves	Sotho’s use smoke from burning green branches as an inhalant for headaches; Infusions of leaves and twigs used to treat headache; Branches are burnt, and smoke inhaled for the relief of headache	Hutchings et al. (1996); Moffett, (2016); Venter and Venter, (2016)
Cannabaceae	*Cannabis sativa* L	Marijuana (E); Dagga (A); Umnya (X); Matekwane (S); Nsangu (Z)	Herb	whole plant	Used in the treatment of depressive mental conditions; Whole plant is used to treat “Vaal sick” and excessive headache; Smoked to induce well-being, relaxation, sociability and/or spirituality; Administered orally, intravenously or by topical application for treatment of depression and other conditions	Hutchings et al. (1996); Mongalo et al. (2018); Van Wyk and Gericke, (2000); Van Wyk et al. (1997)
Capparaceae	*Capparis tomentosa* Lam. [*C. alexandrae* Chiov., *C. biloba* Hutch. and Dalziel, *C. floribunda* Wight]	Woolly caper bush (E); Wollerige(A); Imfihlo (X); Umabusane (Z)	Shrub	Roots	Emetics and snuffed or inhaled medicines; Roots are burnt to form a powder that is rubbed into scarifications for the relief of headache; The Zulu use unspecified parts to treat madness; Powdered, burnt roots rubbed into skin for headache	Hutchings, (1989); Hutchings et al. (1996); Sobiecki, (2002); Van Wyk et al. (1997)
	*Maerua angolensis* DC. [*M. angolensis* subsp. *angolensis*]	Bead-bean tree, bead-pod tree (E); Knoppiesboontjieboom (A); Umenwayo (Z); Mogogwane (S); Mutamba-na-mme (V)	Tree	Leaves	Steam from leaves inhaled to treat headache	Venter and Venter, (2016)
Euphorbiaceae	*Synadenium cupulare* L.C. Wheeler	Dead-man’s tree (E); Gifboom (A); Umbulele (Z)	Tree	Leaves	Emetics and snuffed or inhaled medicines; Leaves are broken up and inhaled to relieve headaches; Leaves are used as medicine for headache	Hutchings, (1989); Hutchings et al. (1996); Van Wyk and Gericke, (2000)
Fabaceae	*Albizia adianthifolia* (Schum.) W.Wight [*A. adianthifolia* var*. adianthifolia* Schum.) W.Wight, *Mimosa adianthifolia* Schum.]	Flat- crown albizia (E); Platkroon (A); Umgadankawu (Z); Umhlandlothi (X)	Tree	Bark	Taken as snuff; Powdered bark is taken as a snuff for headaches; Powdered bark used as snuff; Bark is powdered and used as snuff for the relief of headache	Corrigan et al. (2011); Hutchings et al. (1996); Van Wyk et al. (1997); Venter and Venter, (2016)
	*Tephrosia capensis* (Jacq.) Pers	Cape Tephrosia (E); Pelodimaroba (S)	Shrub	Roots	Emetics and snuffed or inhaled medicines; Dried powdered roots are used as snuff to relieve headaches; Dried roots snuffed for headache; Dried powdered roots are used as a snuff for headaches and plant decoctions for nervousness	Hutchings, (1989); Hutchings et al. (1996); Moffett, (2016); Sobiecki, (2002)
Hypericaceae	*Hypericum perforatum* L. [*H. vulgare* Lam., *H. perforatum* var. *petiolatum* Peterm	Saint John’s wort (E); Johanneskruid (A)	Shrub	Whole plant	Popular in the West and in South Africa for treating mild depression, anxiety and sleep disorders; Powdered extracts used as antidepressants	Sobiecki, (2002); Van Wyk et al. (1997)
	*Hypericum revolutum* Vahl [*H. kalmianum* Vahl, *H. revolutum* subsp. *revolutum*]	Curry bush, forest primrose (E); Kerriebos (A)	Shrub	Unspecified	Unspecified	Stafford, (2009)
Hypoxidaceae	*Hypoxis hemerocallidea* Fisch., C.A. Mey. and Avé-Lall. [*H. elata* Hook. f., *H. obconica* Nel, *H. patula* Nel, *H. rooperi* T. Moore, *H. rooperi var. forbesii* Baker	Star flower, yellow star (E); Sterblom (A); Inkomfe (Z); Lotsane (S)	Shrub	Corm	Emetics and snuffed or inhaled medicines; Corm infusions are given as emetics for mental disorders; Used as charm to cure headache and for anxiety and depression; Medicinal plant used for headache; Corm infusions are used for insanity; Infusions of corms and leaves used as emetics	Hutchings, (1989); Hutchings et al. (1996); Moffett, (2016); Moteetee et al. (2019); Sobiecki, (2002); Van Wyk et al. (1997)
Lamiaceae	*Ballota hirsuta* Benth. [*B. africana* Colmeiro, *B. cinerea* (Desr.) Briq.]	Cape horehound (E); Katterkruie (A)	Shrub	Leaves	Leaf infusions used for treating headache; Compresses on head to treat headache; Treats headaches; Used to treat headaches; Infusions used to treat headache; Drank to treat headache	Hulley and Van Wyk (2019); Nortje and van Wyk (2015); Philander (2011); Thring and Weitz (2006); Van Wyk et al. (1997); Van Wyk et al. (2008)
	*Leonotis leonurus* (L.) R.Br. [*Leonurus africanus* Mill., *Leonurus grandiflorus* Moench, *Leonurus superbus* Medik., *Phlomis leonurus* L., *P. speciosa* Salisb.]	Lion’s ear, wild dagga (E); Wildedagga (A); Imvovo (X); Umcwili (Z)	Shrub	Whole plant	Emetics and snuffed or inhaled medicines; Cold water infusions from leaves are inhaled to relieve feverish headaches; Unspecified; Leaves are smoked for epilepsy and partial paralysis; Unspecified parts used for headache; Decoctions of flowers, stems and leaves are used to treat headache; Decoctions taken for headache	Hutchings, (1989); Hutchings et al. (1996); Philander, (2011); Sobiecki, (2002); Stafford, (2009); Thring and Weitz, (2006); Van Wyk and Gericke, (2000); Van Wyk et al. (1997)
	*Mentha longifolia* (L.) L. [*M. longifolia* (L.) Huds.]	Wild mint (E); Kruisement (A); Bohatsu (S); Umfuthana lomhlhanga (Z)	Herb	Leaves	Leaf infusion drank as tea and warm compress of leaves used for headache; Unspecified part compressed on the head for headache; Emetics and snuffed or inhaled medicines; Sotho’s sometimes plug their nose with crushed leaves and bind with a cloth for the relief of headaches; Used medicinally to treat headache; Unspecified parts used to treat headache; Crushed leaf infusions or decoctions drank for headache; Leaf infusion mixed with *kruisement* in tea for headache and general malaise	De Beer and Van Wyk, (2011); Hulley and Van Wyk, (2019); Hutchings, (1989); Hutchings et al. (1996); Philander, (2011); Thring and Weitz, (2006); Van Wyk et al. (1997); Van Wyk et al. (2008)
	*Mentha spicata* L. [*Mentha crispata* Schrad. ex Willd.]	Spearmint, garden mint (E)	Herb	Leaves	Leaf infusion taken as tea to treat headache and colds	Van Wyk et al. (2008)
	*Rosmarinus officinalis* L. [*R. communis* Noronha, *R. communis* var. *communis*]	Rosemary (E)	Shrub	Unspecified	Used medicinally to treat headache	Philander, (2011)
Lauraceae	*Cinnamomum camphora* (L.) J.Presl [*Camphora* (L.) H.Karst., *Laurus camphora* L.]	Camphor laurel, camphor tree (E)	Tree	Unspecified	Details not disclosed	Stafford, (2009)
	*Ocotea bullata* (Burch.) E. Meyer in Drege [*Laurus bullata* Burch., *Oreodaphne bullata* (Burch.) Nees]	Black stinkwood (E); Stinkhout (A); Unukani (X,Z)	Tree	Bark	Emetics and snuffed or inhaled medicines; Bark used as snuff, inhaled to treat headache; South Africans use unspecified parts as an emetic for emotional and nervous disorders; Finely ground bark used as snuff for headache	Hutchings, (1989); Hutchings et al. (1996); Sobiecki, (2002); Van Wyk et al. (1997)
Meliaceae	*Ekebergia capensis* Sparrm. [*E. mildbraedii* Harms*, E. ruppeliana* (Fresen.) A. Rich., *E. senegalensis* Fuss]	Cape ash (E); Essenhout (A); Mmidibidi (S); Umnyamatsi (SS)	Tree	Leaves and roots	Vha-Venda use leaves and bark in emetics and for headache; Leaves are pounded in cold water and the solution is extracted and inhaled to treat mental problems; Roots used to treat headache; Root decoction taken orally to relieve headache	Hutchings et al. (1996); Sobiecki, (2002); Van Wyk et al. (1997); Venter and Vente, (2016)
	*Melia azedarach* L	Chinaberry, persian lilac (E)	Tree	Leaves	Infusions made from a handful of leaves in half a cup of water are taken for abdominal pains	Hutchings et al. (1996)
Myricaceae	*Morella serrata* (Lam.) Killick [*Myrica serrata* Lam*.*]	Mountain Waxberry (E); Berg-wasbessie (A); Ulethu (Z); Umaluleka (X); Maleleka (S)	Shrub	Rootbark	Emetics and snuffed or inhaled medicines; Rootbark decoctions are taken for headaches; Rootbark used for headache	Hutchings, (1989); Hutchings et al. (1996); Moffett, (2016)
Oleaceae	*Olea europaea* subsp*. cuspidata* (Wall. and G.Don) Cif. [*O. europaea* subsp. a*fricana* (Mill.) P.S.Green Kew Bull., *O. chrysophylla* Lam., *O. kilimandscharica* Knobl.]	Olive tree, wild olive (E); Olienhout (A); Umnquma (Z, X); Motlhware (B); Mutlhwari (V)	Tree	Leaves	Unspecified; Infusions of dry leaves used to treat headache; Medicinal plant used for headache; Unspecified	Hutchings et al. (1996); Moffett, (2016); Moteetee et al. (2019); Stafford, (2009)
Passifloraceae	*Adenia gummifera* (Harv.) Harms [*Modecca gummifera* Harv., *A. rhodesica* Suess., *A. gummifera* var*. gummifera*]	Snake-climber, monkey rope (E); Slangklimop (A); Impinda (Z)	Shrub	Roots	Root is used to make tonic, taken orally as stimulant for seediness or depression; Infusions made from roots in boiling water are administered as emetic tonics or stimulants for seediness or depression; Unspecified parts used to treat depression	Corrigan et al. (2011); Philander, (2011); Sobiecki, (2002)
Peraceae	*Clutia pulchella* L. [*C. cotinifolia* Salisb*., C. pulchella* var. *genuina* Müll.Arg., *C. pulchella* var. *pulchella*, *C. gapinii* Pax]	Common lightning bush (E); Gewone bliksembos (A); Podimolwetse (S); Umsimpane (X); Umembesa (Z)	Shrub	Unspecified	Used as emetics and snuffed or inhaled medicines; Used to treat headaches; Medicinal plant used for headache	Hutchings, (1989); Moffett, (2016); Moteetee et al. (2019)
Phyllanthaceae	*Pseudophyllanthus ovalis* (E.Mey. ex Sond.) Voronts and Petra Hoffm. [*Andrachne ovalis* (E.Mey. ex Sond.) Müll.Arg., *Savia ovalis* (E.Mey. ex Sond.) Pax and K.Hoffm.]	False lightning bush (E)	Shrub	Roots	Used as emetics and snuffed or inhaled medicines; Burnt roots are sniffed for headache; Root emetics taken to relieve morning stress and body aches and burned roots snuffed for headache	Hutchings, (1989); Hutchings et al. (1996); Philander, (2011)
Poaceae	*Cymbopogon nardus* (L.) Rendle [*C. virgatus* Stapf ex Bor, *C. validus* (Stapf) Stapf ex Burtt Davy, *Sorghum nardus* (L.) Kuntze]	Tamboekiegras (A); Isicunge/isiqunga (Z)	Grass	Shoot, roots	Used to revitalise the nerves of moody people, Zulu use the roots and shoots to strengthen the nervous system	Sobiecki, (2002)
Polygalaceae	*Securidaca longipedunculata* Fresen. [*Elsota longipendunculata* (Fresen.) Kuntze, *S. longipendunculata* var. *longipendunculata*]	Violet tree, fibre tree (E); Rooipeultjie (A); Mmaba (S); Iphuphuma (Z); Mpesu (V)	Tree	Roots; wood	Root kernel is used to treat headache; Powdered root/wood rubbed on forehead for headache	Mongalo et al. (2018); Van Wyk et al. (1997)
Polygonaceae	*Rumex sagittatus* Thunb. [*R. scandens* Burch., *Acetosa sagittata* Johnson and Briggs]	Climbing dock (E); Ranksuring (A); Umdende (Z); Tshitamba-tshedzi (V); Bodilaboboholo (S)	Herb	Rootstock	Emetics and snuffed or inhaled medicines; Powdered rootstock used by the Sotho as a snuff for headaches; Powdered rootstock used as snuff for headache; Used medicinally to treat headache	Hutchings, (1989); Hutchings et al. (1996); Moffett, (2016); Moteetee et al. (2019)
Pteridaceae	*Adiantum capillus*-*veneris* L. [*A. capillus*-*veneris* var*. capillus*-*veneris* L., *A. capillus*-*veneris* f. *dissectum* (M. Martens and Galeotti) Ching]	Southern maidenhair fern (E)	Herb	Leaves	Used as emetics and snuffed or inhaled medicines; Dried leaves are smoked for head and chest colds	Hutchings, (1989); Hutchings et al. (1996)
Ranunculaceae	*Ranunculus multifidus* Forssk. [*R. striatus* Hochst. ex A. Rich., *R. udus Freyn*.]	Common buttercup (E); Botterblom, kankerblare (A); Isijojokazana (Z); Hlapi (S)	Herb	Unspecified	Emetics and snuffed or inhaled medicines; Burning plant inhaled by the Sotho people to relieve headache; Smoke is inhaled to relieve headache	Hutchings, (1989); Hutchings et al. (1996); Moffett, (2016)
Rhamnaceae	*Ziziphus mucronata* Willd. [*Z. madecassus* H. Pierrier*, Z. mucronata* subsp. *mucronata*]	Buffalo thorn (E); Blinkblaar-wag-ŉ-bietjie (A); Umphafa (Z); Mongalo (S)	Tree	Leaves; bark	Unspecified; Powdered leaf and bark in water is taken as an emetic	Van Wyk et al. (1997); Venter and Venter, (2016)
Rutaceae	*Ptaeroxylon obliquum* (Thunb.) Radlk. [*P. utile* Eckl. and Zeyh., *Rhus obliqua* Thunb.]	Sneezewood tree (E); Nieshout (A); Umthathi (X)	Tree	Bark and wood	Emetics and snuffed or inhaled medicines; Xhosas use powdered bark traditionally as a snuff and medically to relieve headaches; Used medicinally to treat headache; Powdered bark used as snuff; Powdered wood used as snuff; Bark and wood used to make snuff to treat headache	Hutchings, (1989); Hutchings et al. (1996); Philander, (2011); Sobiecki, (2002); Van Wyk et al. (1997); Venter and Venter, (2016)
Salicaceae	*Salix mucronata* Thunb. [*S. subserrata* Willd.]	Cape Willow (E); Kaapse Wilger (A); Mogokare (S); Umnyezane (Z); Munengeledzi (V)	Tree	Leaves, roots	Leaves are compressed on the head to treat headache; Leaf compress used for headache; Used medicinally to treat headache; Decoctions or infusions used for headache; Root decoction used to treat headache	Hulley and Van, Wyk (2019); Nortje and van, Wyk (2015); Philander, (2011); Van Wyk et al. (1997); Venter and Venter, (2016)
Solanaceae	*Datura ferox* L. [*D. laevis* Bertol. *D*. *quercifolia* Kunth]	Long-spined thorn apple (E); Groot stinkblaar (A)	Shrub	Unspecified	Unspecified	Stafford, (2009)
	*Datura metel* L. [*D. metel* var. *dentata* Schltdl. and Cham., *D. metel* var. *fastuosa* (L.) Saff.]	Angel’s trumpet (E)	Shrub	Unspecified	Emetics and snuffed or inhaled medicines; Unspecified parts smoked for the relief of headache; Unspecified	Hutchings, (1989); Hutchings et al. (1996); Sobiecki, (2002)
	*Datura stramonium* L. [*D. stramonium* var. *canescens* Roxb., *D. stramonium* var. *chalybaea* W.D.J.Koch]	Common thorn apple (E); Malpitte (A); Ijoyi, umhlabavutha (X); Iloyi (Z)	Shrub	Leaves	Unspecified part compressed on the head to relieve headache; Emetics and snuffed or inhaled medicines; Unspecified parts smoked for the relief of headache; The Venda use the leaves to treat insanity. Healers inhale powdered roots and leaves as snuff for divinatory purposes; Unspecified; Dried and powdered leaves used as consciousness-altering snuff by diviners; Leaves used to treat headache	Hulley and Van Wyk, (2019); Hutchings, (1989); Sobiecki, (2002); Stafford, (2009); Thring and Weitz, (2006); Van Wyk and Gerickev (2000); Van Wyk et al. (1997)
	*Nicotiana glauca* Graham [*N. glauca lateritia* Lillo]	Tree tobacco (E)	Shrub	Leaves	Compressed on the head for headache (external use only); Leaf compress used to treat headache; Leaves are warmed and put on the head to relieve headache; Fresh leaves applied to the head as a poultice for headache	Hulley and Van Wyk (2019); Nortje and van Wyk (2015); Van Wyk and Gericke (2000); Van Wyk et al. (2008)

Species and family names for each plant species were validated in reference to The Plant List (www.theplantlist.org), The World Flora Online (http://theworlflora.online) and PlantZAfrica (http://pza.sanbi.org/) and the local names were confirmed using PlantZAfrica (http://pza.sanbi.org/). ^#^Local name: A, Afrikaans; E, English; S, Sotho; SS, Southern Sotho; V, Venda; X, Xhosa; Z, Zulu.

*Boophone disticha* is an important Southern Africa medicinal bulb and is popular in South Africa where it is used as herbal medicine by traditional healers to induce hallucinations, and as a medication for mental disorders (Hutchings et al., 1996; Stafford et al., 2008; Neergaard et al., 2009). Bulb infusions of this plant are drank by South African traditional healers and patients (Neergaard et al., 2009), and weak decoctions made from the bulb scales of are taken by mouth or as enemas for headache (Hutchings et al., 1996; Van Wyk et al., 1997). *Sceletium tortuosum* has recently attracted attention for its long history of use in traditional medicine and its possible use in promoting well-being and treating depression and/or stress (Sobiecki, 2002; Harvey et al., 2011). It was likely to have been used in the prehistoric times by hunter-gatherers and pastoralists as a mood-altering substance (Van Wyk and Gericke, 2000). The whole plant is chewed or drank traditionally as a psychoactive substance or medicine to elevate mood, reduce stress and treat anxiety and depression disorders (Hutchings et al., 1996; Van Wyk et al., 1997; Sobiecki, 2002). *Xysmalobium undulatum* has a long history of therapeutic use in South African traditional medicine (Helmstädter, 2015). The roots of this plant contain glycosides with weak central nervous system depressant and the extracts have demonstrated antidepressant activity (Hutchings et al., 1996). Powdered roots are used as snuff or inhaled medicine and as emetics (Hutchings, 1989; Van Wyk et al., 1997). Unspecified parts of *Agapanthus campanulatus* are used by the Sotho to treat people with “spirit,” which is a type of mental disturbance (Sobiecki, 2002). Infusions made from leaves, fruits, and leaf decoctions of *Schinus molle* are used traditionally as antidepressants (Bhat and Jacobs, 1995). Fresh leaves are placed on a cloth with vinegar and wrapped as compress on the head to treat headache (Van Wyk et al., 2008; Nortje and van Wyk, 2015; Hulley and Van Wyk, 2019). *Cannabis sativa* is widely used in traditional African medicine for both recreational and medicinal purposes (Hutchings et al., 1996; El-Alfy et al., 2010). The whole plant is used to treat “Vaal sick” and intense headache (Mongalo et al., 2018), smoked to induce well-being, relaxation, sociability and/or spirituality and administered orally, intravenously or by topical application for treatment of depression and other related conditions (Van Wyk et al., 1997; Van Wyk and Gericke, 2000). Decoctions from the leaves of *Heteromorpha trifoliata* (H.L.Wendl.) Eckl. and Zeyh. are administered for mental and nervous diseases and smoked for headaches (Hutchings et al., 1996). The Sotho and Xhosa administer leaf decoctions and infusions for the same purpose (Hutchings et al., 1996; Sobiecki, 2002). The continuous reliance on traditional medicine has led to an extensive indigenous knowledge and expertise within local communities, and documentation thereof, from which herbal product development can be initiated (Fennell et al., 2004; Ndhlala et al., 2011).

### Plant Parts Used, Methods of Preparation and Routes of Administration

Based on the reviewed ethnobotanical literature, different plant parts were used in the preparation of medicinal plants used to treat MDD and MDD-com. Plant parts used for the preparations are specified in most studies, with leaves (49 times) being the most predominantly used plant part (Figure 2). However, about 35% (66) of the plants did not specify the plant parts used in the preparation of plant medicines. Fourteen plant parts (e.g. leaves, bark, roots, seeds, bulbs and the stem are used for the preparation of herbal medicines in various studies (Figure 2). Recorded plants were prepared using various preparation methods, including decoction, infusion, burning, compressing, crushing, pounding, tea and powder. The most frequently used method of preparation was burning, which accounted for approximately 21% of the plant medicines recorded (Figure 3). This method involves burning plant material and inhaling the smoke or smoking the dried plant material. The second most frequently used methods of preparation are infusion and powder, both used 28 times each. Recorded routes of administration included oral (drinking, chewing), nasal (snuffing, inhaling, steaming) and topical (applied on the skin, wrapped around the head). The most dominant route of administration is the nasal route which includes plants that are powdered and sniffed, and plants that are burnt and their smoke inhaled.

### Plant Families Used for Depression and Related Ailments

**FIGURE 4:**
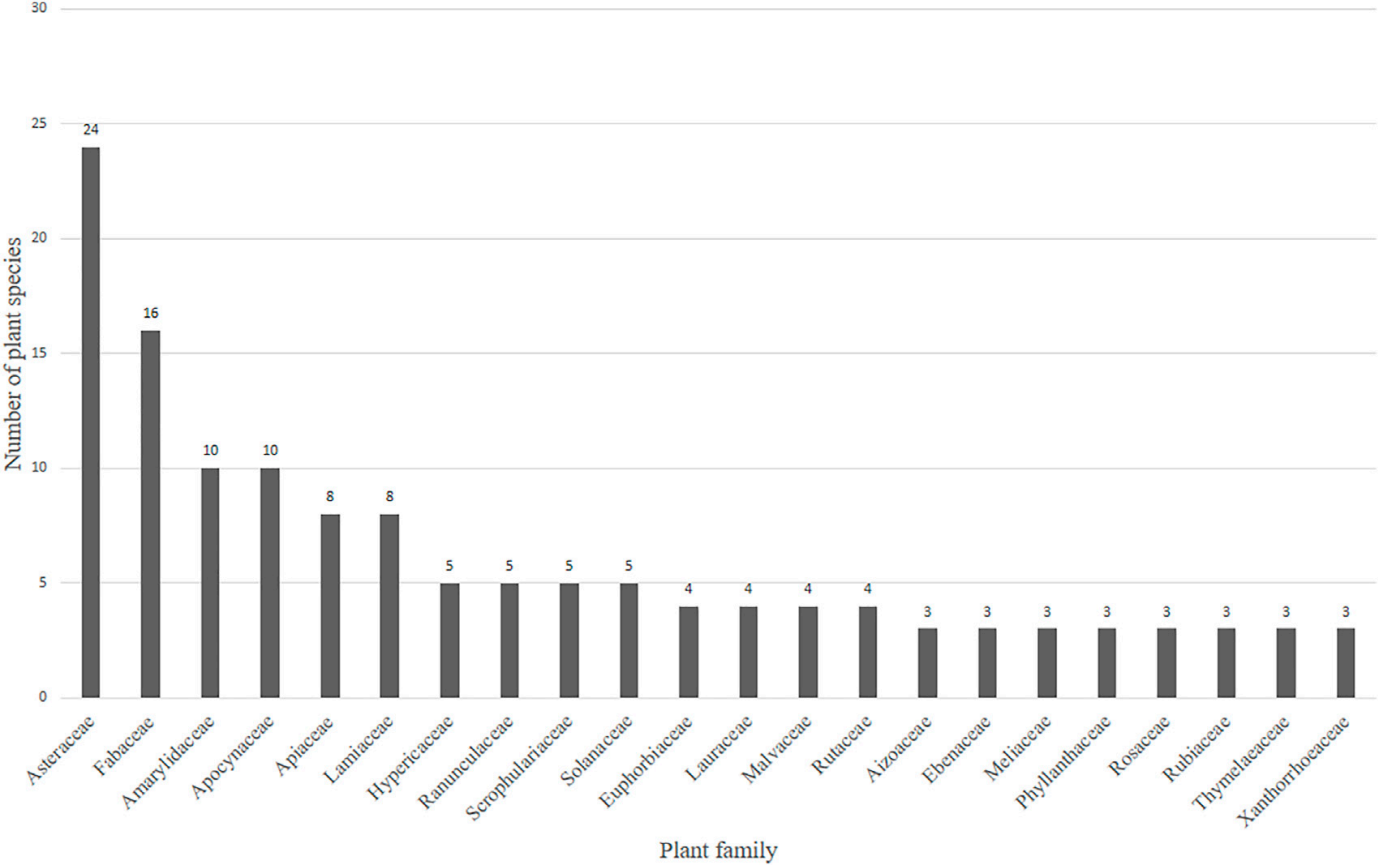
Twenty-two plant families with three or more plant species recorded for the treatment of depression. In total, 63 plant families representing 186 plants were recorded in Supplementary Table S1. The remaining 41 families had only one or two plants. Thirty plant families were recorded in Table 2 as representing a narrowed-down 54 popular medicinal plants.

A total of 63 plant families are being used in South Africa to treat MDD and MDD-com (Figure 4 and Supplementary Table S1). Twenty-two of the plant families recorded represented more than two plant species used traditionally against MDD. The remaining 41 plant families represented only one or two plant species. Plant families with three or more plant species recorded for the treatment of depression and related ailments are presented in Figure 4. Several plant families contain relatively higher numbers of plant species with potential antidepressant effects than others. Families with the highest numbers of plant species included Asteraceae (24) and Fabaceae (16), Amaryllidaceae (10), and Apocynaceae (10).

Asteraceae (Compositae) as a plant family is known to have diverse therapeutic applications, and has a long history in traditional medicine (Rolnik et al., 2021). Members of the Asteraceae are commonly used to treat various diseases since ancient times (Panda et al., 2018), including the observation in the current review (Figure 4). Asteraceae are known to produce a large quantity of terpenoids, such as hemiterpenes, sesquiterpenes, diterpenes, monoterpene and polyterpenes, and flavonoids, with common flavonols, quercetin and kaempferol and flavones apigenin and luteolin being widely distributed (Hutchings et al., 1996; Sülsen et al., 2017). Trigo et al. (2003) isolated and identified pyrrolizidine alkaloids of the senecionine, platyphylline, rosmarinine, senkirkine subgroups and triangularine group from the inflorescences of 14 *Senecio* (Asteraceae), one of the largest genera of flowering plants distributed worldwide. Sarker et al. (2001) investigated a methanolic extract from the seeds of *Centaurea cyanus* L. (Asteraceae) using preparative RP-HPLC analysis and afforded four alkaloids of the indole variety: moschamine, *cis*-moschamine, centcyamine and *cis*-centcyamine. Existing pharmacological studies have reported the antidepressant-like activity of *Artemisia dracunculus* L. (Asteraceae) in animal models of depression (Jahani et al., 2019; Ilkhanizadeh et al., 2021)*.* Although these studies did not isolate or identify any phytochemicals in their experiments, several phenolic compounds, such as, syringic acid, vanillic acid, chlorogenic acid, ferulic acid, caffeic acid, quercetin and luteolin are present in the plant material of *A. dracunculus (* Mumivand et al., 2017). The presence of these alkaloids may be rationale behind the extensive use of plants in this family for the alleviation of depressive symptoms in traditional medicine. Despite this plant family being the most mentioned in ethnobotany surveys, only a few of the plants have been investigated pharmacologically using models of depression to validate the recorded traditional uses.

Fabaceae often produces indole variety alkaloids, such as N-methyltryptamine, N-methyltryptophan, and choline, able to mimic the structure of the neurotransmitter serotonin, thereby antagonizing its action with resulting neuroprotective effects (Hutchings et al., 1996). Although no specific phytochemicals were isolated, aqueous extract of *Albizia adianthifolia* (Fabaceae) leaves increased swimming time and decreased immobility time in the forced swimming test conducted using male Wistar rats (Beppe et al., 2015). The antidepressant-like effect of this plant may be attributed to the potential presence of the indole alkaloids (Hutchings et al., 1996). The similarity in structure of indole alkaloids to neurotransmitters including serotonin has led to the prediction of the potential neurological and antidepressant effects of several medicinal plants and their active phytochemicals (Hamid et al., 2017). Further investigations are required to identify these alkaloids from medicinal plants belonging to the Fabaceae and explore their antidepressant-like effects.

Amaryllidaceae are extensively used traditionally for CNS activation, with uses such as treatment depression, epilepsy and other mental disorders (Stafford et al., 2008; Nair and Van Staden, 2014). Their pharmacological efficacy can be attributed to the presence of unique alkaloids previously isolated from several medicinal plants belonging to this plant family (Nair et al., 2013; Elgorashi, 2019). Amaryllidaceae is one of the 20 most important alkaloid containing plant families (Koutová et al., 2020). Plants from the Amaryllidaceae produce isoquinoline alkaloids classified into unique structurally diverse groups, with three major structural-types galanthamine, lycorine and crinine (Viladomat et al., 1997; Nair and Van Staden, 2013). The minor series of these alkaloids include tazettine, homolycorine, and montanine (Nair et al., 2013; Nair and Van Staden, 2013). Previous studies of *Boophone disticha* (Amaryllidaceae), a popular plant in South African traditional medicine, led to the identification of buphanamine, buphanisine, buphanidrine, distichamine and crinine and a confirmation of its antidepressant effects (Nielsen et al., 2004; Sandager et al., 2005; Neergaard et al., 2009). Raghoo et al. (2021) identified aromatic, lycorine or crinine type Amaryllidaceae alkaloids from the bulb of *Ammocharis coranica* (Koorbanally et al., 2000a; Elisha et al., 2013). Bay-Smidt et al. (2011) explored the tribe Haemantheae for potential target species for the discovery of serotonin reuptake transport protein inhibitors. From the study, lycorine, homolycorine and montanine type alkaloids were isolated from *Haemanthus hirsutus* Baker, the extract of *H. sanguineus* Jacq. yielded only montanine type alkaloids and *H. coccineus* L. yielded montanine type and crinine type alkaloids, all with antidepressant effects. Furthermore, the alkaloid-rich extracts *Haemanthus coccineus*, *H. montanus* Baker and *H. sanguineus* yielded two isoquinoline montanine type Amaryllidaceae alkaloids, montanine and coccinine, both with antidepressant effects (Stafford et al., 2013). This serves as evidence that the plant family Amaryllidaceae is of significance and importance in phytochemical-based antidepressant drug discovery.

Apocynaceae as a plant family is an important and popular source of a number of drugs including simple indoles, carbolines, steroidal amines, isomeric quinindolines and quinindoles and miscellaneous type alkaloids (Raffauf and Flagler, 1960; Dey et al., 2017). Members of the Apocynaceae contain alkaloid ibogaine, which is used as a psychedelic drug for the treatment of substance addiction (Koenig and Hilber, 2015; Dey et al., 2017), ajmalicine, an alkaloid used as an antihypertensive drug used to treat high blood pressure (Wink and Roberts, 1998), and alstonine, an antipsychotic picralima alkaloid which prevents hyperlocomotion, memory deficit and social interaction deficit through antipsychosis mediated by 5-HT2A/C receptors (Dey et al., 2017). Members of the Apocynaceae often produce a vast range of indolic alkaloids, including tryptophan, and harman type alkaloids that are psychoactive (Trease and Evans, 1983). *Mondia whitei* (Hook.f.) Skeels commonly known as White’s ginger, is a popular South African plant used in folk medicine to treat diseases of the nervous system and it has demonstrated antidepressant properties under *in vitro* conditions (Pedersen et al., 2006; Pedersen et al., 2008; Neergaard et al., 2010; Aremu et al., 2011; Grabarczyk et al., 2015). Koorbanally et al. (2000b) reported the isolation and identification of the previously identified chemical compounds 2-hydroxy-4-methoxybenzaldehyde andreported the presence of Isovanillin from a methylene chloride extract of *M. whitei*. Neergaard et al. (2010) isolated a monoterpene lactone (−)-loliolide from *M. whitei* leaves and tested it for *in-vitro* for its affinity to SERT. The extracts showed good displacement of [3H]-citalopram in the SERT binding assay. In a binding assay, extracts of *M. whitei* had exhibited affinity to SERT (Nielsen et al., 2004). Ethanolic extracts have further exhibited antidepressant activity in a functional SERT inhibition assay, and had an antidepressant-like effect in two *in vivo* models of depression (Pedersen et al., 2008). The authors suggested that the *in vitro* serotonin transporter affinity exhibited by the plant extracts was due to the lactone (−)-loliolide.

Two members of the Solanaceae have demonstrated antidepressant-like effects in *in vitro* and *in vivo* investigations that evaluated the potential antidepressant effects of medicinal plants. An aqueous extract from *Datura ferox* L. seeds exhibited high affinity the SERT binding assay (Nielsen et al., 2004), while extracts from the fresh leaves of *Datura stramonium* L. exhibited antidepressant-like effects in the forced swimming test and open field test conducted by Devi et al. (2012). Other families representing medicinal plant species with pharmacological evidence against MDD are Aizoaceae (*Sceletium tortuosum* (L.) N.E. Br.), Cannabaceae (*Cannabis sativa* L.), Hypericaceae (*Hypericum perforatum* L.), Pteridaceae (*Adiantum capillus-veneris*), Lauraceae (*Cinnamomum camphora*), Poaceae (*Cymbopogon nardus*), Capparaceae (*Maerua angolensis*), Meliaceae (*Melia azedarach*), Polygalaceae (*Securidaca longipedunculata*), Rhamnaceae (*Ziziphus mucronata*) and Anarcadiaceae (*Schinus molle*) (Figure 4).

To allow for easy identification and selection of potential medicinal plants for pharmacological investigation, the relationship between plant families with potential antidepressant effects and the various phytochemicals present in plants belonging to these families remain pertinent. This was achieved by looking closely at the phytochemical profiles of medicinal plants belonging to the four plant families with the highest number of plants recorded in this review. Based on the fact that plants in the same family may have similar phytochemical profiles, it is hypothesized that recognizing plant families of potential (based on the presence of anti-depressive phytochemicals) could lead to the discovery of potential medicinal plants within those families for pharmacological investigations using models of MDD. This approach may provide insight on the potential presence of novel phytochemical compounds of antidepressant value not previously isolated from medicinal plants and stimulate alternative antidepressant drug discovery.

### Phytochemicals With Potential Antidepressant Effects

The reported ethnobotanical uses of the 186 medicinal plants were based on indigenous knowledge and expertise from traditional healers and knowledgeable community members. Many of these plants contain phytochemicals with psychoactive and pharmacological effects ranging from sedation, stimulation to euphoria and hallucinations (Sobiecki, 2002). Moreover, their effects lead to altering of perception, emotion and cognition, and change in consciousness (Alrashedy and Molina, 2016; Khan et al., 2018; Martins and Brijesh, 2018). Only 27 (14.5%) of the recorded plants have been investigated pharmacologically for depression while only nine were phytochemically characterised to identify phytochemicals with antidepressant-like effects. A total of 24 phytochemicals with antidepressant-like effects have been isolated and identified (Table 3). We also identified the plant part from which the phytochemicals were isolated, the methods of extraction used, the methods of isolation and identification as well as the molecular structures of the compound**.** These plants were represented by 12 pharmacological studies previously conducted to investigate the *in vitro* and *in vivo* antidepressant-like effects. Medicinal plants with the highest number of phytochemicals identified are *Boophone disticha* and *Rosmarinus officinalis*, each with five phytochemicals isolated, identified and their antidepressant effects investigated in various models of depression (Sandager et al., 2005; Neergaard et al., 2009; Sasaki et al., 2013; Abdelhalim et al., 2015). Phytochemical studies on *Rosmarinus officinalis* led to the identification of carnosic acid, rosmarinic acid (Sasaki et al., 2013), salvigenin, rosmanol and cirsimaritin (Abdelhalim et al., 2015), with proven antidepressant effects in the FST (Machado et al., 2009; Abdelhalim et al., 2015), TST (Machado et al., 2009; Sasaki et al., 2013; Abdelhalim et al., 2015; Sasaki et al., 2021) and OFT (Machado et al., 2009). The mood elevating properties of extract *Sceletium. tortuosum*, Zembrin^™^, are due to the presence of mesembrine, a phytochemical with potent selective serotonin (5-HT) re-uptake activity (Van Wyk and Gericke, 2000; Harvey et al., 2011).

**TABLE 3:** Phytochemicals isolated and chemical structures of compounds from plant with antidepressant-like effects.

Scientific name	Reference	Plant part	Method of extraction	Methods for the isolation and identification	Compound	Molecular structure
*Boophone disticha*	Sandager et al. (2005)	Leaves	Vacuum filtration	Bioassay-guided fractionation on VLC and preparative TLC	Buphanamine	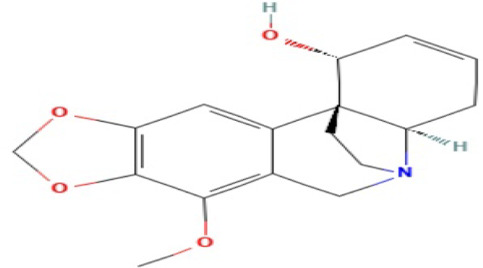
					Buphanadrine	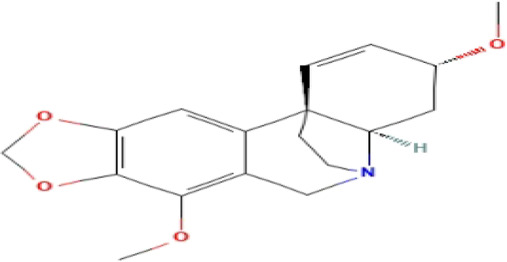
	Neergaard et al. (2009)	Bulbs	Liquid–liquid partitioning	HPLC–UV separation	Buphanamine	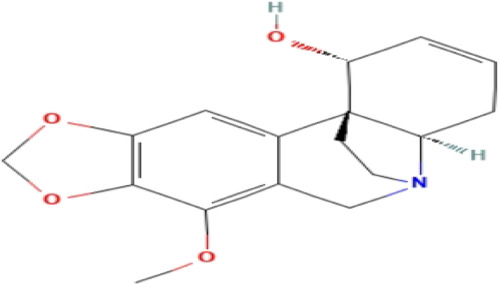
					Buphanisine	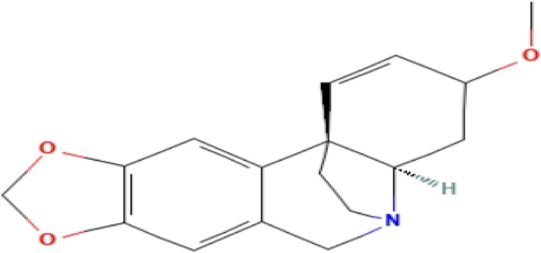
					Distichamine	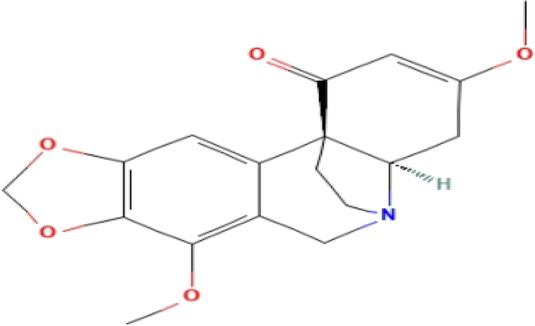
*Cannabis sativa*	El-Alfy et al. (2010)	Buds	Column chromatography	Preparative C18 HPLC	Δ^9^-tetrahydrocannabinol (Δ^9^-THC)	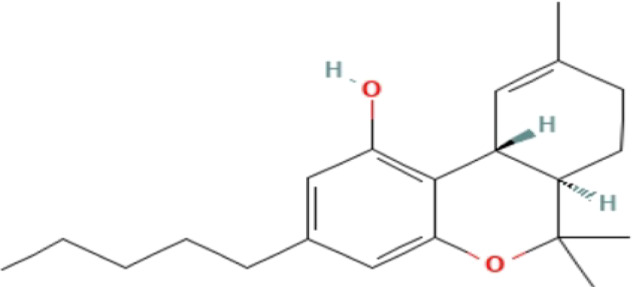
					Cannabidiol (CBD)	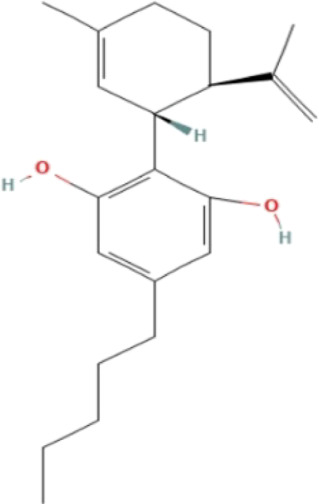
					Cannabichromene (CBC)	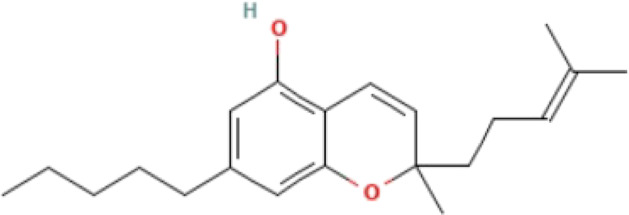
*Centella asiatica*	Kalshetty et al. (2012)	Leaves	Vacuum filtration	HPLC	Asiaticoside	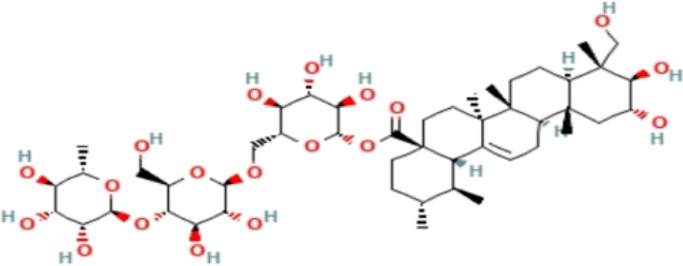
*Cymbopogon nardus*	Victoria et al. (2014)	Unspecified	Reduced pressure distillation	GC/MS	(R)-Citronellal	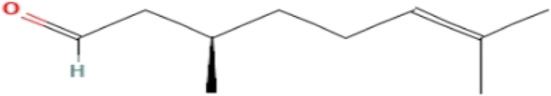
*Haemathus coccineus*	Stafford et al. (2013)	Bulb scales	Maceration	Column chromatography and TLC profile	Montanine	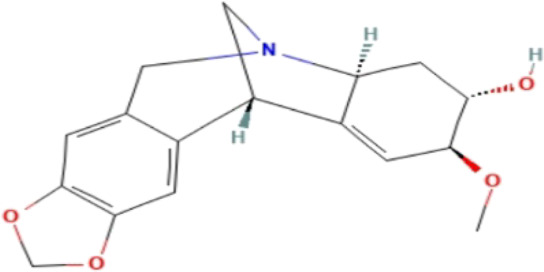
					Coccinine	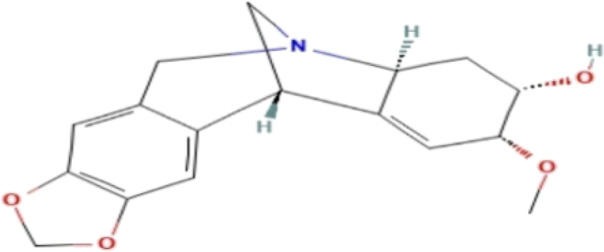
*Hypericum perforatum*	Tian et al. (2014)	Unspecified	Unspecified	Unspecified	Adhyperforin	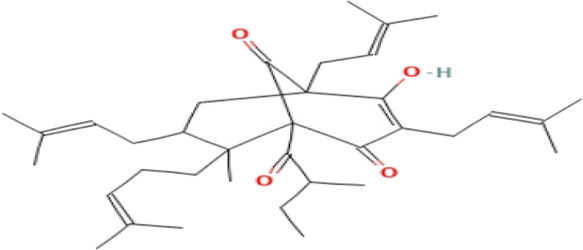
	Öztürk et al. (1996)	Aerial parts	Maceration	HPLC methods	Hypericin	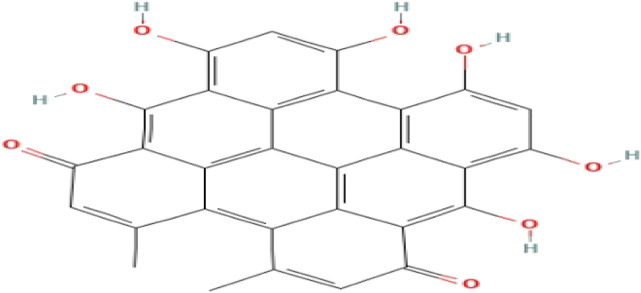
					Pseudohypericin	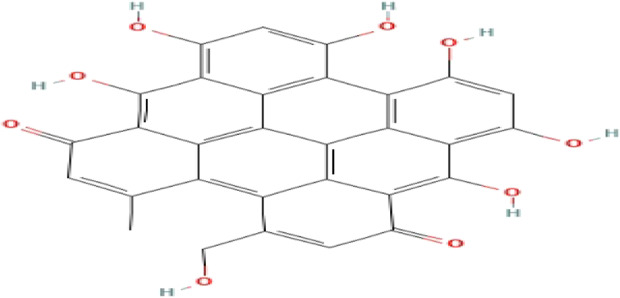
*Mondia whitei*	Neergaard et al. (2010)	Leaves	Liquid–liquid partitioning	Vacuum liquid chromatography and Preparative HPLC	Loliolide	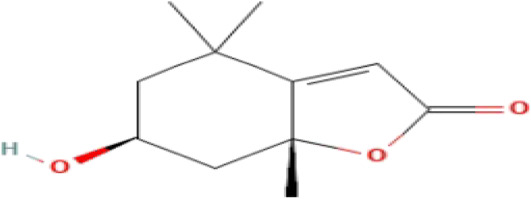
*Rosmarinus officinalis*	Sasaki et al. (2013)	Leaves	Maceration	HPLC analysis	Carnosic acid	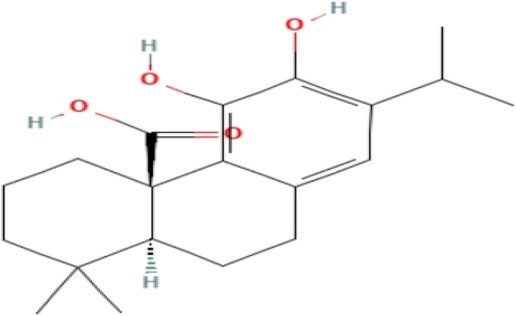
					Rosmarinic acid	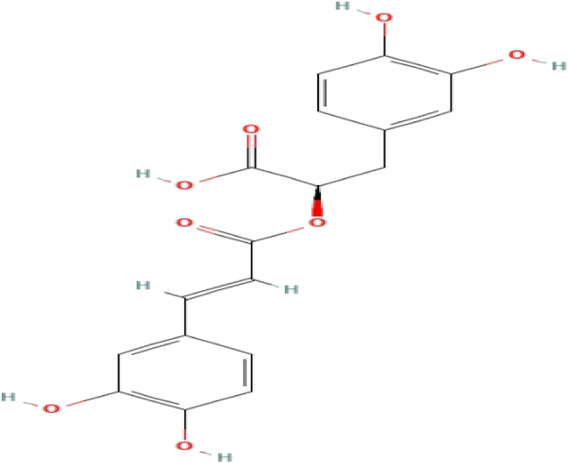
	Abdelhalim et al. (2015)	Whole plant	Infusion	Column chromatography and preparative thin layer chromatography	Salvigenin	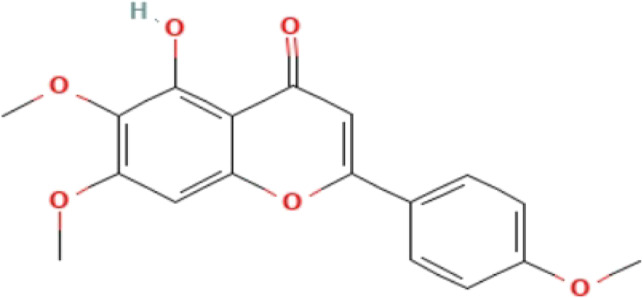
					Rosmanol	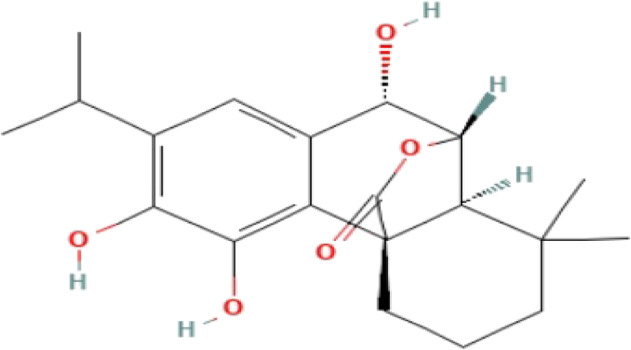
					Cirsimaritin	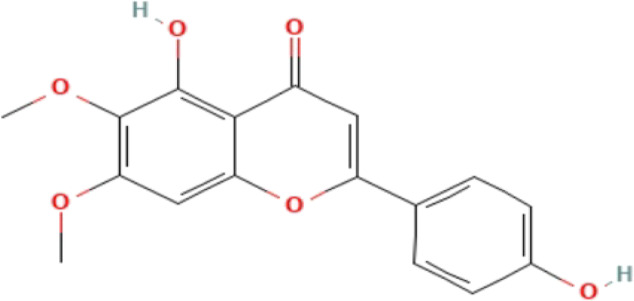
*Sceletium tortuosum*	Loria et al. (2014)	Leaves	Reduced pressure distillation	HPLC fingerprinting analysis	Mesembrine	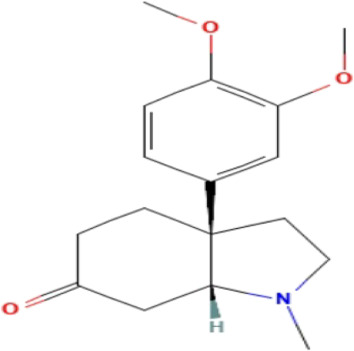
	Harvey et al. (2011)	Above-ground parts	Maceration	Filtration and column chromatography	Mesembrenone	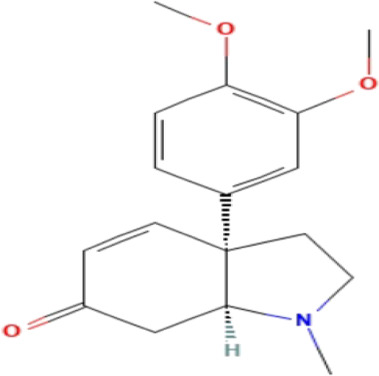
					Mesembrenol	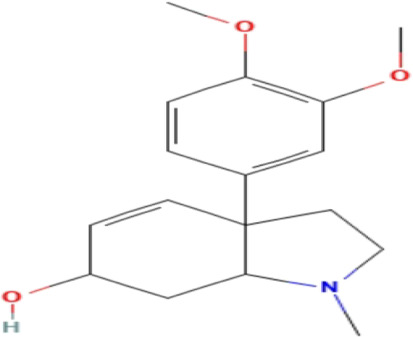

TLC, Thin layer chromatography; VLC, Vacuum liquid chromatography; HPLC, High-performance liquid chromatography; GC/MS, Gas chromatography-mass spectrometry. Molecular structures for compounds were exported from PubChem https://pubchem.ncbi.nlm.nih.gov/.

The most frequently used solvent for extraction of plant material was ethanol, being used in six out of the 12 studies (50%), while the most popular method of extraction was maceration, which was used in four of the studies (33.3%). Various methods were used to isolate and identify phytochemicals from plant extracts, and these include HPLC–UV separation, bioassay-guided fractionation, vacuum liquid chromatography, gas chromatography, column chromatography and preparative thin layer chromatography. Using bioassay-guided fractionation on VLC and preparative TLC, Sandager et al. (2005) isolated buphanidrine and buphanamine from the leaves of *Boophone disticha* and tested them for their affinity to the SERT protein. These phytochemicals inhibited affinity to the SERT in the rat brain. In addition, Neergaard et al. (2009) isolated buphanamine, buphanisine, crinine, buphanidrine and distichamine by repeated preparative HPLC and tested the activity of these compounds in a SERT binding and a functional SERT inhibition assay. Buphanamine, buphanidrine and distichamine showed high activity in SERT binding assay, whereas buphanidrine and distichamine showed activity in the functional SERT inhibition assay. El-Alfy et al. (2010) used preparative C_18_ HPLC to isolate Δ^9^-tetrahydrocannabinol (Δ^9^-THC), cannabidiol (CBD) and cannabichromene (CBC) from the buds of *Cannabis sativa*, the three phytochemicals that showed antidepressant-like effects in two animal models of depression (the force swimming test and the tail suspension test) conducted in the same study. Other phytochemicals isolated from indigenous plant extracts in similar studies, such as (R)-Citronellal, asiaticoside, adhyperforin, hypericin, loliolide and carnosic acid, have been investigated in various models of depression, where they showed positive antidepressant-like results (Öztürk et al., 1996; Neergaard et al., 2010; Kalshetty et al., 2012; Victoria et al., 2014; Sasaki et al., 2021). The use of phytochemicals in MDD therapy is reported to decreases the risk of some severe disorders, including cardiovascular, autoimmune and neurodegenerative diseases (Lee and Bae, 2017).

### Ethnopharmacological Investigations

For the analysis of pharmacological literature, we critically assessed the experimental approaches used, following methods described by Heinrich et al. (2020). Briefly, we looked at the correct identification of plant material under study, appropriate methodology, models, and controls. For a pharmacological study to be included in this review, it had to include a validated source of material, standard methodology for herbal antidepressant assays and access to full text articles written in the English language. Based on the inclusion criteria for this review, a total of 27 medicinal plants were investigated pharmacologically for their antidepressant activity. However, the majority of these plants were screened for antidepressant-like effects despite the absence of any ethnobotanical records indicating their use for such purposes in South African traditional medicine. Plants such as *Agapanthus campanulatus*, *Haemathus coccineus, Scadoxus puniceus*, and *Mondia whitei* have been confirmed to possess antidepressant effects, however, they are documented ethnobotanically to be administered in the treatment of mental disorders, insanity, headache, nervous disorder, used as tranquilizers, snuff and sedatives (Table 2). Out of the 186 medicinal plants recorded in this review (Supplementary Table S1), only 18 plants (9.7% of the total recorded plants) were recorded with indications for managing depression in ethnobotanical surveys. This has led to a paucity in pharmacological evidence on the antidepressant-like effects of South African medicinal plants.

Several biological assays have been used to investigate the antidepressant-like effects of South African medicinal plants, including *in vitro* biological assays (Nielsen et al., 2004; Sandager et al., 2005; Neergaard et al., 2009; Harvey et al., 2011; Stafford et al., 2013) and *in vivo* assays conducted using rodent models of depression (Machado et al., 2007; Machado et al., 2009; El-Alfy et al., 2010; Ahmadpoor et al., 2019; Rabiei and Setorki, 2019). The mechanism of action of most psychoactive compounds involves endocrine modulation of specific molecules in the CNS, modification of multiple biological effects on reuptake and/or receptor binding of various monoamines and interacting with neuronal receptors (Saki et al., 2014; Alrashedy and Molina, 2016). Medicinal plants and their bioactive compounds producing antidepressant therapeutic effects *via* interaction with serotonergic systems (SERT), noradrenergic (NAT) and dopaminergic (DAT) receptors (Machado et al., 2007; Stafford et al., 2008). Therefore, screening plant extracts for the effects they have on these neurotransmitters remain relevant for exploring plants used in traditional medicine for treating depression. Furthermore, the forced swimming test (FST) and tail suspension test (TST) represent the most widely used and well-established paradigm for screening medicinal plants for antidepressant activity using animals (Pedersen et al., 2008).

#### *In Vitro* Biological Assays (SERT, DAT and NAT Screening Assays)

*In vitro* assays utilized for the investigation of potential antidepressant effects of medicinal plants include the SERT (serotonin transporter) protein binding assay, the functional SERT uptake inhibition assay, the functional DAT (dopamine transporter) uptake inhibition assay and the functional NAT (noradrenalin transporter) uptake inhibition assay. The SERT binding assay includes, briefly, mixing a dilution of the extract with [3H] citalopram and rat brain tissue suspension, replacing the extract with paroxetine for positive control and with a buffer for negative control. All samples are then incubated for 2 h and filtered under vacuum before radioactivity is measured by liquid scintillation (Nielsen et al., 2004). For the functional inhibition of SERT, DAT and NAT, human SERT, NAT and DAT clones transfected in COS-7 cells are incubated for 30 min in PBSCM containing 50 nM [3H]-5-HT (SERT assay) or 50 nM [3H]-dopamine (NAT and DAT assays) and increasing concentrations of extracts. The amount of accumulated [3H]-5-HT or [3H]-dopamine is determined by solubilizing cells in scintillant, followed by direct counting of plates. Specific uptake is calculated by subtracting uptake values from control values (Pedersen et al., 2008).

Nine plants including *Agapanthus campanulatus*, *Boophone disticha*, *Datura ferox* L., *Hypericum perforatum* and *Mondia whitei* were investigated for their antidepressant activity using *in vitro* models of depression (Table 4). Most of the *in vitro* investigations on the potential antidepressant effects of medicinal plants was conducted using the SERT binding assay. The pharmacological screening of medicinal plants for antidepressant effects has yielded several plants with noteworthy antidepressant activity. Ethanol extracts from the leaves of *Agapanthus campanulatus, Boophone disticha* and *Mondia whitei* exhibited antidepressant activity *in vitro* (Table 4). Methanol and ethanol extracts from the bulbs of *Boophone disticha, Haemathus coccineus* and *Scadoxus puniceus* showed affinity for the SERT protein *in vitro*. Most of these active leaf and bulb extracts were extracted using ethanol as a solvent. Generally, ethanol extracts were observed to have the most significant antidepressant activity. Pharmacological studies on *Boophone disticha* revealed that ethanolic extracts from this plant possess affinity to the SERT protein, and functional inhibition of SERT, DAT and NAT (Nielsen et al., 2004; Sandager et al., 2005; Pedersen et al., 2008; Neergaard et al., 2009). Powdered extracts of *Hypericum perforatum* are used traditionally as antidepressants and represent an accepted alternative to conventional synthetic antidepressants (Van Wyk et al., 1997; Nielsen et al., 2004). *In vitro* pharmacological investigations on *H. perforatum* reported that this plant possesses affinity to SERT, and it inhibited SERT, NAT and DAT (Fiebich et al., 2011; Tian et al., 2014). The observed antidepressant pharmacological activities of *H. perforatum* appear to be attributed to adhyperforin, hypericin and pseudohypericin, previously isolated from dry extracts, with antidepressant activities as effective as conventional antidepressants desipramine and trimipramine (Öztürk et al., 1996; Tian et al., 2014). An ethanol extract from *Sceletium tortuosum*, with alkaloids mesembrine, mesembrenone and mesembrenol, has shown to inhibit serotonin uptake (Harvey et al., 2011).

**TABLE 4:** Different *in vitro* assays utilized for investigating the antidepressant potential of medicinal plants.

Plant species	Reference	Plant part	Solvent used	Model	Serotonin transporter (SERT) binding assay	SERT inhibition assay	Dopamine transporter (DAT) inhibition assay	Noradrenalin transporter (NAT) inhibition assay
*Agapanthus campanulatus*	Pedersen et al. (2008)	Unspecified	Ethanol	Whole rat brain; Human SERT clones; Human DAT clones	Extracts inhibited the binding of [3H]-citalopram with IC_50_ value of 4.9 ± 1.3 mg dry extract/ml	The extract inhibited SERT significantly with IC_50_ = 99.4 μg/ml	Extract inhibited DAT significantly with IC_50_ = 76.2 μg/ml	Extract exhibited potent inhibition of NAT, with IC_50_ = 84.9 μg/ml
	Nielsen et al. (2004)i	Leaves; flowers	Ethanol and water	Rat brains from male Wistar rats	Aqueous extracts of leaves and flowers from *A. campanulatus* had more than 60% transport protein bound [3H]citalopram at the three highest concentrations	—	—	—
*Boophone disticha*	Pedersen et al. (2008)	Unspecified	Ethanol	Whole rat brain; Human SERT clones; Human DAT clones	Extracts inhibited the binding of [3H]-citalopram with IC_50_ = 0.5 ± 1.5 mg/ml	The extract inhibited SERT with IC_50_ = 423.8 μg/ml	Extract inhibited DAT, with IC_50_ = 93.5 μg/ml	Extract had potent inhibition of NAT, with IC_50_ = 77.3 μg/ml
	Sandager et al. (2005)	Leaves	Ethanol	Rat brain	Buphanamine and buphanadrine showed affinity to the SERT	—	—	—
	Nielsen et al. (2004)	Leaves; bulbs	Ethanol and water	Rat brains from male Wistar rats	Extracts displaced more than 50% of the transport protein bound [3H]citalopram at the three highest concentrations	—	—	—
	Neergaard et al. (2009)	Bulbs	Ethanol	Rat brain; human COS-7 cells	Buphanamine, buphanidrine and distichamine were the most active (IC_50_ = 55 ± 4 μM (Ki = 23 μM), 63 ± 9 μM (Ki = 26 μM) and 65 ± 7 μM (Ki = 27 μM), respectively)	Buphanidrine and distichamine showed activity in the functional assay	—	—
*Datura ferox*	Nielsen et al. (2004)	Seeds	Ethanol and water	Rat brains from male Wistar rats	Extract had 80% transport protein bound [3H]citalopram at the three highest concentrations	—	—	—
*Haemathus coccineus*	Stafford et al. (2013)	Bulbs	Methanol	Rat brains from male Wistar rats	Extracts had considerable affinity for the SERT, with IC_50_ = 2 μg/ml	—	—	—
*Hypericum perforatum*	Tian et al. (2014)	Unspecified	Unspecified	Cell membranes; Rat synaptosome	Adhyperforin had strong binding affinity to the hSERT with Ki value = 18.75 ± 7.76 mg/ml	Adhyperforin potently blocked the uptake of 5-HT, with IC_50_ = 4.14 ± 0.29 mg/ml	Adhyperforin potently blocked the uptake of DA, with IC_50_ = 0.89 ± 0.07 mg/ml	Adhyperforin potently blocked the uptake of NE, with IC_50_ = 2.64 ± 0.35 mg/ml
	Fiebich et al. (2011)	Aerial parts	Ethanol	Rat cerebral cortex	*Hypericum* extracts (500 μg/ml) inhibited serotonin uptake by greater than 80%	—	—	—
*Mondia whitei*	Pedersen et al. (2008)	Unspecified	Ethanol	Whole rat brain; Human SERT clones; Human DAT clones; Human NAT clones	Extracts inhibited the binding of [3H]-citalopram with IC_50_ value of 2.2 ± 1.4 mg dry extract/ml	The extract inhibited SERT with IC_50_ = 283 μg/ml	No significant effect on this transporter	No significant effect on this transporter
	Neergaard et al. (2010)	Leaves	Ethanol	Rat brain	Fractions containing (−)-loliolide showed good displacement of [3H]-citalopram from the SERT	—	—	—
*Scadoxus puniceus*	Bay-Smidt et al. (2011)	Bulbs	Methanol	Rat brains, except cerebellum	Showed affinity to the SERT protein with IC_50_ >50 μg/ml	—	—	—
*Sceletium tortuosum*	Harvey et al. (2011)	Above-ground parts	70% ethanol, 30% water	5-HT1 receptors; DAT receptors	Extract had a marked effect (>80% inhibition of binding) at the 5-HT transporter binding site	No significant effect on this transporter	No significant effect on this transporter	No significant effect on this transporter
	Coetzee et al. (2016)	Unspecified	Unspecified	Human astrocytes and GT1-7 cells	—	In astrocytes, extract had comparable effects to citalopram. In GT1-7 cells, similar effects to citalopram, but came by slower	—	—
*Xysmalobium undulatum*	Pedersen et al. (2008)	Unspecified	Ethanol	Whole rat brain; Human SERT clones; Human DAT clones; Human NAT clones	Extracts inhibited the binding of [3H]-citalopram with IC_50_ = 1.1 ± 2.3 mg dry extract/ml	No significant effect on this transporter	No significant effect on this transporter	No significant effect on this transporter
	Nielsen et al. (2004)	All parts	Ethanol and water	Rat brains from male Wistar rats	Extract had more than 50% of the transport protein bound [3H]citalopram at the three highest concentrations	—	—	—

### *In Vivo* Biological Models

*In vivo* behavioral models such as the forced swimming test (FST), tail suspension test (TST) and the open-field test (OFT) were applied on *in vivo* models the investigation of potential antidepressant effects of medicinal plants. The forced swimming test is the most widely used test for screening of antidepressants and it involves placing rats or mice individually in plastic cylinders containing a column of water with no possible escape for 6 min (Rabadia et al., 2013). After allowing the rats to acclimatize for 2 min, immobility time (in sec) is recorded in the last 4 min of the test (Ahmadpoor et al., 2019). In the tail suspension test, rats or mice are individually hung by the tail using adhesive tape and attached to the edge of a tabletop hanging about 75 cm above the floor. The total duration of immobility (the absence of any limb or body movements) is then recorded manually during the 6 min session (Pedersen et al., 2008). Differences that can be noted between the FST and the TST the response to drugs in both tests and the apparent increased sensitivity of the TST (Machado et al., 2009). The locomotor activity test involves recording the spontaneous activity of animals treated with the plant extracts in photoresistor actometers. Mice or rats are individually placed in actometers illuminated by two light beams for the recording of light beam interruptions and the number of light beam crossings are counted (Pedersen et al., 2008). Imipramine was used for positive control and Tween 80:water (1:10) was used as negative control in all the three *in vivo* studies (Pedersen et al., 2008).

A total of 24 plants, including *Adiantum capillus-veneris*, *Agapanthus campanulatus*, *Albizia adianthifolia*, *Artemisia dracunculus*, *Boophone disticha* and *Cannabis sativa* have been investigated in *in vivo* models (Table 5). Most of the *in vivo* studies were conducted using the rodent forced swimming test. Ethanol extracts from *Albizia adianthifolia, Rosmarinus officinalis, Sceletium tortuosum* and *Ziziphus mucronata* exhibited antidepressant activity *in vivo*. Ethanolic extracts from *Boophone disticha* exhibited antidepressant-like effects in the FST and TST (Pedersen et al., 2008). Previously isolated from *B. disticha*, buphanamine and buphanadrine structurally have the benzo-1,3-dioxole moiety in common with SSRI paroxetine, which could explain the observed anti-depressant-like properties (Sandager et al., 2005; Neergaard et al., 2009; Stafford et al., 2013), supporting the traditional use of this plant to treat mental disorders (Hutchings et al., 1996). *Cannabis sativa* cannabinoids Δ^9^-THC, CBD and CBC induced significant antidepressant-like effects in animal models of depression (FST and TST) (El-Alfy et al., 2010; Zanelate et al., 2010). A study into CBD-induced antidepressant-like effects in the FST revealed that the antidepressant effects depend on levels of serotonin, but not noradrenaline, in the CNS (Zanelate et al., 2010; Sales et al., 2018). This serves as rationale for the traditional use of *C. sativa* to treat depressive mental conditions (Hutchings et al., 1996; Van Wyk et al., 1997). Oleuropein, which is considered the most active phenolic in ingredient *Olea europaea* subsp. *cuspidata*, significantly reduced levels of serotonin and dopamine and exhibited antidepressant-like effects in the FST, TST and OFT (Badr et al., 2020).

**TABLE 5:** Different *in vivo* assays utilized for investigating the antidepressant potential of medicinal plants.

Plant species	References	Plant part	Solvent used	Model	Forced swimming test (FST)	Tail suspension test (TST)	Open-field test (OFT)
*Adiantum capillus-veneris*	Ahmadpoor et al. (2019)	Whole plant	Ethanol	Male BALB/c mice (25–30 g)	Plant extract (100, 200, and 400 mg/kg) significantly decreased the immobility time	—	—
	Rabiei and Setorki (2019)	Whole plant	Ethanol	Male rats (250–300 g)	Extract (200 mg/kg) significantly reduced immobility time duration while doses of 50 and 100 mg/kg had no significant effect on immobility	—	—
*Agapanthus campanulatus*	Pedersen et al. (2008)	Unspecified	Ethanol	Male albino Swiss mice (25–30 g)	Extract exhibited antidepressant-like effects at doses 250 and 500 mg/kg and yielded 74.4 and 62.3% relative immobility, respectively	No significant effects	No effect on the spontaneous activity of mice or rats
	Pedersen et al. (2008)	Unspecified	Ethanol	Male Wistar rats (220–300 g); Male C57BL/6J mice (19–27 g)	Extract exhibited no significant effects	—	—
*Albizia adianthifolia*	Aderibigbe et al. (2018)	Leaves	Ethanol	Male Swiss mice	Extract significantly reduced immobility time at doses 1.25 mg/kg (40.8 ± 13.1) and 2.50 mg/kg (42.4 ± 9.7) compared to control (170.0 ± 10.1) [*p* ˂ 0.05]	Extract significantly reduced immobility time at dose1.25 mg/kg (85.2 ± 8.9) compared to control (142.6 ± 3.9) [*p* ˂ 0.05]	—
	Beppe et al. (2015)	Leaves	Distilled water	Wistar rats (350 ± 50 g)	Both doses exhibited significant effects evidenced by the swimming time (F(3,36) = 21.09, *p* < 0.0001) and the immobility time (F(3,36) = 78.59, *p* < 0.0001)	—	—
*Artemisia dracunculus*	Jahani et al. (2019)	Aerial parts	Ethanol	Male Swiss mice and male NMRI mice (8–12 weeks; 18–25 g)	At doses 100, 200, and 400 mg/kg, ethanolic extract decreased immobility time (162.30 ± 6.87, 161.60 ± 5.54, and 153.60 ± 6.87 s, respectively)	Extract at the doses of 100 and 200 mg/kg decreased immobility time (124.00 ± 6.58, 117.20 ± 2.50 s, respectively). No significant effect at dose 400 mg/kg	Extract decreased immobility time in Swiss mice treated with 100 mg/kg
	Ilkhanizadeh et al. (2021)	Whole plants	Ethanol	Adult female NMRI mice	Extract (50 mg/kg) significantly reduced depression induced immobility time in comparison to OVX group (*p* < 0.05)	Extract (50 mg/kg) significantly reduced depression induced immobility time (*p* < 0.05)	Extract at doses 25 and 50 mg/kg significantly increased the number of crossing in OFT test
*Boophone disticha*	Pedersen et al. (2008)	Unspecified	Ethanol	Male albino Swiss mice (25–30 g)	Extract exhibited an antidepressant-like activity at doses 250 and 500 mg/kg and yielded 84.9 and 83.3% relative immobility, respectively	At a dose of 125 mg/kg, extract significantly exhibited an antidepressant-like activity	No effect on the spontaneous activity of mice or rats
	Pedersen et al. (2008)	Unspecified	Ethanol	Male Wistar rats (220–300 g); Male C57BL/6J mice (19–27 g)	Extract exhibited an antidepressant-like activity at dose 250 mg/kg and yielded 74.2% relative immobility	—	—
*Cannabis sativa*	Zanelate et al. (2010)	Unspecified	Unspecified	Male Swiss mice (20–25 g)	CBD treatment reduced immobility time (F6,59 = 3.89, *p* < 0.01) at dose 30 mg/kg	—	—
	El-Alfy et al. (2010)	Buds	Hexane and water	8 weeks old male Swiss Webster mice (24–30 g); 8 weeks old adult male DBA/2 mice (19–23 g)	Δ9-THC showed significant overall reduction in immobility time (F[3,35] = 8.32; *p* = 0.0003). Δ8-THC had no significant effect on immobility time (F[3,44] = 2.14; *p* = 0.11). CBD revealed a significant decrease in immobility time (F[3,42] = 3.89; *p* = 0.015)	Δ9-THC resulted in significant decrease in immobility time (F[3,32] = 3.29; *p* = 0.033). CBC resulted in significant reduction in immobility time (F[3,33] = 6.24; *p* = 0.002). CBD did not affect immobility time at any of the doses (F[3,33] = 0.59; *p* = 0.623)	—
	Sales et al. (2018)	Unspecified	Unspecified	Male Swiss mice (8 weeks)	CBD at dose 10 mg/kg significantly reduced the immobility time in the FST (F3,25 = 6.104, *p* < 0.05)	—	No significant effect on the locomotor activity in the open-field test
*Centella asiatica*	Selvi et al. (2012)	Whole plant	Ethanol and distilled water	Male Wister rats (8 weeks old)	The extract at both doses exhibited significant reduction in immobility time compared to control	—	—
	Ceremuga et al. (2015)	Unspecified	Unspecified	Male Sprague Dawley rats (250–300 g)	Asiatic acid (AA) from *Centella asiatica* had no significant effect on immobility time. Significant effect (1.8), (*p* = 0.001) in the faecal pellet output (FPO)	—	—
	Kalshetty et al. (2012)	Leaves	5 L of isopropyl alcohol	Male Sprague Dawley rats (250–270 g)	—	—	INDCA (3, 10 or 30 mg/kg) significantly reduced the ambulation scores (30.3, 51.2 and 64.7%; *p* < 0.001) in the open filed test. INDCA (30 mg/kg) showed significant (*p* < 0.01) reduction of rearing score (12.8, reduction of 47.5%). INDCA (30 mg/kg) treatment reduced the grooming score by 33.3%
*Cinnamomum camphora*	Rabadia et al. (2013)	Bark	Ethanol	Swiss albino mice (18–30 g)	Rats showed significant decrease in their immobility times at all three doses (116.7 ± 6.146, 110.8 ± 12.54; *p* < 0.05 and 101.7 ± 9.458; *p* < 0.01), respectively	Rats showed significant decrease in their immobility times at all three doses (133.3 ± 8.758, 128.3 ± 8.33; *p* < 0.05 and 122.5 ± 8.342; *p* < 0.01), respectively	—
*Cymbopogon nardus*	Victoria et al. (2014)	Unspecified	Ethanol	Adult male Swiss albino mice (25–35 g; 2–3 months old)	No significant effects in the mouse FST.	No significant effects on mouse TST	No significant effect on exploratory and locomotor activities of mice
*Datura stramonium*	Devi et al. (2012)	Leaves	Distilled water	Adult male albino mice (26–32 g)	Extract caused a significant increase in immobility time (*p* ˂ 0.05 for 20 mg/kg, *p* ˂ 0.01 for 40 mg/kg)	—	The extract significantly decreased the locomotor activity when compared with the control. For 20 mg/kg (*p* < 0.05) and for 40 mg/kg (*p* < 0.001)
*Hoodia gordonii*	Citó et al. (2015)	Unspecified	Unspecified	Male Swiss mice (25–30 g)	Significantly reduced immobility time of animals after acute administration of *H*. *gordonii* at doses of 25 and 50 mg/kg. Animals treated for 15 consecutive days at doses of 25 mg/kg and 50 mg/kg also reduced immobility time	—	Extract did not alter locomotor activity when either dose was used
*Hypericum perforatum*	Tian et al. (2014)	Unspecified	Unspecified	Male Swiss mice (18–22 g)	Adhyperforin (16 mg/kg) significantly decreased the immobility times of mice in the FST (*p* = 0.049)	Adhyperforin (16 mg/kg) significantly reduced the immobility times of mice (*p* = 0.043)	Adhyperforin had no significant effect on spontaneous locomotor activity in mice at any dose tested
	Öztürk et al. (1996)	Aerial parts	50% ethyl alcohol	Adult male albino mice (30–45 g)	Dried plant extract from aerial parts of *Hypericum perforatum* decreased the swimming performance to a statistically significant extent (*p* < 0.05)	—	Dried plant extracts from the aerial parts of *Hypericum perforatum* decreased the walking time to a statistically significant extent (*p* < 0.05)
	Ramalhete et al. (2016)	Aerial parts	Methanol	Male hsd:icr mice (28–34 g)	Extract showed no significant effects	Extract showed non-significant effects like those of the control	—
	Fiebich et al. (2011)	Aerial parts	Ethanol and water	Unspecified	*Hypericum* extract exerted a significant inhibitory effect on the Immobility of the rats at doses 180 and 360 mg/kg (*p* < 0.01)	—	—
*Hypericum revolutum*	Ejigu and Engidawork, (2014)	Leaves	Methanol	Wistar male rats (7–10 weeks, 180–230 g); Male and female Swiss Albino mice (7–10 weeks, 22–32 g)	Extract significantly brought down immobility time at both 200 mg/kg (33.72%, *p* < 0.05) and 400 mg/kg (38.42%, *p* < 0.01)	Extract at doses of 200 (44%, *p* < 0.01) and 400 mg/kg (49%, *p* < 0.01) significantly reduced immobility time, 100 mg/kg did not show any significant change	Extract failed to produce any significant alteration in the parameters measured in the OFT
*Maerua angolensis*	Benneh et al. (2018)	Stem bark	Petroleum ether/ethyl acetate (50:50) mixture	Male ICR mice (20–25 g)	*Maerua angolensis* extract (1000 mg/kg) significantly decreased the immobility time and increased swimming time (*F*3, 38 = 10.33, *p* < 0.0001) of mice	*Maerua angolensis* extract at 1000 mg/kg significantly decreased the immobility time (*F*3 20 = 5.744, *p* = 0.0053). Significant effect on duration at dose 300 mg kg (*F*3, 20 = 3.493, *p* = 0.0347)	—
*Melia azedarach*	Ishaq, (2016)	Flowers, twigs and roots	Methanol	Male and female NMRI mice (20–30 g)	15 mg/kg dose significantly (22.7%, *p* < 0.005) decreased immobility time	—	—
*Mentha spicata*	Jedi et al. (2017)	Unspecified	Unspecified	Male mice	Essential oil (120 and 240 mg/kg) reduced immobility time in mice	Essential oil at doses 120 7 240 mg/kg reduced immobility time in mice	—
*Mondia whitei*	Pedersen et al. (2008)	Unspecified	Ethanol	Male albino Swiss mice (25–30 g)	Extract exhibited no significant effects	No significant effects	No effect on the spontaneous activity of mice or rats
	Pedersen et al. (2008)	Unspecified	Ethanol	Male Wistar rats (220–300 g); Male C57BL/6J mice (19–27 g)	Extract exhibited an antidepressant-like activity at dose 250 mg/kg and yielded 69.9% relative immobility	—	—
*Olea europaea* subsp. *cuspidata*	Badr et al. (2020)	Unspecified	Unspecified	Male mice (20–25 g)	Immobility times were significantly reduced by Oleuropein at 8 mg/kg (*p* < 0.01), 16 mg/kg (*p* < 0.001) and 32 mg/kg (*p* < 0.001)	Oleuropein treatment at doses 8, 16 and 32 mg/kg significantly decreased the immobility time in TST	Immobility time significantly decreased with oleuropein at 8 mg/kg (*p* < 0.01), 16 mg/kg (*p* < 0.001) and 32 mg/kg (*p* < 0.001)
	Perveen et al. (2013)	Unspecified	Unspecified	Male albino Wistar rats (140–280 g)	Significant increase in struggling time (*p* < 0.05) following repeated administration of extra-virgin olive oil	—	—
	Tariq et al. (2021)	Fruit	Ethanol	Sprague–Dawley rats (200–250 g)	Green and black olive extract significantly reduced the immobility period	Green and black olive extract treatment displayed less immobility time in TST	—
*Rosmarinus officinalis*	Machado et al. (2009)	Stems and leaves	Ethanol	Male Swiss mice (60–80 days old; 40–50 g)	The percent of reduction in the immobility time was 49.5% in FST with one-way ANOVA revealing a significant effect of the extract (100 mg/kg) in FST [F(4,31) = 6.24, Pb0.01]	The percentage of reduction in immobility time was 22.9%, 28.0% in TST, one-way ANOVA revealed a significant effect of the extract (10–100 mg/kg) in TST [F(4,29) = 6.80, Pb0.01]	Extract (1–300 mg/kg, p.o.) did not significantly alter the number of the rearings of mice in OFT
	Sasaki et al. (2013)	Leaves	Ethanol	Male ICR mice (3 weeks old; 35–40 g)	—	Immobility times were decreased to 91.98 ± 12.06 s for 50 mg/kg and 66.81 ± 17.00 s for 100 mg/kg	—
	Sasaki et al. (2021)	Whole plant	Ethanol and citric acid crystal	Male ICR mice (8 weeks old)	—	While 100 mg/kg significantly decreased immobility time from day 2 onwards, 10 mg/kg only had decrease in immobility time at day 7	—
	Abdelhalim et al. (2015)	Whole plant	Petroleum ether, ethanol and ethyl acetate	Male Swiss mice (20–30 g)	Salvigenin, rosmanol and cirsimaritin (30 and 100 mg/kg) significantly decreased the immobility time in the FST as compared to the control (vehicle) (*p* < 0.05; *p* < 0.001)	Salvigenin, rosmanol and cirsimaritin (30 and 100 mg/kg) caused a significant decrease in the immobility time as compared to the vehicle control group (*p* < 0.05; *p* < 0.01)	—
*Securidaca longipedunculata*	Adebiyi et al. (2006)	Roots	Distilled water	Swiss albino mice (25–30 g)	Aqueous extract significantly decreased duration of immobility (*p* < 0.05) at the dose of 400 mg/kg	—	—
*Sceletium tortuosum*	Loria et al. (2014)	Leaves	Methanol and chloroform	Male Sprague Dawley rats (175–200 g, 6–7 weeks old	*S. tortuosum* extract significantly decreased floating time of rats	—	—
*Schinus molle*	Machado et al. (2007)	Stems, leaves	Hexane	Male Swiss mice (35–45 g)	—	Extract significantly decreased the immobility time at all doses. One-way ANOVA revealed a significant effect [F(4,25) = 11.14, Pb0.01]	Extract at dose range 100–600 mg/kg had no significant effect on locomotor activity of mice as compared to control group. One-way ANOVA revealed [F(3,18) = 1.38, *p* = 0.27]
*Xysmalobium undulatum*	Pedersen et al. (2008)	Unspecified	Ethanol	Male albino Swiss mice (25–30 g)	Extract exhibited an antidepressant-like activity at doses of 250 and 500 mg/kg and yielded 77.6% 67.9% relative immobility, respectively	No significant effects	No effect on the spontaneous activity of mice or rats
	Pedersen et al. (2008)	Unspecified		Male Wistar rats (220–300 g); Male C57BL/6J mice (19–27 g)	Extract exhibited no significant effects	—	—
*Ziziphus mucronata*	Keugong Wado, et al. (2020)	Leaves	Methanol and distilled water	Adult male Wistar rats	Chronic treatment with *Ziziphus mucronata* at both doses significantly reversed depressive behavior ([F (5, 27) = 6.284; *p* < 0.0005] lowering in swimming time and [F (5, 27) = 10.44; *p* < 0.0001] increase in immobility time)	—	—

The most extensively studied plants included *Agapanthus campanulatus*, *Boophone disticha*, *Hypericum perforatum*, *Mondia whitei* and *Xysmalobium undulatum*. These plants were investigated in all the bioassays included in this study. Based on the plant parts screened, leaf extracts for 11 plants (*Agapanthus campanulatus, Boophone disticha, Mondia whitei, Albizia adianthifolia, Centella asiatica, Rosmarinus officinalis, Sceletium tortuosum, Hypericum revolutum, Datura stramonium, Schinus molle* and *Ziziphus mucronata*) demonstrated antidepressant activity. These studies serve as scientific rationale for the utilization of these pharmacological assays. Furthermore, we provides insight on the plant parts and solvents used in the preparation of anti-depressive plant extracts for pharmacological investigation.

### Toxicity and Safety of South African Plants Used for Managing Depression

South Africa has a rich diversity of medicinal plants, however, this rich flora includes several plants with the potential to poison humans (Nair and Van Staden, 2013; Ndhlala et al., 2013). As highlighted in Table 6, there are safety concerns for some of the South African plants with anti-depressant potential. Among the listed plants, *Boophone disticha, Haemanthus coccineus* and *Scadoxus puniceus* have the isoquinoline alkaloids as the toxic compound characterized by symptoms such as dizziness (Ndhlala et al., 2013). In South Africa, traditional medicine prescription and use was not regulated in the past. It is also associated with the dangers of misadministration, and the potential long-term genotoxic effects that follow the prolonged use of self-prescribed popular herbal medicines (Fennell et al., 2004). Poisonous plants can lead to symptoms such as serious poisoning when ingested or irritation and/or discomfort after contact with the skin (Hutchings et al., 1996; Van Wyk et al., 1997). Adverse effects may include hallucinations, sedation, irrational behavior and more seriously coma and death (Van Wyk et al., 1997). Toxic substances from medicinal plants can affect important human organs while some are able to affect functional systems of the body, like the central nervous system (CNS), and interfere with the nerve function coordination of the body (Ndhlala et al., 2013). A comprehensive review on the ethnopharmacology and toxicology of alkaloids of the Amaryllidaceae of South Africa has been published (Nair and Van Staden, 2013). *Boophone disticha* is amongst the first recorded toxic plants to have caused fatalities due to poisoning after consumption by Sotho, Xhosa and Zulu people from South Africa or when its bulbs were used as arrow poisons by the Khoi and San, and is the most widely studied for its toxic effects (Hutchings et al., 1996; Nair and Van Staden, 2013). Many injuries have resulted from the toxic use of this plant (Sobiecki, 2002). Symptoms of non-fatal administration of this plant include an unsteady gait, dryness of the mouth and increased thirst, nausea, vomiting, impaired vision and variable emotional reactions followed by stupor and sleep for about an hour (Hutchings et al., 1996). The prolonged use of *Cannabis sativa* has toxic effects that include indifference, insomnia, lassitude, headaches, increased susceptibility to infections, sexual impotence, gastrointestinal disturbances, and personality changes (Hutchings et al., 1996). Side effects of regular use include heart palpitations, orthostatic hypotension, acute panic reactions, mental confusion, depression paranoia and acute toxic psychosis in some users (Hutchings et al., 1996). The seeds of *Cinnamomum camphora*, commonly known as camphor bush, contain cytotoxic proteins camphorin and cinnamomin that cause systemic toxicity when used as an inhalant for children, or in large amounts (Hutchings et al., 1996; Van Wyk et al., 1997).

**TABLE 6:** Toxicity, side effects and safety indication of South African medicinal plants used traditionally to manage depression and related ailments.

Plant species	Toxicity	Side effects	Safety indication	References
*Agapanthus campanulatus*	No reported toxicity	Gastrointestinal tract and kidney problems	—	Ndhlala et al. (2013)
*Albizia adianthifolia*	No reported toxicity	No reported side effects	Leaf extracts do not induce neurotoxicity and this effect could be related to its antioxidant activity (increased activities of SOD, CAT, GSH level and the decreased levels of protein carbonyl and MDA	Beppe et al. (2015); Hutchings et al. (1996); Van Wyk et al. (1997)
*Artemisia dracunculus*	No reported toxicity	No reported side effects	Possible mechanism for anxiolytic and antidepressant effects reported to be the anti-oxidant activity of tarragon. Extract reduced the serum MDA while elevated SOD and GPX levels and has protective effect against ROS production and oxidative stress	Ilkhanizadeh et al. (2021); Khosravi et al. (2017); Mumivand et al. (2017)
*Boophone disticha*	Bulbs are reported to have caused both acute and fatal poisoning in human beings, following medicinal administration	Symptoms of non-fatal toxicity include dryness of the mouth and increased thirst, nausea, vomiting, impaired vision, and variable emotional reactions followed by stupor and sleep for about an hour	Several human deaths have been reported due to extremely toxic alkaloids. Internal use is dangerous and should be avoided	Hutchings et al. (1996); Ndhlala et al. (2011); Van Wyk and Gericke, (2000); Van Wyk et al. (1997)
*Cannabis sativa*	Toxic effects of prolonged use include lassitude, indifference, lack of productive activity, insomnia, headaches, nystagmus, increased susceptibility to infections, gastrointestinal disturbances, sexual impotence and personality changes	Side effects include increased heart rate, palpitations, orthostatic hypotension, acute panic reactions, mental confusion, depression and paranoia. Acute toxic psychosis in some users	The mouse tetrad assay determined that Δ9-THC at 2.5 mg/kg dose in both the FST and TST does not cause any impairment of locomotor activity, change in body temperature or catalepsy	El-Alfy et al. (2010); Hutchings et al. (1996)
*Centella asiatica*	No reported toxicity	No reported side effects	—	Kalshetty et al. (2012); Van Wyk and Gericke, (2000)
*Cinnamomum camphora*	Death from respiratory causes is rare however use as an inhalant for children, or in large amounts should be avoided as it may cause systemic toxicity. Seeds contain cytotoxic proteins camphorin and cinnamomin	Large doses of this plant cause nausea and vomiting while high doses produce epileptiform convulsions	Small doses warm and soothe the epigastric region	Hutchings et al. (1996); Rabadia et al. (2013); Van Wyk et al. (1997)
*Cymbopogon nardus*	No reported toxicity	No reported side effects	The treatment of mice with (R)-Citronellal did not cause death of any animals and demonstrated lack of toxicological effects	Victoria et al. (2014)
*Datura ferox*	All plant parts are toxic	Pupil dilation, convulsion, tremor and appetite depression. Toxicity in livestock causes reduction in body weight, hypersalivation and an altered gait	—	Kovatsis et al. (1993)
*Datura stramonium*	Poisoning from cooked leaves. Ingestion of the seeds produces similar effects to *Cannabis* poisoning in children. Studies have shown it has potential to cause damage to the ultrastructure of the brain cells	Severe mental confusion, hallucinations, insomnia, increased heart rate and decreased saliva due to atropine, tropane, and hyoscine alkaloids. Blurred vision, suppressed salivation, vasodilation, hallucinations and delirium	There exists dangers of harmful side effects and self-medication without medical advice is not recommended	Devi et al. (2012); Hutchings et al. (1996); Ndhlala et al. (2013); Van Wyk et al. (1997)
*Haemanthus coccineus*	All members of genus *Haemanthus* are considered capable of causing dermatitis	Side effects have not been reported	—	Bay-Smidt et al. (2011); Stafford et al. (2013)
*Hoodia gordonii*	Has anorectic effects (produces loss of appetite)	Experiments resulted in decrease in food consumption and body mass of rats and an increase in ATP in the hypothalamus	—	Van Wyk et al. (1997)
*Hypericum perforatum*	No reported toxicity associated with internal use	Photosensitivity may occur in fair-skinned users, especially under fairly strong sunshine	—	Öztürk et al. (1996); Ramalhete et al. (2016); Tian et al. (2014); Van Wyk et al. (1997)
*Maerua angolensis*	Seizure induction potential in mice	Physical signs such as tonic and/or clonic convulsions were observed during the Irwin test	—	Benneh et al. (2018)
*Melia azedarach*	Significant poisoning may occur after large amounts of the fruit have been ingested. All plant parts are reported to be toxic. Fatal poisoning from the fruit and bark	Anuria, severe stomatitis and violent and sanguineous vomiting, nausea, and diarrhoea, followed by, mental confusion and stupor, respiratory problems, convulsions and partial to complete paralysis	Gastric lavage, egg whites and milk for shock treatment and symptomatic measures are recommended treat poisoning	Hutchings et al. (1996)
*Mondia whitei*	No reported toxicity	No reported side effects	*Mondia* is reasonably expected to be safe when prepared and used according to traditional practices	Aremu et al. (2011); Koorbanally et al. (2000b); Neergaard et al. (2010)
*Olea europaea* subsp. cuspidata	No reported toxicity	Gastric symptoms may occur due to an irritant effect on the mucosa. Therefore, plant medicine should always be taken after meals	Significantly decreased the antioxidant pool of rat’s brain	Hutchings et al. (1996); Tariq et al. (2021)
*Rosmarinus officinalis*	Dose ranges 50–200 mg/kg of Salvigenin, rosmanol and cirsimaritin did not produce any toxicity effects	No reported side effects	Protect neuronal cells against corticosterone-induced toxicity. Extract improved cell viability 30% *in vitro*	Abdelhalim et al. (2015); Sasaki et al. (2013)
*Scadoxus puniceus*	Human deaths and poisoning have been reported	Symptoms of non-fatal poisoning from the bulbs or leaves include visual disturbances, dizziness and CNS excitation or depression. Hypotension and convulsion	—	Ndhlala et al. (2013); Van Wyk et al. (1997)
*Sceletium tortuosum*	No toxicity reported	Intoxicating doses can cause euphoria	No severe adverse effects have been documented	Gericke and Viljoen (2008); Harvey et al. (2011); Van Wyk et al. (1997)
*Securidaca longipedunculata*	Securinine is toxic. Overdoses are potentially lethal and suicidal use has been documented	—	—	Van Wyk and Gericke (2000); Van Wyk et al. (1997)
*Xysmalobium undulatum*	Low toxicity	Severe gastrointestinal irritation from protracted poisoning	—	Van Wyk and Gericke (2000); Van Wyk et al. (1997)
*Ziziphus mucronata*	Low toxicity. Fruit is toxic but is considered edible and is used for porridge. Extracts from leaves have shown potential genotoxic activity	—	—	Hutchings et al. (1996); Van Wyk et al. (1997)

SOD, Superoxide dismutase; GPX, Glutathione peroxidase; CAT, Catalase; GSH, Glutathione; MDA, Malondialdehyde; ROS, Reactive oxygen species.

## Conclusion and Future Perspectives

South Africa has a rich diversity of plants that have been recorded for the treatment of MDD. The systematic review of 20 ethnobotanical records led to the documentation of 186 medicinal plants from 63 families used locally for the treatment of MDD and MDD-com. Most of the plants were from Asteraceae, Fabaceae, Amaryllidaceae and Apocynaceae. These families have demonstrated rationale of their extensive use in traditional medicine given the presence of unique bioactive phytochemicals with antidepressant-like properties in plants belonging to these families. A large number of the literature reviewed listed local uses of medicinal plants for somatic symptoms described as mental health related problems, such as headache, that could be classified as depression based on similar clinical symptoms. The overlap and comorbidity observed between depression and headache in studies using humans provide evidence of a link between medicinal plants used traditionally for headache and the potential antidepressant effects of those plants. This link can be seen with plants such as *Boophone disticha*, *Scadoxus puniceus*, *Maerua angolensis* and *Mentha spicata*, which are listed in ethnobotanical literature as traditional medicine for headache, but also exhibited antidepressant-like effects in pharmacological studies reviewed in this paper. Moreover, *Sceletium tortuosum*, *Xysmalobium undulatum*, *Cannabis sativa*, *Schinus molle* and *Hypoxis hemerocallidea* represent some of the plants with reported ethnopharmacological uses for both depression and headache and have been validated for their antidepressant-like activity, suggesting that medicinal plants used ethnopharmacologically for headache may have some degree of antidepressant-like effects worth investigating.

Despite the extensive use of medicinal plants for MDD-com in traditional medicine, pharmacological evidence validating the antidepressant action of most plants and the search for phytochemicals of anti-depressive novelty are limited. Only 27 plants (14.5%) have been investigated for their antidepressant-like activity in *in vitro* and *in vivo* models of depression. *Agapanthus campanulatus*, *Boophone disticha*, *Hypericum perforatum*, *Mondia whitei* and *Xysmalobium undulatum*, were the most extensively pharmacologically studied based on investigation in all the models of depression reviewed in this paper. A total of nine plants were investigated in four *in vitro* models of depression, while 23 plants were investigated using *in vivo* models of depression. Nine out of the 27 plants, including *Boophone disticha*, *Mondia whitei*, *Cannabis sativa*, *Hypericum perforatum* and *Rosmarinus officinalis* underwent a further phytochemical investigation to identify the bioactive compounds responsible for their antidepressant-like effects. This led to the identification and isolation of 24 phytochemicals, including buphanidrine, buphanamine, distichamine, cannabidiol, asiaticoside, adhyperforin, hypericin and loliolide, of anti-depressive value. On the basis of the pharmacological and phytochemical data reviewed, medicinal plant extracts may contain more novel phytochemicals that can improve antidepressant therapy with a more rapid onset of antidepressant action and minimized side effects. This review provides a useful foundation for the investigation of pharmacological antidepressant-like effects of plants used traditionally without pharmacological validation. Furthermore, it will stimulate the development of new pharmacotherapies derived from plant extracts for use in the clinical treatment of depression. Future research of the antidepressant effects of South African medicinal plants should be explored and validated using additional models of depression with the aim of discovering plant species and novel compounds effective at treating MDD. Simultaneous evaluation of the ethnopharmacology and phytochemistry of medicinal plants used traditionally for depression provides a useful framework for the selection of potential candidate species for drug discovery based on traditional use and the presence of phytochemicals with antidepressant effects. Overall, Asteraceae, Fabaceae, Amaryllidaceae and Apocynaceae may hold plant species with phytochemicals capable of effectively treating MDD and its associated symptoms.
