# Supplementary material for: Antidepressant Effects of South African Plants: An Appraisal of Ethnobotanical Surveys, Ethnopharmacological and Phytochemical Studies
Source: Front Pharmacol. 2022 Jun 29;13:895286. doi: 10.3389/fphar.2022.895286 (PMC9277359; doi:10.3389/fphar.2022.895286)
Supplement: Supplementary file 1 [file DataSheet1.PDF]

Supplementary Material

Supplementary Table 1: An inventory of medicinal plants used against depression and related ailments in South Africa. Species and family names for each plant species were validated in references to The Plant List (www.theplantlist.org), The World Flora Online (http://theworlflora.online) and PlantZAfrica (http://pza.sanbi.org/) and the local names were confirmed using PlantZAfrica (http://pza.sanbi.org/).

Plant family	Scientific name	Local name	Life form	Plant parts used	Method of preparation, route of administration and/or short notes	References
**Aizoaceae**	*Khadia acutipetala* (N.E. Br.) N.E. Br. [*Mesembryanthemum acutipetala* N.E. Br.]	Khadiwortel (A)	Shrub	Rootstock	Unspecified groups use the rootstock as a fermentation agent in beer brewing	Sobiecki (2002)
	Sceletium tortuosum (L.) N.E. Br [*Mesembryanthemum tortuosum* L.]	Kanna (E); Kougoed (A); Kanna (K)	Herb	Whole plant	Used as a psychoactive substance; Emetics made from leaves in boiling water are administered for the fearful dreams; Leaves used to treat headache; Whole plant chewed or drunk for depression and anxiety disorders; Past use as a mood-altering substance from prehistoric times; The dried plant material is prepared traditionally and chewed, smoked, or powdered and inhaled as a snuff; Whole plant used to elevate mood and reduce anxiety and stress	Hutchings *et al.* (1996); Nortje and van Wyk (2015); Philander (2011); Sobiecki (2002); Van Wyk and Gericke (2000); van Wyk *et al.* (1997)

	Trichodiadema stellatum Schwantes [*T. barbatum* (L) Schwantes, *Mesembryanthemum stellatum* Mill.]	Pickle plant (E); Kareemoer (A)	Shrub	Unspecified	Unspecified	Sobiecki (2002)
**Amaryllidaceae**	*Agapanthus campanulatus* F.M. Leight. [*A. campanulatus* subsp. *patens* (F.M. Leight.) F.M. Leight.]	Bell agapanthus (E); Bloulelie (A); Ubani (Z); Leta-la-phofu (S); Ugebeleweni (X)	Herb	Unspecified	Unspecified; Unspecified parts used by the Sotho to treat people with "spirit", which is a type of mental disturbance; Unspecified	Moffett (2016); Sobiecki (2002); Stafford (2009)
	Boophone disticha (L.f.) Herb. [*Amaryllis disticha* L.f., *Brunsvigia disticha* (L.f.) Sweet, *B. toxicaria* (L.f. ex Aiton) Herb.]	Cape poison bulb, sore eye flower (E); Gifbol, seeroogblom (A); Leshoma (S); Incwadi (X); Incotho (Z)	Herb	Bulb	Used as emetics and snuffed or inhaled medicines; Bulb decoctions are administered by mouth to adults suffering from headaches; Unspecified; Unspecified; Bulb infusions are drunk to induce hallucinations and to treat mental diseases; Unspecified; Bulbs are used to treat headache; Weak decoctions of bulb scales administered by mouth or as enemas for headache	Hutchings (1989); Hutchings *et al.* (1996); Moffett (2016); Philander (2011); Sobiecki (2002); Stafford (2009); Van Wyk and Gericke (2000); van Wyk *et al.* (1997)
	Brunsvigia grandiflora Lindl. [*Amaryllis banksiana* Lindl., *A. grandiflora* (Lindl.) D.Dietr., *A. slateriana* Lindl., *Brunsvigia banksiana* (Lindl.) T.Durand & Schinz]	Giant Candelabra flower (E); Reusekandelaaarblom (A)	Herb	Unspecified	Unspecified	Stafford (2009)

	Crossyne guttata (L.) D.Müll.-Doblies & U. Müll.-Doblies [*Amaryllis guttata* L., *Boophone guttata* (L.) Herb.]	Parasol lily, April fool lily (E); Sambreelblom (A)	Shrub	Bulb	Bulb used to treat alcoholism and headaches	Philander (2011)
	Cyrtanthus obliquus (L.f.) Aiton [*C. varius* M.Roem., *Amaryllis pendula* Salisb., *A. umbella* L'Hér. *Crinum obliquum* L.f.]	Knysna lily (E); Knysnalelie (A); Umthaga (Z)	Shrub	Unspecified	Used as emetics and snuffed or inhaled medicines	Hutchings (1989)
	Gethyllis ciliaris (Thunb.) Thunb. [*G. ciliaris* subsp. *ciliaris*, *G. cuspidate* Thunb. ex Baker, *G. polyanthera* Sol. ex Britten]	Hotnotskoekmakranka (A)	Herb	Unspecified	Unspecified	Stafford (2009)
	Haemathus coccineus L. [*H. latifolius* Salisb.]	March flower, paintbrush lily (E); Bergajuin, bloedblom (A); Uzaneke (Z)	Herb	Roots	Boiled root decoctions are taken as emetics	Hutchings *et al.* (1996)
	Scadoxus puniceus (L.) Friis & Nordal [*Haemanthus puniceus* L., *H. rouperi* auct. *H. superbus* Baker]	Paintbrush lily (E); Rooikwas (A); Umgola (Z)	Shrub	Bulbs	Bulbs are used for headaches; Unspecified	Hutchings *et al.* (1996); van Wyk *et al.* (1997)
	Tulbaghia alliacea L.f. [*T. affinis* Link, *T. narcissiflora* Salisb.]	Wild garlic (E); Wildeknoffel, knoffel (A); Sikwa (Z); Sefothafotha (B); Ivimba-mpunzi (X)	Herb	Leaves	Leaf crushed and used as snuff for headache	Nortje and van Wyk (2015)

	Tulbaghia violacea Harv. [*T. violacea var violacea*]	Wild garlic (E); Wildeknoffel (A); Isihaqa (Z); Utswelane (X)	Herb	Unspecified	Used as emetics and snuffed or inhaled medicines	Hutchings (1989)
**Anacardiaceae**	*Schinus molle* L. [*S. angustifolia* Sessé & Moc., *S. huigan* Molina, *S. molle* var. *molle*, *S. occidentalis* Sessé & Moc.]	False pepper tree (E); Peperboom (A)	Tree	Stems, leaves	Infusions made from leaves and fruits and leaf decoctions are used as antidepressants; Unspecified part pressed on the head for headache; Leaves used as compress to treat headache; Fresh leaves placed on a cloth with vinegar and wrapped on the head for headache	Bhat and Jacobs (1995); Hulley and Van Wyk (2019); Nortje and van Wyk (2015); Van Wyk *et al.* (2008)
**Apiaceae**	*Alepidea amatymbica* Eckl. & Zeyh. [*A. amatymbica* var. *amatymbica* Eckl. & Zeyh., *A. amatymbica* var. *cordata* Eckl. & Zeyh., *A. aquatica* Kuntze, *Eryngium amathymbicum* (Eckl. & Zeyh.) Koso-Pol]	Giant alepidea (E); Kalmoes (A); Ikhathazo (Z); Iqwili(X); Lesoko(S)	Herb	Rhizome; roots	Dry rhizome and roots are smoked, or powdered and taken as a snuff to help prevent nervousness; Dry rhizomes are smoked or powdered and taken as snuff for mild sedation and vivid dreams; Fresh rhizomes are chewed, or decoctions are made from dried product. Also administered as snuff or burnt and inhaled. Smoke from roots used as a mild sedative	Sobiecki (2002); Van Wyk and Gericke (2000); van Wyk *et al.* (1997)
	Alepidea natalensis J.M.Wood & M.S.Evans [*A. baurii* Kuntze, *A. cilliaris* var. *baurii* Kuntze]	Natal star flower (E)	Herb	Unspecified	Unspecified	Stafford (2009)
	Alepidea pilifera Weim. [*A. ciliaris* F.Delaroche (in part)]	Lesokwana (S)	Herb	Roots; rhizomes	Roots or rhizomes used as snuff or burnt and inhaled for headaches	Moffett (2016)

	Berula erecta subsp. *thunbergii* (DC.) B.L.Burtt [*B. thunbergia* (DC.) H.Wolff]	Lesser parsnip (E); Tandpynwortel (A)	Herb	Unspecified	Infusions used as body wash for headaches	Moffett (2016)
	Centella asiatica (L.) Urb. [*C. asiatica* var. *asiatica*, *C. asiatica* var. *crista* Makino, *C. hirtella* Nannf.]	Indian pennywort(E); Inyongwane(X); Varkoortjies(A)	Herb	Leaves	Finely ground leaves used as snuff; Dried, powdered leaf used as a snuff, which produces a calming, sedative effect; Possesses anti-inflammatory, tranquilizing and age-related neuroprotective effects	Sobiecki (2002); Van Wyk and Gericke (2000); van Wyk *et al.* (1997)
	Chamarea longipedicellata B.L.Burtt	Vinkel (A)	Herb	Roots	Edible root used to help with headache	De Beer and Van Wyk (2011)
	Heteromorpha trifoliata (H.L.Wendl.) Eckl. & Zeyh. [*Bupleurum trifoliatum* H.L.Wendl. & Bartl.]	Parsley tree (E); Mkatlala (S); Umbangandlala (Z)	Tree	Leaves	Emetics and snuffed or inhaled medicines; Leaf decoctions are administered for mental and nervous diseases e.g. smoked for headaches; The Sotho administer leaf decoctions for mental and nervous diseases, and Xhosa administer warm leaf infusions for similar purposes	Hutchings (1989); Hutchings *et al.* (1996); Sobiecki (2002)
	Heteromorpha arborescens var. *abyssinica* (Hochst. ex A.Rich.) H.Wolff [*H. abyssinica* Hochst. ex A.Rich.]	Wild parsley tree (E); Wildepietersielie, pietersieliebos (A); Umbangadlala (Z); Makatlala (S)	Tree	Leaves	Leaves are smoked to relieve headache	Moffett (2016)
**Apocynaceae**	*Acokanthera oppositifolia* (Lam.) Codd [*A. longifolia* Stapf]	Bushman’s poison (E); Boesmansgif (A); Inhlungunyembe (Z); Intlungunyembe (X)	Tree	Roots	Powder made from the dry roots is used as snuff for headache; Unspecified	Bhat and Jacobs (1995); Stafford (2009)

	Asclepias crispa P.J.Bergius [*Xysmalobium crispum* (P.J.Bergius) D. Dietr.]	Bitter root (E); Bitterwortel (A)	Herb	Roots	Roots used as snuff for headache	Nortje and van Wyk (2015)
	Gomphocarpus fruticosus (L.) W.T.Aiton [*Asclecias fruticosa* L., *G. fruticosus* subsp. *decipiens* (N.E.Br) Goyder & Nicholas, *G. fruticosus* subsp. *flavidus* (N.E.Br) Goyder & Nicholas, *G. fruticosus* subsp. *rostratus* (N.E.Br) Goyder & Nicholas]	Milkweed(E); Tontelbos(A); Lebejana(S); Umsinga-lwesalukazi(Z)	Herb	Whole plant	Emetics and snuffed or inhaled medicines; Dried aerial parts used as snuff; Leaves are taken orally as headache treatment; Roots used as snuff to treat headache; Snuff made from powdered leaves used as a sedative; Unspecified; Snuff made from powdered leaves is used as a sedative; Snuff from powdered leaves is used as a sedative	Hutchings (1989); Moffett (2016); Mogale *et al.* (2019); Nortje and van Wyk (2015); Stafford (2009); Van Wyk and Gericke (2000); van Wyk *et al.* (1997)
	Hoodia gordonii (Masson) Sweet ex Decne. [*Scytanthus gordonii* (Masson) Hook., *Stapelia gordonii* Masson]	Bushman's hat, Hoodia (E); Bitterghaap (A); Khobab (K)	Shrub	Unspecified	Unspecified	van Wyk *et al.* (1997)
	Hoodia grandis (N.E. Br.) Plowes	Groothaap (A)	Shrub	Unspecified	Used for the treatment of headache	Hulley and Van Wyk (2019)
	Mondia whitei (Hook.f.) Skeels [*Chlorocodon whitei* Hook. f., *C. whiteii* Hook. f.]	White's ginger (E); Umondi (Z)	Herb	Roots	Root infusions used to treat stress and tension in adults; Unspecified	Sobiecki (2002); Stafford (2009)
	Pachycarpus inconstans N.E. Br.	None	Herb	Unspecified	Used as emetics and snuffed or inhaled medicines	Hutchings (1989)

	Pachycarpus rigidus E.Mey. ex Eckl. & Zeyh. [*Asclepias rigida* (E.Mey. ex Eckl. & Zeyh.) Schltr., *Xysmalobium rigidum* (E.Mey.) D. Dietr.]	Ishongwe (E); Lerakampjhane (S)	Herb	Rootstock	Powdered rootstock used for headache and to induce sneezing	Moffett (2016)
	Rauvolfia caffra Sond. [*R. microphylla* Stapf, *R. natalensis* Sond.]	Quinine tree (E); Umhlambamase (X)	Tree	Bark, leaves	Bark is used by traditional healers as a tranquilliser for patients believed to be possessed by spirits; Bark decoction used for insomnia and dried leaves are used as a snuff for headache	Sobiecki (2002); Van Wyk and Gericke (2000)
	Xysmalobium undulatum (L.) W.T.Aiton [*Asclepias ciliata* Murray ex Decne., *A. leucotrica* Schltr., *A. undulata* L., *Gomphorcarpus undulatus* (L.) Schltr.]	Milk bush (E); Bitterhout/melkbos (A); Iyeza (X); Ishinga (Z); Leshokoa (S)	Herb	Roots	Emetics and snuffed or inhaled medicines; Unspecified; Used as decongestant and for headache; Roots contain several glycosides with weak central nervous system depressant and antidepressant activity; Unspecified; Powdered root used as snuff	Hutchings (1989); Hutchings *et al.* (1996); Moffett (2016); Sobiecki (2002); Stafford (2009); van Wyk *et al.* (1997)
**Araceae**	*Zantedeschia aethiopica* (L.) Spreng. [*Otosma aethiopica* (L.) Raf., *Psedohomalomena pastoensis* A.D.Hawkes]	White or common arum-lily (E); Wit varkoor (A); Intebe (X); Ihlukwe (Z)	Shrub	Leaves	Leaf compressed on the head for headache	Hulley and Van Wyk (2019)
**Asparagaceae**	*Bowiea volubilis* Harv. [*Ophiobostryx volubilis* (Harv.) Skeels, *Schizobasopsis volubilis* (Harv.) J.F.Macbr.]	Climbing onion (E); Knolklimop (A); Ugibisisila, iguleni, (Z); Umgaqana (X)	Herb	Bulb	Emetics and snuffed or inhaled medicines; Infusions made from crushed bulbs are used as protective washes when travelling; Bulb used to treat sore eyes and headache; Unspecified	Hutchings (1989); Hutchings *et al.* (1996); Philander (2011); van Wyk *et al.* (1997)

	Drimia uniflora J.C.Manning &Goldblatt [*Litanthus pussilus* Harv.]	TomThumb hyacinth (E); Kleunduimpie-hiasint (A); Kgohoyalefika (S)	Shrub	Unspecified	Burnt, powdered and rubbed into cuts on forehead to cure headache; Medicinal plant used to treat headache	Moffett (2016); Moteetee *et al.* (2019)
**Asteraceae**	*Afroaster hispida* (Thunb.) J.C.Manning & Goldblatt [*Aster bakerianus* Burtt Davy ex C.A.Sm., *A. asper* (Less.) Schönland, *A. bakerianus* subsp. *albiflorus* W.Lippert]	Baker's wild aster (E); Udlutshana (Z); Umthekisana (X); Phoa (S)	Herb	Roots	Emetics and snuffed or inhaled medicines; Ground roots are taken as snuff for headaches; Dried, powdered roots taken as snuff or decoctions taken orally for headache**;** Dried, powdered roots taken as snuff	Hutchings (1989); Hutchings *et al.* (1996); Moffett (2016); van Wyk *et al.* (1997)
	Artemisia afra Jacq. ex Willd. [*A. tenuifolia* Moench]	African wormwood (E); Wilde-als (A); Umhlonyane (X); Mhlonyane (Z); Lengana (B)	Shrub	Leaves	Leaves used in the treatment of headache and anxiety; Infusions or steam from crushed leaves are commonly inhaled for headaches and colds; Unspecified; Tea made from leaves used to treat headache; Unspecified	Hulley and Van Wyk (2019); Hutchings *et al.* (1996); Stafford (2009); Thring and Weitz (2006); van Wyk *et al.* (1997)
	Artemisia dracunculus L. [*A. dracunculoides* Pursh]	True tarragon, biting dragong (E)	Herb	Unspecified	Unspecified	Stafford (2009)
	Dicerothamnus rhinocerotis (L.f.) Koek. [*Elytropappus rhinocerotis* (L.f.) Less.]	Rhinoceros bush (E); Rhenosterbos (A)	Shrub	Leaves	Used as emetics and snuffed or inhaled medicines; Leaves placed in vinegar or brandy for headache	Hutchings *et al.* (1996); Thring and Weitz (2006)
	Euryops evansii Schltr. [*E. dieterleniae* J.M.Wood]	Sehlakoana (S)	Shrub	Stem	Stem burnt and smoke as a cure for headache	Moffett (2016)
	Felicia muricata (Thunb.) Nees [*Felicia frutescens*	Wild aster, Karoo aster (E); Taai-Astertjie, Karoobossie (A); Mosala-tsela (S)	Herb	Unspecified	Used to relieve headaches	Moffett (2016)

	R.E.Fr, *Aster muricatus* Thunb.]					
	Gazania krebsiana Less. (*G. mucronata* DC.]	Terracotta gazania (E); Gousblom, roois gazania (A)	Herb	Whole plant	Plant crushed and mixed with water to treat headache	Moffett (2016)
	Gazania krebsiana subsp. *serrulata* (DC.) Roessler [*G. montana* Spreng., *G. serrulata* DC., *Meridiana serrulata* (DC.) Kuntze]	Grassland gazania (E); Kleingousblommetjie, wutgousblom (A)	Herb	Whole plant	Plant crushed and mixed with water to treat headache	Moffett (2016)
	Gazania linearis (Thunb.) [*G. krausii* Sch.Bip., *G. liunearis* var. *linearis* (Thunb.) Druce]	Treasure flower (E)	Herb	Whole plant	Plant crushed and mixed with water to treat headache	Moffett (2016)
	Helichrysum cymosum (L.) D.Don [*Gnaphakium cernuum* Thunb., *G. spadiceum* Lam.]	Silver moon (E)	Shrub	Leaves	Fresh leaves are boiled in water and the vapour used as vapour bath for treating headache	Bhat and Jacobs (1995)
	Helichrysum nudifolium (L.) Less [*H. velatum* Moeser]	Hottentot's tea (E); Hottentotstee (A); Icholocholo (Z, X); Letapiso, mohlomela-tsie-oa-thaba (S)	Herb	Leaves	Smoke from burned leaves inhaled to treat headache; Used to treat headache	Hutchings *et al.* (1996); van Wyk et al. (1997)
	Helichrysum ododratissimum (L.) Sweet [*H. ododratissimum* var. *ododratissimum* (L.) Sweet]	Most fragrant helichrysum (E); Kooigoed (A); Imphepho (Z, X); Phefo (S)	Herb	Unspecified	Used for fever and headache; Most popular species for use as ritual incense ("imphepho") and used to treat headaches, insomnia, tension and insanity	Moffett (2016); van Wyk *et al.* (1997)

	Helichrysum patulum (L.) D.Don [*Helichrysum crispum* (L.) D.Don]	Honey everlasting (E); Kooigoed (A); Impepho (Z, X); Phefo (S)	Shrub	Leaves	Small amounts of leaves are placed under the pillow for sleeplessness	Thring and Weitz (2006)
	Hilliardiella hirsuta H.Rob. [*Vernonia flanaganii* (E.P. Phillips) O.M. Hilliard, *V. hirsuta* (DC.) Sch.Bip. ex Walp., *V. hirsuta* var. *hirsuta*]	Quilted-leaved Vernonia (E); Wildesosoekertjie (A); Ikhambilenyongo (Z); Phefo-e-kholo (S)	Shrub	Stems; stalks	Used as emetics and snuffed or inhaled medicines; Powder from ground, dried stems and stalks used as snuff for headaches	Hutchings (1989); Hutchings *et al.* (1996)
	Lopholaena coriifolia (Sond.) E.Phillips & C.A.Sm. [*L. bainesii* (Oliv. & Hiern) S.Moore, *L. randii* S.Moore]	Leather-leaved fluffbush (E); Pluisiesbos (A); Mokorokorwane (B)	Shrub	Branches	Dried branches are burnt, and smoke is inhaled to treat headache	Mogale *et al.* (2019)
	Mikania capensis DC. [*M. oxyota* DC., *M. thunbergioides* Bojer ex DC.]	Cape mikania (E)	Shrub	Leaves	Used as emetics and snuffed or inhaled medicines; Leaves are sniffed for headaches	Hutchings (1989); Hutchings *et al.* (1996)
	Mikania natalensis DC.	Natal mikania (E); Ihlozi, umdlonzo (Z)	Shrub	Leaves	Used as emetics and snuffed or inhaled medicines; Leaves are sniffed for headaches	Hutchings (1989); Hutchings *et al.* (1996)
	Nolletia ciliaris (DC.) Steetz [*Leptothamnus ciliaris* DC.]	Kaalriekte (A); Mollo-wa-thaba, moloka (S)	Herb	Leaves	Leaves smoke to cure headache	Moffett (2016)
	Oncosiphon piluliferum (L.f.) Källesjö [*Pentzia pilulifera* (L.F.) Fourc.]	Globe chamomile (E); Gansogie (A)	Herb	Leaves	Leaves compressed on the head for headache	Hulley and Van Wyk (2019)

	Pegolettia baccharidifolia Less. [*Carphopappus baccharidifolius* (Less.) Sch.Bip.]	Gwarrieson (A)	Shrub	Unspecified	Used to treat headache	Hulley and Van Wyk (2019)
	Pluchea scabrida DC. [*Conyza scabrida* (DC.) DC. ex Miq]	Oven bush (E); Bakbos (A); Mokotedi-wa-thaba (S)	Shrub	Leaves	Unspecified parts used to treat headache; In Transkei, ground leaves are snuffed for headaches; Roots are used to treat depression; Leaves are placed on cloth with vinegar/brandy and wrapped around head for headache; Unspecified; Leaves placed in cloth with vinegar/brandy and wrapped around the head to treat headache	Hulley and Van Wyk (2019); Hutchings *et al.* (1996); Mogale *et al.* (2019); Moteetee *et al.* (2019); Thring and Weitz (2006); van Wyk *et al.* (1997); Van Wyk *et al.* (2008)
	Senecio pterophorus DC. [*S. pterophorus* var. *apterus* (DC.) Harv., *S. pterophorus* var. *pterophorus*]	African daisy (E); Perdegifbos (A)	Shrub	Unspecified	Used medicinally to treat headache	Philander (2011)
	Senecio speciosus Willd. [*S. concolor* DC., *S. concolor* var. *concolor*]	Beautiful senecio (E); Ibohlololo (Z); Sebea-mollo-se-senyane (S); Indambiso (X)	Herb	Leaves	Used as emetics and snuffed or inhaled medicines; Powder from ground, dried leaves sniffed for headache	Hutchings (1989); Hutchings *et al.* (1996)
	Tarchonanthus camphoratus L. [*T. camphoratus* var. *camphoratus*, *T. abyssinicus* Sch.Bip.]	Camphor bush (E); Kankerbos (A); Igqeba-elimhlophe (Z); Sefahla (S)	Tree	Branches; leaves	Sothos use smoke from burning green branches as an inhalant for headaches; Infusions of leaves and twigs used to treat headache; Branches are burnt, and smoke inhaled for the relief of headache	Hutchings *et al.* (1996); Moffett (2016); Venter and Venter (2016)

**Brassicaceae**	*Lepidium schinzii* Thell.	Pepperwort (E); Peperbossie (A); Sebitsa (S)	Herb	Leaves	Crushed leaves are used as inhalants for headaches by the Sotho; Crushed leaves are snuffed for headache	Hutchings *et al.* (1996); Moffett (2016)
**Campanulaceae**	*Lobelia anceps* L.f. [*L. alata* Labill]	Angled lobelia, swamp lobelia (E)	Herb	Unspecified	Unspecified	Stafford (2009)
**Canellaceae**	*Warburgia salutaris* (G.Bertol.) Chiov. [*W. breyeyi* R.Pott, *Chibaca salutaris* G.Bertol.]	Pepper-bark tree (E); Peperbasboom (A); Isibaha (Z); Manaka (V)	Tree	Bark	Treats headaches; Dried bark is grounded and used as snuff to treat headaches	Philander (2011); Venter and Venter (2016)
**Cannabaceae**	*Cannabis sativa* L.	Marijuana (E); Dagga (A); Umnya (X); Matekwane (S); Nsangu (Z)	Herb	All plant parts	Used in the treatment of depressive mental conditions; Whole plant is used to treat "Vaal sick" and excessive headache; Smoked to induce well-being, relaxation, sociability and/or spirituality; Administered orally, intravenously or by topical application for treatment of depression and other conditions	Hutchings *et al.* (1996); Mongalo and Makhafola (2018); Van Wyk and Gericke (2000); van Wyk *et al.* (1997)
**Cannaceae**	*Canna indica* L. [*C. edulis* Ker Gawl.]	African arrowroot, edible canna (E)	Herb	Leaves	Leaves used as a compress for headache	Hulley and Van Wyk (2019)
**Capparaceae**	*Capparis tomentosa* Lam. [*C. alexandrae* Chiov., *C. biloba* Hutch. & Dalziel, *C. floribunda* Wight]	Woolly caper bush (E); Wollerige(A); Imfihlo (X); Umabusane (Z)	Shrub	Roots	Emetics and snuffed or inhaled medicines; Roots are burnt to form a powder that is rubbed into scarifications for the relief of headache; The Zulu use unspecified parts to treat madness; Powdered, burnt roots rubbed into skin for headache	Hutchings (1989); Hutchings *et al.* (1996); Sobiecki (2002); van Wyk *et al.* (1997)

	Maerua angolensis DC. [*M. angolensis* subsp. *angolensis*]	Bead-bean tree, bead-pod tree (E); Knoppiesboontjieboom (A); Umenwayo (Z); Mogogwane (S); Mutamba-na-mme (V)	Tree	Leaves	Steam from leaves inhaled to treat headache	Venter and Venter (2016)
**Crassulaceae**	*Cotyledon orbiculata* L. [*C. ungulate* Lam.]	Pig's ear, round-leafed navel wort (E); Plakkie, varkoorblare (A)	Shrub	Unspecified	Unspecified	Stafford (2009)
	Kalanchoe brachyloba Welw. ex Britten [*K. multiflora* Schinz, *K. pyramidalis* Schönland]	Short-lobed kalanchoe (E); Gelobde plakkie (A); Tshinyanyu (V)	Shrub	Leaves	Unspecified	Sobiecki (2002)
**Dioscoreaceae**	*Dioscorea diversifolia* Griseb. [*D. multiloba* R.Knuth]	Wild yam (E); Udakwa (Z)	Tree	Tubers	Tubers are used to treat hysterical fits	Hutchings *et al.* (1996)
**Ebenaceae**	*Diospyros pubescens* Pers. [*Diospyros austroafricana* De Winter]	Fire-sticks, star-apple (E); Kritikom, jakkalsbos (A); Senokonko (S);Umbhongisa (X)	Shrub	Leaves	Smoke of burning leaves inhaled for calming and to treat headache	Van Wyk *et al.* (2008)
	Euclea divinorum Hiern [*E. stuhlmannii* Gürke]	Magic guarri, diamond-leaved euclea (E); Towerghwarrie (A); Mohlakola (S); Umhlangula (Z)	Shrub	Roots	Root infusion given as eardrops to treat headache	Van Wyk and Gericke (2000)

	Euclea undulata Thunb. [*E. humilis* Eckl. & Zeyh., *E. myrtina* Burch., *E. undulata var. myrtica* (Burch.) Hiern, *E. undulata* var. *undulata* Thunb.]	Small-leaved guarri (E); Kleinblaarghwarrie (A); Inkuzane (Z)	Shrub	Bark	Used as emetics and snuffed or inhaled medicines; Powdered bark used with a strip of *Dombeya rotundifolia* is wrapped around the head for the relief of headaches by the Sotho	Hutchings (1989); Hutchings *et al.* (1996)
**Euphorbiaceae**	*Cavacoa aurea* (Cavaco) J.Léonard [*Grossera aurea* Cavaco]	Cavaco, Natal hickory (E); Cavaco, Natal-okkerneut (A); Umbhuku (Z)	Shrub	Roots	Root infusions are taken to ease pain and for fevers	Hutchings *et al.* (1996)
	Ricinus communis L.	Castor bean, castor oil plant (E); Kastorolie (A)	Shrub	Leaves	Leaves compressed on the head for headache; Leaves are bound to sore area to alleviate pain	Hulley and Van Wyk (2019); Thring and Weitz (2006)
	Spirostachys africana Sond. [*S. synandra* (Pax) Pax, *Stillingia africana* (Sond.) Baill.]	Tamboti (E); Tambotie (A); Umthombothi (Z); Modiba (S); Morukuru (B)	Tree	Wood	Used as emetics and snuffed or inhaled medicines; Slightly burnt wood is put in the nose to relieve headaches and smoke inhaled to drive away bad spirits	Hutchings (1989); Hutchings *et al.* (1996)
	Synadenium cupulare L.C. Wheeler	Dead-man's tree (E); Gifboom (A); Umbulele (Z)	Tree	Leaves	Emetics and snuffed or inhaled medicines; Leaves are broken up and inhaled to relieve headaches; Leaves are used as medicine for headache	Hutchings (1989); Hutchings *et al.* (1996); Van Wyk and Gericke (2000)
**Fabaceae**	*Albizia adianthifolia* (Schum.) W.Wight [*A. adianthifolia* var. *adianthifolia* Schum.) W.Wight, *Mimosa adianthifolia* Schum.]	Flat-crown albizia (E); Platkroon (A); Umgadankawu (Z); Umhlandlothi (X)	Tree	Bark	Taken as snuff; Powdered bark is taken as a snuff for headaches; Powdered bark used as snuff; Bark is powdered and used as snuff for the relief of headache	Corrigan *et al.* (2011); Hutchings *et al.* (1996); van Wyk *et al.* (1997); Venter and Venter (2016)

	Albizia versicolor Oliv [*A.versicolor* var. *mossambicensis*]	Large-leaved albizia, large-leaved false thorn (E); Grootblaar-valsdoring (A); Umbhangazi (Z); Mohlabafota (S); Mutamba-pfuna (V)	Tree	Leaves	The leaves and bark are used to relieve headache	Venter and Venter (2016)
	Cassia abbreviata subsp. *beareana* (Holmes) Brenan [*Cassia beareana* Holmes]	Long-tail Cassia, wild senna (E); Sjambokpeul, peulboom (A); Molepelele (S); Muboma (V)	Tree	Branches	The smoke of burnt branches inhaled to relieve headache	Venter and Venter (2016)
	Erythrophleum lasianthum Corbishley [*E. guineense* var. *swaziense* Burtt Davy, *E. suaveolens* sensu auct.]	Swazi ordeal tree (E); Swazi-oordeelboom (A); Umbhemise (Z); Umkhanku (SS)	Tree	Bark	Used as emetics and snuffed or inhaled medicines; Powdered bark, mixed with powdered bark of *Warburgia salutaris* is widely taken as snuff for headaches	Hutchings (1989); Hutchings *et al.* (1996)
	Indigastrum fastigiatum (E.Mey.) Schrire [*Indigofera fastigiata* E.Mey.]	Slender indigo (E); Uluhlomantethe (Z); Leta-la-phofu (S)	Herb	Roots	Burnt roots smoked to cure headache; Medicinal plant used for headache	Moffett (2016); Moteetee *et al.* (2019)
	Indigofera tristis E.Mey. [*I. corniculata* E.Mey.]	Muthi mkhulu (Z); Musa-pelo (S)	Herb	Unspecified	Unspecified	Stafford (2009)
	Indigofera woodii Bolus	Wood’s indigo (E)	Shrub	Unspecified	Unspecified	Stafford (2009)
	Melolobium alpinum Eckl. & Zeyh.	Motsoehla (S)	Shrub	Unspecified	Used as a sedative to treat depression and sorrow; Unspecified parts used to comfort those who sorrow or in any case of depression	Moffett (2016); Sobiecki (2002)

	Millettia grandis (E.Mey.) Skeels[*M. caffra* Meissner]	Umzimbeet (E); Umsambeet (A); Umsimbithwa (Z); Umkunywa (X)	Tree	Unspecified	The plant is used as a tranquilliser and soporific in southern Africa	Sobiecki (2002)
	Otholobium arborescens C.H. Stirt.	Renosterveld hook-leaved pea (E); Renosterveld-vlieëkeurtjie (A)	Tree	Unspecified	Unspecified parts used for the relief of headache	De Beer and Van Wyk (2011)
	Otholobium polystictum (Harv.) C.H.Stirt., *Lotodes polysticum* (Benth. ex Harv.) Kuntze, *Psoralea polystica* Harv.]	Kite hook-leaved pea (E)	Shrub	Whole plant	Whole plants are used as traditional medicine; Medicinal plant used for headache	Hutchings *et al.* (1996); Moteetee *et al.* (2019)
	Rhynchosia caribaea Jacq.) DC. [*R. caribaea* var. *caribaea*]	Snoutbean (E); Rankboontjie (A); Isihlahlasenqomfi (Z); Monyamadi (S)	Herb	Roots	Dried, powdered roots used as snuff for headache; Medicinal plant used for headache	Moffett (2016); Moteetee *et al.* (2019)
	Schotia brachypetala Sond. [*S. semireducta* Merxm.]	Weeping boer-bean, tree fuchsia (E); Huilboerboon (A); Ihluze, umgxamu (Z); Umfofofo (X); Molope (S); Mulibi (V)	Tree	Bark	Unspecified groups use the bark to treat hangovers and nervous conditions	Sobiecki (2002)
	Sutherlandia frutescens (L) R.Br. [*S. microphylla* Burch.]	Cancer bush, balloon tree (E); Insiswa (Z); Phetola (S)	Shrub	Seeds, leaves	Seeds and leaves smoked as a dagga substitute in Namaqualand	Sobiecki (2002)
	Tephrosia capensis (Jacq.) Pers.	Cape Tephrosia (E); Pelodimaroba (S)	Shrub	Roots	Emetics and snuffed or inhaled medicines; Dried powdered roots are used as snuff to relieve headaches; Dried roots snuffed for headache; Dried powdered roots are used as a snuff for	Hutchings (1989); Hutchings *et al.* (1996); Moffett (2016); Sobiecki (2002)

					headaches and plant decoctions for nervousness	
	Vachellia erioloba (E. Mey.) P.J.H. Hurter	Camel thorn (E); Kameeldoring (A); Mogohlo (S)	Tree	Bark	Burnt and powdered bark is used as a remedy for headache	Venter and Venter (2016)
**Gentianaceae**	*Chironia baccifera* L. [*C. baccata* Hoffmanns., *C. parviflora* Salisb.]	Christmas berry (E); Bitterbossie, amberbossie (A)	Shrub	Unspecified	Infusion used to treat headache	Hulley and Van Wyk (2019)
**Gerrardinaceae**	*Gerrardina foliosa* Oliv.	Krantz-berry (E); Kransberrie (A)Umaluleka, umlulama (Z)	Shrub	Rootbark	Used as emetics and snuffed or inhaled medicines; -Rootbark is used in medicines taken for coughs, colds and headaches	Hutchings (1989); Hutchings *et al.* (1996)
**Gunneraceae**	*Gunnera perpensa* L. [*G. perpensa* var. *kilimandscharia* Schindl.	River pumpkin, wild rhubarb (E); Rivierpampoen, wilde ramenas (A); Ugobhe, ugobho (Z); Rambola-vhadzimu (V); Ighobo (X); Qobo (S)	Herb	Unspecified	Used as compress on the head to treat headache; Used for headaches	Hulley and Van Wyk (2019); Moffett (2016)
**Hypericaceae**	*Hypericum aethiopicum* Thunb.	Seeroogbossie (A); Bohoho (S)	Herb	Aerial parts	Infusion of powdered above-ground parts used as antidepressants	Moffett (2016)
	Hypericum aethiopicum subsp. *sonderi* N. Robson	Small hypericum (E); Vlieëpisbossie (A); Isimonyo (Z); Bohohwana (S)	Herb	Aerial parts	Infusion of powdered above-ground parts used as antidepressants	Moffett (2016)

	Hypericum lalandii Choisy [*H. baumii* Engl. & Gilg, *H. comorense* Baill.]	Spindly hypericum (E); Bohlokoana (S)	Herb	Unspecified	Unspecified	Stafford (2009)
	Hypericum perforatum L. [*H. vulgare* Lam., *H. perforatum* var. *petiolatum* Peterm.	Saint John's wort (E); Johanneskruid (A)	Shrub	Whole plant	Popular in the West and in South Africa for treating mild depression, anxiety and sleep disorders; Powdered extracts used as antidepressants	Sobiecki (2002); van Wyk *et al.* (1997)
	Hypericum revolutum Vahl [*H. kalmianum* Vahl, *H. revolutum* subsp. *revolutum*]	Curry bush, forest primrose (E); Kerriebos (A)	Shrub	Unspecified	Unspecified	Stafford (2009)
**Hypoxidaceae**	*Hypoxis hemerocallidea* Fisch., C.A. Mey. & Avé-Lall. [*H. elata* Hook. f., *H. obconica* Nel, *H. patula* Nel, *H. rooperi* T. Moore, *H. rooperi* var. *forbesii* Baker	Star flower, yellow star (E); Sterblom (A); Inkomfe (Z); Lotsane (S)	Shrub	Corm	Emetics and snuffed or inhaled medicines; Corm infusions are given as emetics for mental disorders; Used as charm to cure headache and for anxiety and depression; Medicinal plant used for headache; Corm infusions are used for insanity; Infusions of corms and leaves used as emetics	Hutchings (1989); Hutchings *et al.* (1996); Moffett (2016); Moteetee *et al.* (2019); Sobiecki (2002); van Wyk *et al.* (1997)
**Iridaceae**	*Gladiolus crassifolius* Baker	Thick-leaved gladiolus (E); Igulusha, ingangulazi (Z); Kgatla-e-nyenyane (S)	Herb	Unspecified	Used as a cure for headache; Medicinal plant used to treat headache	Moffett (2016); Moteetee *et al.* (2019)
	Iris domestica (L.) Goldblatt & Mabb. [*Pardanthus sinesis* Van Houtte, *Vanilla domestica* (L.) Druce, *Belamcanda chinensis*]	Leopard lily, blackberry lily (E)	Shrub	Roots	Roots are used to allay hysterical crying; The Zulu use roots to allay hysterical crying	Hutchings *et al.* (1996); Sobiecki (2002)

**Lamiaceae**	*Ballota hirsuta* Benth. [*B. africana* Colmeiro, *B. cinerea* (Desr.) Briq.]	Cape horehound (E); Katterkruie (A)	Shrub	Leaves	Leaf infusions used for treating headache; Compresses on head to treat headache; Treats headaches; Used to treat headaches; Infusions used to treat headache; Drank to treat headache	Hulley and Van Wyk (2019); Nortje and van Wyk (2015); Philander (2011); Thring and Weitz (2006); van Wyk *et al.* (1997); Van Wyk *et al.* (2008)
	Leonotis leonurus (L.) R.Br. [*Leonurus africanus* Mill., *Leonurus grandiflorus* Moench, *Leonurus superbus* Medik., *Phlomis leonurus* L., *P. speciosa* Salisb.]	Lion's ear, wild dagga (E); Wildedagga (A); Imvovo (X); Umcwili (Z)	Shrub	Whole plant	Emetics and snuffed or inhaled medicines; Cold water infusions from leaves are inhaled to relieve feverish headaches; Unspecified; Leaves are smoked for epilepsy and partial paralysis; Unspecified parts used for headache; Decoctions of flowers, stems and leaves are used to treat headache; Decoctions taken for headache	Hutchings (1989); Hutchings *et al.* (1996); Philander (2011); Sobiecki (2002); Stafford (2009); Thring and Weitz (2006); Van Wyk and Gericke (2000); van Wyk *et al.* (1997)
	Leonotis ocymifolia (Burm.f.) Iwarsson [*L. capensis* Raf., *L. dubia* E.Mey. ex Benth., *L. ocymifolia* var. *ocymifolia*]	Minaret flower, rock lion's paw (E); Klipdagga, wilde dagga (A)	Shrub	Leaves	Used to treat headache — dried, powdered leaves used as a snuff	Van Wyk *et al.* (2008)
	Mentha aquatica L.	Water mint (E); Watermint (A)	Herb	Unspecified	Unspecified	Stafford (2009)

	Mentha longifolia (L.) L. [*M. longifolia* (L.) Huds.]	Wild mint (E); Kruisement (A); Bohatsu (S); Umfuthana lomhlhanga (Z)	Herb	Leaves	Leaf infusion drank as tea and warm compress of leaves used for headache; Unspecified part compressed on the head for headache; Emetics and snuffed or inhaled medicines**;** Sothos sometimes plug their nose with crushed leaves and bind with a cloth for the relief of headaches; Used medicinally to treat headache; Unspecified parts used to treat headache; Crushed leaf infusions or decoctions drank for headache; Leaf infusion mixed with kruisement in tea for headache and general malaise	De Beer and Van Wyk (2011); Hulley and Van Wyk (2019); Hutchings (1989); Hutchings *et al.* (1996); Philander (2011); Thring and Weitz (2006); van Wyk *et al.* (1997); Van Wyk *et al.* (2008)
	Mentha spicata L. [*Mentha crispata* Schrad. Ex Willd.]	Spearmint, garden mint (E)	Herb	Leaves	Leaf infusion taken as tea to treat headache and colds	Van Wyk *et al.* (2008)
	Rosmarinus officinalis L. [*R. communis* Noronha, *R. communis* var. *communis*]	Rosemary (E)	Shrub	Unspecified	Used medicinally to treat headache	Philander (2011)
	Syncolostemon obermeyerae (M.Ashby) D.F.Otieno [*Hemizygia obermeyerae* M.Ashby]	Soutpansberg sagebush (E); Soutpansbergsalie (A)	Shrub	Unspecified	Unspecified	Stafford (2009)
**Lauraceae**	*Cassytha ciliolata* Nees [*C. capensis* Meisn., *C trifloral* E.Mey. *Ozarthris capensis* Raf.]	False dodder, devil's tresses (E); Bobbejaantou (A)	Shrub	Unspecified	Used as snuff to treat headache	Nortje and van Wyk (2015)

	Cinnamomum camphora (L.) J.Presl [*Camphora camphora* (L.) H.Karst., *Laurus camphora* L.]	Camphor laurel, camphor tree (E)	Tree	Unspecified	Unspecified	Stafford (2009)
	Cryptocarya latifolia Sond.	Broad-leafed quince, broad-leaved laurel (E); Breeblaarkweper (A); Umthugwa (Z)	Tree	Bark	Finely powdered bark infusion is taken for morning and evening cramps	Hutchings *et al.* (1996)
	Ocotea bullata (Burch.) E. Meyer in Drege [*Laurus bullata* Burch., *Oreodaphne bullata* (Burch.) Nees]	Black stinkwood (E); Stinkhout (A); Unukani (X,Z)	Tree	Bark	Emetics and snuffed or inhaled medicines; Bark used as snuff, inhaled to treat headache; South Africans use unspecified parts as an emetic for emotional and nervous disorders; Finely ground bark used as snuff for headache	Hutchings (1989); Hutchings *et al.* (1996); Sobiecki (2002); van Wyk *et al.* (1997)
**Limeaceae**	*Limeum aethiopicum* Burm. f [*L. aethiopicum* subsp. *aethiopicum, L. aethiopicum* var. *aethiopicum* Burm. f.]	Aarbossie, boesmandagga (A)	Herb	Unspecified	Smoked as a psychoactive substance	Hulley and Van Wyk (2019)
**Lycopodiaceae**	*Lycopodium clavatum* L. [*L. clavatum* subsp. *clavatum*]	Stag's-horn clubmoss, common clubmoss (E); Boribaboboholo (S)	Herb	Unspecified	Mixed with *Selaginella caffrorum* and smoked for headaches by the Sotho; Dried plant is smoked to cure headache	Hutchings *et al.* (1996); Moffett (2016)
**Malvaceae**	*Anisodontea triloba* (Thunb.) D.M.Bates [*Malva triloba* Thunb., *Malvastrum trilobium* (Thunb.) Garcke]	Wildestokroos (A)	Shrub	Leaves	Leaf infusion used to treat headache	De Beer and Van Wyk (2011)

	Dombeya rotundifolia (Hochst.) Planch. [*D. cerasiflora* Exell]	Wild pear (E); Drolpeer (A); Inhliziyonkulu (Z); Mohlabaphala (S)	Tree	Bark	The Sotho use bark with *Euclea undulata* for headaches	Hutchings *et al.* (1996)
	Hibiscus microcarpus Garke	Wild hibiscus (E); Wilde hibiscus (A); Bohojana (S)	Shrub	Unspecified	Used as a medicine for headache; Medicinal plant used for headache	Moffett (2016); Moteetee *et al.* (2019)
	Malva parviflora L. [*Althaea parviflora* (L.) Alef., *M. parviflora var. parviflora*]	Cheeseweed, little mallow (E)	Herb	Roots	Sotho give root decoctions to persons who have lost near relatives; Unspecified	Sobiecki (2002); Stafford (2009)
**Meliaceae**	*Ekebergia capensis* Sparrm. [*E. mildbraedii* Harms, *E. ruppeliana* (Fresen.) A. Rich., *E. senegalensis* Fuss]	Cape ash (E); Essenhout (A); Mmidibidi (S); Umnyamatsi (SS)	Tree	Leaves and roots	The Vha-Venda use leaves and bark in emetics and for headache; Leaves are pounded in cold water and the solution is extracted and inhaled to treat mental problems; Roots used to treat headache; Root decoction taken orally to relieve headache	Hutchings *et al.* (1996); Sobiecki (2002); van Wyk *et al.* (1997); Venter and Venter (2016)
	Melia azedarach L.	Chinaberry, persian lilac (E)	Tree	Leaves	Infusions made from a handful of leaves in half a cup of water are taken for abdominal pains	Hutchings *et al.* (1996)
	Turraea floribunda Hochst. [*Rutaea floribunda* (Hochst.) M.Roem.]	Honeysuckle tree (E); Kanferfoelieboom (A); Umdlozane (Z)	Tree	Roots	Diviners use roots to enter a neurotic state during divining dances	Sobiecki (2002)
**Melianthaceae**	*Bersama lucens* (Hochst.) Szyszył [*Natalia lucens* Hochst.]	Glossy white ash (E); Blinkblaarwitessenhout (A); Undiyaza (Z); Isindiyandiya (X)	Tree	Bark	Bark used to treat nervous disorders and headache	Philander (2011)

	Melianthus pectinatus Harv. [*M. pectinatus* subsp. *pectinatus*]	Kruidjie-roer-my-nie (A)	Shrub	Roots	Root is snuffed for headache	Nortje and van Wyk (2015)
**Menispermaceae**	*Cissampelos capensis* L.f. [*C. fruticosa* L.f.]	Dawidjieswortel (A)	Herb	Unspecified	Used for the relief of headache	Hulley and Van Wyk (2019)
**Myricaceae**	*Morella serrata* (Lam.) Killick [*Myrica serrata Lam.*]	Mountain Waxberry (E); Berg-wasbessie (A); Ulethu (Z); Umaluleka (X); Maleleka (S)	Shrub	Rootbark	Emetics and snuffed or inhaled medicines; Rootbark decoctions are taken for headaches; Rootbark used for headache	Hutchings (1989); Hutchings *et al.* (1996); Moffett (2016)
**Oleaceae**	*Olea europaea* subsp. *cuspidata* (Wall. & G.Don) Cif. [*O. europaea* subsp. *africana* (Mill.) P.S.Green., *O. chrysophylla* Lam., *O. kilimandscharica* Knobl.]	Olive tree, wild olive (E); Olienhout (A); Umnquma (Z, X); Motlhware (B); Mutlhwari (V)	Tree	Leaves	Unspecified; Infusions of dry leaves used to treat headache; Medicinal plant used for headache; Unspecified	Hutchings *et al.* (1996); Moffett (2016); Moteetee *et al.* (2019); Stafford (2009)
	Olea woodiana Knobl. [*O. mackenii* Harv., *O. woodiana* subsp. *woodiana*]	Forest olive, black ironwood (E); Bosolienhout, bosolyfboom (A); Isahlulambhazo (Z);Usintlwa (X)	Tree	Unspecified	Used as emetics and snuffed or inhaled medicines; Unspecified	Hutchings (1989); Sobiecki (2002)
**Orchidaceae**	*Ansellia africana* Lindl. [*A. africana var. australis* Summerh., *A. africana* var. *nilotica* Baker, *A. africana* subsp. *africana*]	Leopard orchid; luiperdorgidee(A); Imfeyenkawu(Z)	Herb	Unspecified	Unspecified	Sobiecki (2002)

**Papaveraceae**	*Cysticapnos pruinosa* (E.Mey. ex Bernh.) Lidén [*Corydalis pruinosa* (E.Mey. ex Bernh.) Harv.]	Wild fumaria (E); Musa pelo oa noka (S)	Herb	Unspecified	Sotho doctor use plant in drug preparation for comforting sorrowful people	Sobiecki (2002)
**Passifloraceae**	*Adenia gummifera* (Harv.) Harms [*Modecca gummifera* Harv., *A. rhodesica* Suess., *A. gummifera* var. *gummifera*]	Snake-climber, monkey rope (E); Slangklimop (A); Impinda (Z)	Shrub	Roots	Root is used to make tonic, taken orally as stimulant for seediness or depression; Infusions made from roots in boiling water are administered as emetic tonics or stimulants for seediness or depression; Unspecified parts used to treat depression	Corrigan *et al.* (2011); Philander (2011); Sobiecki (2002)
**Peraceae**	*Clutia pulchella* L. [*C. cotinifolia* Salisb., *C. pulchella* var. *genuina* Müll.Arg., *C. pulchella* var. *pulchella*, *C. gapinii* Pax]	Common lightning bush (E); Gewone bliksembos (A); Podimolwetse (S); Umsimpane (X); Umembesa (Z)	Shrub	Unspecified	Used as emetics and snuffed or inhaled medicines; Used to treat headaches; Medicinal plant used for headache	Hutchings (1989); Moffett (2016); Moteetee *et al.* (2019)
**Phyllanthaceae**	*Margaritaria discoidea* (Baill.) G.L.Webster [*Phyllanthus discoideus* (Baill.) Müll.Arg.]	Pheasant-berry, bushveld peacock-berry (E); Gewone fisantebessie (A); Isibangamlotha (Z)	Tree	Unspecified	Powders known as *umkhwangu* are used as snuff for headaches	Hutchings *et al.* (1996)
	Phyllanthus meyerianus Müll.Arg. [*Diasperus meyerianus* (Müll.Arg.) Kuntze, *P. woodii* Hutch.]	Ilethi (Z)	Herb	Rootbark	Used as emetics and snuffed or inhaled medicines; Rootbark decoctions taken for colds and headaches	Hutchings (1989); Hutchings *et al.* (1996)

	Pseudophyllanthus ovalis (E.Mey. ex Sond.) Voronts. & Petra Hoffm. [*Andrachne ovalis* (E.Mey. ex Sond.) Müll.Arg., *Savia ovalis* (E.Mey. ex Sond.) Pax & K.Hoffm.]	False lightning bush (E)	Shrub	Roots	Used as emetics and snuffed or inhaled medicines; Burnt roots are sniffed for headache; Root emetics taken to relieve morning stress and body aches and burned roots snuffed for headache	Hutchings (1989); Hutchings *et al.* (1996); Philander (2011)
**Phytolaccaceae**	*Phytolacca octandra* L. [*P. americana* var. *maxicana* L.]	Inkweed (E)	Herb	Unspecified	Unspecified	Stafford (2009)
**Piperaceae**	*Piper capense* L.f. [*P. bequaertii* De Wild.]	Wild pepper (E); Wildepepper (A); Ihlolane (Z)	Shrub	Unspecified	Unspecified	Stafford (2009)
**Plumbaginaceae**	*Plumbago auriculata* Lam. [*P. capensis* Thunb., *P. capensis* Willd.]	Cape leadwort (E); Blousyselbos (A); Umabophe (Z, X)	Shrub	Roots, leaves	Used as emetics and snuffed or inhaled medicines; Powdered roots or dried leaves taken as snuff to relieve headaches	Hutchings (1989); Hutchings *et al.* (1996)
**Poaceae**	*Cymbopogon nardus* (L.) Rendle [*C. virgatus* Stapf ex Bor, *C. validus* (Stapf) Stapf ex Burtt Davy, *Sorghum nardus* (L.) Kuntze]	Tamboekiegras (A); Isicunge/ isiqunga (Z)	Grass	Shoot, roots	Used to revitalise the nerves of moody people, Zulu use the roots and shoots to strengthen the nervous system	Sobiecki (2002)
	Imperata cylindrica (L.) Raeusch. [*I. cylindrica* var. *cylindrica*, *I. cylindrica* var. *major* (Nees) C.E.Hubb., *I. latifolia* (Hook.f.) L.Liou]	Bedding grass, cotton wool grass (E); Donsgras, lalanggras (A); Tlhorumo (B); Mohlabalerumo (S)	Grass	Unspecified	Unspecified	Moffett (2016)

**Polygalaceae**	*Securidaca longipedunculata* Fresen. [*Elsota longipendunculata* (Fresen.) Kuntze, *S. longipendunculata* var. *longipendunculata*]	Violet tree, fibre tree (E); Rooipeultjie (A); Mmaba (S); Iphuphuma (Z); Mpesu (V)	Tree	Roots; wood	Root kernel is used to treat headache; Powdered root/wood rubbed on forehead for headache	Mongalo and Makhafola (2018); van Wyk *et al.* (1997)
**Polygonaceae**	*Rumex sagittatus* Thunb. [*R. scandens* Burch., *Acetosa sagittata* Johnson & Briggs]	Climbing dock (E); Ranksuring (A); Umdende (Z); Tshitamba-tshedzi (V); Bodilaboboholo (S)	Herb	Rootstock	Emetics and snuffed or inhaled medicines; Powdered rootstock used by the Sotho as a snuff for headaches; Powdered rootstock used as snuff for headache; Used medicinally to treat headache	Hutchings (1989); Hutchings *et al.* (1996); Moffett (2016); Moteetee *et al.* (2019)
**Primulaceae**	*Rapanea melanophloeos* (L.) Mez [*R. seychellarum* Mez, *R. simensis* (Hochst. ex A. DC.) Mez, *Roemeria melanophloeos* (L.) Thunb., *Scleroxylum melanophloeum* (L.) Willd., *Sideroxylon melanophloeos* L.]	Cape beech (E); Kaapse boekenhout (A); Umaphipha (Z); Isiqwane sehlati (X)	Tree	Bark	Ground bark infusions taken by a person who feels like crying	Sobiecki (2002)
**Pteridaceae**	*Adiantum capillus-veneris* L. [*A. capillus-veneris* var. *capillus-veneris* L., *A. capillus-veneris* f. *dissectum* (M. Martens & Galeotti) Ching]	Southern maidenhair fern (E)	Herb	Leaves	Used as emetics and snuffed or inhaled medicines; Dried leaves are smoked for head and chest colds	Hutchings (1989); Hutchings *et al.* (1996)
	Pellaea calomelanos (Sw.) Link [*P. hastata* (Thunb.) Prantl, *Pteris calomelanos* Sw.]	Hard fern (E); Inkomankomo (Z); Lehorometso (S); Legogwana (B)	Shrub	Leaves	Smoke from burnt green leaves is inhaled for headaches	Hutchings *et al.* (1996)

**Ranunculaceae**	*Anemone anemonoides* (H.Rasm.) J.C.Manning & Goldblatt [*Knowltonia anemoides* H. Rasm., *Knowltonia gracilis* (Vent.) DC.]	Blistering leaves (E); Brandblare (A)	Herb	Leaves	Emetics and snuffed or inhaled medicines; Smoke from burning leaves inhaled for headache	Hutchings (1989); Hutchings *et al.* (1996)
	Anemone tenuifolia (L.f.) DC.	Cape anemone, veld anemone (E); Sybloom, veldanemoon (A)	Herb	Leaves	Leaves treat headache	Philander (2011)
	Anemone vesicatoria (L.f.) Prantl [*Knowltonia vesicatoria* (L.f.) Sims]	Blister leaf (E); Brandblaar (A)	Herb	Unspecified	Used to treat headache	Hulley and Van Wyk (2019)
	Clematis brachiata Thunb. [*C. biloba* Steud., *C. brachiata* Ker Gawl., *C. brachiate* var. *burkei* Burtt Davy]	Traveller's joy, wild clematis (E); Klimop (A); Ityolo (X); Umdlonzo (Z)	Shrub	Unspecified	Used as emetics and snuffed or inhaled medicines; Used medicinally to treat headache	Hutchings (1989); Moteetee *et al.* (2019)
	Ranunculus multifidus Forssk. [*R. striatus* Hochst. ex A. Rich., *R. udus Freyn*.]	Common buttercup (E); Botterblom, kankerblare (A); Isijojokazana (Z); Hlapi (S)	Herb	Unspecified	Emetics and snuffed or inhaled medicines; Burning plant inhaled by the Sotho people to relieve headache; Smoke is inhaled to relieve headache	Hutchings (1989); Hutchings *et al.* (1996); Moffett (2016)
**Rhamnaceae**	*Ziziphus mucronata* Willd. [*Z. madecassus* H. Pierrier, *Z. mucronata subsp. mucronata*]	Buffalo thorn (E); Blinkblaar-wag-'n-bietjie (A); Umphafa (Z); Mongalo (S)	Tree	Leaves; bark	Unspecified; Powdered leaf and bark in water is taken as an emetic	van Wyk *et al.* (1997); Venter and Venter (2016)
**Rosaceae**	*Alchemilla woodii* Kuntze [*A. wilmsii* Engl.]	Lady's mantle (E); Molalaphoka (S)	Herb	Whole plant	Whole plant is roasted, and smoke is inhaled to relieve headaches; Used medicinally to treat headache	Moffett (2016); Moteetee *et al.* (2019)

	*Prunus persica (*L.) Batsch	Peach tree, nectarine (E); Diperekisi (S)	Tree	Roots	Smoke from burning roots inhaled for headache	Moffett (2016)
	Rubus ludwigii Eckl. & Zeyh. [*R. ludwigii* subsp. *ludwigii, R. rhodacantha* E.Mey]	Bramble (E); Braambossie (A); Imencemence (Z)	Shrub	Unspecified	Unspecified	Stafford (2009)
**Rubiaceae**	*Conostomium natalense* (Hochst.) Bremek. [*C. natalense* var. *hirsuta* Baer]	Wild Pentas (E); Umbophe, ungcolozi (Z)	Shrub	Unspecified	Unspecified	Stafford (2009)
	Gallium capense subsp. *namaquense* (Eckl. & Zeyh.) Puff [*G. capense* var. *scabrum* Sond., *G. namaquense* Eckl. & Zeyh.]	Tiny-tots (E)	Herb	Roots	Root is used as snuff for headache	Nortje and van Wyk (2015)
	Gardenia volkensii subsp. *spathulifolia* (Stapf & Hutch.) Verdc.) [*G. spathulifolia* (Stapf & Hutch.)]	Bushveld gardenia, Transvaal gardenia (E); Transvaalkatjiepiering, bosveldkatjiepiering (A); Morala (S); Umgongwane (Z)	Tree	Unspecified	Infusion dropped in the eye or applied as cold compress to treat headache	Venter and Venter (2016)
**Rutaceae**	*Clausena anisata* (Wild.) Hook.f. ex Benth. [*Amyris anisata* Willd., *C. dunniana* H.Lév., *C. dunniana* var. *robusta* (Takana) C.C.Huang]	Horsewood (E); Perdepis, perdeboom (A); Umsanga (Z); Umtuto (X); Mudedede(V)	Shrub	Unspecified	Unspecified parts are used by the Xhosa to treat mental diseases; Unspecified	Sobiecki (2002); Stafford (2009)

	Ptaeroxylon obliquum (Thunb.) Radlk. [*P. utile* Eckl. & Zeyh., *Rhus obliqua* Thunb.]	Sneezewood tree (E); Nieshout (A); Umthathi (X)	Tree	Bark and wood	Emetics and snuffed or inhaled medicines; Xhosas use powdered bark traditionally as a snuff and medically to relieve headaches; Used medicinally to treat headache; Powdered bark used as snuff; Powdered wood used as snuff; Bark and wood used to make snuff to treat headache	Hutchings (1989); Hutchings *et al.* (1996); Philander (2011); Sobiecki (2002); van Wyk *et al.* (1997); Venter and Venter (2016)
	Ruta graveolens L. [*Ruta hortensis* Mill.]	Common rue, strong smelling rue (E)	Herb	Leaves	Used to treat headache; Tea made from the leaves used to treat headache	Hulley and Van Wyk (2019); Thring and Weitz (2006)
	Zanthoxylum capense (Thunb.) Harv	Small knobwood (E); Kleinperdepram (A); Umnungamabele (Z); Monokwane (S)	Tree	Unspecified	Unspecified	Stafford (2009)
**Salicaceae**	*Salix mucronata* Thunb. [*S. subserrata* Willd.]	Cape Willow (E); Kaapse Wilger (A); Mogokare (S); Umnyezane (Z); Munengeledzi (V)	Tree	Leaves; roots	Leaves are compressed on the head to treat headache; Leaf compress used for headache; Used medicinally to treat headache; Decoctions or infusions used for headache; Root decoction used to treat headache	Hulley and Van Wyk (2019); Nortje and van Wyk (2015); Philander (2011); van Wyk *et al.* (1997); Venter and Venter (2016)
**Sapindaceae**	*Dodonea viscosa* (L.) Jacq. [*D. viscosa* var. *angustifolia* (L.f.) Benth, *D. ehrenbergii* Schltdl., *D. viscosa* subsp. *angustifolia* (L.f.) J.G.West]	Sand olive (E); Sandolien, ysterbos (A); Mutata-vhana (V)	Shrub	Unspecified	Powdered and used as snuff for headache; Used as a compress for headache	De Beer and Van Wyk (2011); Nortje and van Wyk (2015)

	Hippobromus pauciflorus Radlk. [*H. alata* (Thunb.) Eckl. & Zeyh., *Rhus alatum* Thunb.]	False horsewood, bastard horsewood (E); Basterperdepisboom (A); Isiphahluka (Z); Umhlwathile (X)	Tree	Roots	Used as emetics and snuffed or inhaled medicines; Roots pounded in a little water drawn up into the nostril for headaches caused by influenza	Hutchings (1989); Hutchings *et al.* (1996)
**Sapotaceae**	*Vitellariopsis marginata* (N.E. Br.) Aubrév. [*V. sylvestris* (S. Moore) Aubrév.]	Forest bush-milkweed (E); Umphumbulu (Z)	Tree	Roots	Psychoactive medicines are made from the roots to cure moody people rendered neurotic	Sobiecki (2002)
**Scrophulariaceae**	*Aptosimum indivisum* Burch. ex Benth. [*A. nanum* Engl.]	Karoo violet, wild violet (E); Karooiooltjie, wildeviooltjie (A)	Shrub	Whole plant	Infusion of the whole plant used to treat headache	De Beer and Van Wyk (2011)
	Aptosimum spinescens (L.Bolus) F.E.Weber [*A. laricinum* Dinter]	Doringviooltjie, kankerbossie (A)	Shrub	Leaves	Dried leaves used as snuff for headache	De Beer and Van Wyk (2011)
	Diclis reptans Benth. [*D. reptans* var. *serratodentata* Kuntze]	Dwarf snapdragon, toadflax (E); Isinama (Z); Koenana (S)	Herb	Unspecified	Unspecified	Stafford (2009)
	Manulea paniculata Benth [*Sutera elliotensis* Hiern]	Bolao (S)	Herb	Leaves; shoots	Lotion from leaves and shoots used to purify heads of those who handled a corpse; Medicinal plant used to treat headache	Moffett (2016); Moteetee *et al.* (2019)
	Sutera atropurpurea Hiern [*Lyperia atropurpurea* Benth., *S. brunnea* Hirn]	Bruinsafraanbos, geelblommetjie (A)	Shrub	Unspecified	The plant is used for headache and anxiety	Sobiecki (2002)
**Selaginellaceae**	*Selaginella caffrorum* (Milde) Hieron.	Resurrection plant (E); Boriba (S)	Herb	Unspecified	Smoked for the relief of headache	Moffett (2016)

**Solanaceae**	*Datura ferox* L. [*D. laevis* Bertol. *D. quercifolia* Kunth]	Long-spined thorn apple (E); Groot stinkblaar (A)	Shrub	Unspecified	Unspecified	Stafford (2009)
	Datura metel L. [*D. metel var. dentata* Schltdl. & Cham., *D. metel var. fastuosa* (L.) Saff.]	Angel's trumpet (E)	Shrub	Unspecified	Emetics and snuffed or inhaled medicines; Unspecified parts smoked for the relief of headache; Unspecified	Hutchings (1989); Hutchings *et al.* (1996); Sobiecki (2002)
	Datura stramonium L. [*D. stramonium* var. *canescens* Roxb., *D. stramonium* var. *chalybaea* W.D.J.Koch]	Common thorn apple (E); Malpitte (A); Ijoyi, umhlabavutha (X); Iloyi (Z)	Shrub	Leaves	Unspecified part compressed on the head to relieve headache; Emetics and snuffed or inhaled medicines; Unspecified parts smoked for the relief of headache; The Venda use the leaves to treat insanity. Healers inhale powdered roots and leaves as snuff for divinatory purposes; Unspecified; Dried and powdered leaves used as consciousness-altering snuff by diviners; Leaves used to treat headache	Hulley and Van Wyk (2019); Hutchings (1989); Sobiecki (2002); Stafford (2009); Thring and Weitz (2006); Van Wyk and Gericke (2000); van Wyk *et al.* (1997)
	Iochroma parvifolium (Roem. & Schult.) D'Arcy [*Lycium horridum* Kunth]	Moferefere (S)	Shrub	Unspecified	Unspecified parts used to treat headache; Medicinal plant used to treat headache	Moffett (2016); Moteetee *et al.* (2019)
	Nicotiana glauca Graham [*N. glauca lateritia* Lillo]	Tree tobacco (E)	Shrub	Leaves	Compressed on the head for headache (external use only); Leaf compress used to treat headache; Leaves are warmed and put on the head to relieve headache; Fresh leaves applied to the head as a poultice for headache	Hulley and Van Wyk (2019); Nortje and van Wyk (2015); Van Wyk and Gericke (2000); Van Wyk *et al.* (2008)

**Thymelaeaceae**	*Gnidia capitata* L.f. [*Lasiosiphon carpitatus* (L.f.) Burtt Davy]	Kerrieblom (A); Setele (S)	Shrub	Unspecified	Used as a snuff to cure headache; Medicinal plant used to treat headache	Moffett (2016); Moteetee *et al.* (2019)
	Gnidia gymnostachya (Meisn.) Gilg [*Arthrosolen gymnostachya* (Meisn.) C.A.Mey., *Passerina gymnostachya* Meisn.]	Small gnidia (E); Thopananyana (S)	Shrub	Leaves	Leaves smoke to relieve headache; Medicinal plants used to treat headache	Moffett (2016); Moteetee *et al.* (2019)
	Gnidia kraussiana Meisn. [*G. kraussiana* var. *kraussiana*, *Lasiosiphon kraussiana* (Meisn.) Burtt Davy]	Yellow heads (E); Tlhorumo (S)	Shrub	Unspecified	Used as snuff; Medicinal plant used to treat headache	Moffett (2016); Moteetee *et al.* (2019)
**Xanthorrhoeaceae**	*Bulbine frutescens* (L.) Willd. [*B. incurva* (Thunb.) Spreng.]	Stalked bulbine, snake flower (E); Balsem kopieva, geelkatstert (A)	Shrub	Unspecified	Unspecified	Stafford (2009)
	Bulbine latifolia (L.f.) Spreng. [*Anthericum latifolium* L.f., *B. brunsvigiaefolia* Baker, *B. brunsvigiifolia* Baker, *B. ensifolia* Baker]	Broad-leaved bulbine (E); Rooiwortel (A); Incelwane (X); Ibhucu (Z)	Herb	Unspecified	Used by Zulu men and boys in purification rites for the prevention of antisocial behaviour	Sobiecki (2002)
	Gasteria croucheri (Hook.f.) Baker [*G. disticha* var. *natalensis* Baker, *Aloe croucheri* Hook.f.]	Forest Gasteria, forex ox-tounge, Natal Gasteria (E); Bosaalwee, oukossie, Natal beestong (A); Impundu (Z)	Shrub	Unspecified	Unspecified	Stafford (2009)

**Zamiaceae**	*Stangeria eriopus* (Kunze) Baill. [*Lomaria eriopus* Kunze, *S. katzeri* Regel, *S. paradoxa* T.Moore, *S. sanderiana* J.Schust., *S. schizodon* W.Bull]	Stranger's cycad (E); Bobbejaankos; Imfingo (Z); Umfingwani (X)	Shrub	Tubers	Used as emetics and snuffed or inhaled medicines; Burnt powdered underground tubers are used for headaches	Hutchings (1989); Hutchings *et al.* (1996)
**Zingiberaceae**	*Siphonochilus aethiopicus* (Schweinf.) B.L. Burtt [*S. evae* (Briq.) B.L.Burtt, *S. natalensis* (Schltr. & K.Schum.) J.M.Wood & Frank]	Natal ginger, wild ginger (E); Wildegemmer (A); Indungulo (Z)	Herb	Unspecified	Used medicinally to treat headaches	Philander (2011)
